# Epidemiology of injuries from fire, heat and hot substances: global, regional and national morbidity and mortality estimates from the Global Burden of Disease 2017 study

**DOI:** 10.1136/injuryprev-2019-043299

**Published:** 2019-12-18

**Authors:** Spencer L James, Lydia R Lucchesi, Catherine Bisignano, Chris D Castle, Zachary V Dingels, Jack T Fox, Erin B Hamilton, Nathaniel J Henry, Darrah McCracken, Nicholas L S Roberts, Dillon O Sylte, Alireza Ahmadi, Muktar Beshir Ahmed, Fares Alahdab, Vahid Alipour, Zewudu Andualem, Carl Abelardo T Antonio, Jalal Arabloo, Ashish D Badiye, Mojtaba Bagherzadeh, Amrit Banstola, Till Winfried Bärnighausen, Akbar Barzegar, Mohsen Bayati, Soumyadeep Bhaumik, Ali Bijani, Gene Bukhman, Félix Carvalho, Christopher Stephen Crowe, Koustuv Dalal, Ahmad Daryani, Mostafa Dianati Nasab, Hoa Thi Do, Huyen Phuc Do, Aman Yesuf Endries, Eduarda Fernandes, Irina Filip, Florian Fischer, Takeshi Fukumoto, Ketema Bizuwork Bizuwork Gebremedhin, Gebreamlak Gebremedhn Gebremeskel, Syed Amir Gilani, Juanita A Haagsma, Samer Hamidi, Sorin Hostiuc, Mowafa Househ, Ehimario U Igumbor, Olayinka Stephen Ilesanmi, Seyed Sina Naghibi Irvani, Achala Upendra Jayatilleke, Amaha Kahsay, Neeti Kapoor, Amir Kasaeian, Yousef Saleh Khader, Ibrahim A Khalil, Ejaz Ahmad Khan, Maryam Khazaee-Pool, Yoshihiro Kokubo, Alan D Lopez, Mohammed Madadin, Marek Majdan, Venkatesh Maled, Reza Malekzadeh, Navid Manafi, Ali Manafi, Srikanth Mangalam, Benjamin Ballard Massenburg, Hagazi Gebre Meles, Ritesh G Menezes, Tuomo J Meretoja, Bartosz Miazgowski, Ted R Miller, Abdollah Mohammadian-Hafshejani, Reza Mohammadpourhodki, Shane Douglas Morrison, Ionut Negoi, Trang Huyen Nguyen, Son Hoang Nguyen, Cuong Tat Nguyen, Molly R Nixon, Andrew T Olagunju, Tinuke O Olagunju, Jagadish Rao Padubidri, Suzanne Polinder, Navid Rabiee, Mohammad Rabiee, Amir Radfar, Vafa Rahimi-Movaghar, Salman Rawaf, David Laith Rawaf, Aziz Rezapour, Jennifer Rickard, Elias Merdassa Roro, Nobhojit Roy, Roya Safari-Faramani, Payman Salamati, Abdallah M Samy, Maheswar Satpathy, Monika Sawhney, David C Schwebel, Subramanian Senthilkumaran, Sadaf G Sepanlou, Mika Shigematsu, Amin Soheili, Mark A Stokes, Hamid Reza Tohidinik, Bach Xuan Tran, Pascual R Valdez, Tissa Wijeratne, Engida Yisma, Zoubida Zaidi, Mohammad Zamani, Zhi-Jiang Zhang, Simon I Hay, Ali H Mokdad

**Affiliations:** 1 Institute for Health Metrics and Evaluation, University of Washington, Seattle, Washington, USA; 2 Department of Anesthesiology, Kermanshah University of Medical Sciences, Kermanshah, Iran; 3 Department of Epidemiology, Jimma University, Jimma, Ethiopia; 4 Evidence Based Practice Center, Mayo Clinic Foundation for Medical Education and Research, Rochester, MN, USA; 5 Health Management and Economics Research Center, Tehran, Iran; 6 Health Economics Department, Iran University of Medical Sciences, Tehran, Iran; 7 Environmental and Occupational Health and Safety Department, University of Gondar, Gondar, Ethiopia; 8 Department of Health Policy and Administration, University of the Philippines Manila, Manila, Philippines; 9 Department of Applied Social Sciences, Hong Kong Polytechnic University, Hong Kong, China; 10 Health Management and Economics Research Center, Iran University of Medical Sciences, Tehran, Iran; 11 Department of Forensic Science, Government Institute of Forensic Science, Nagpur, India; 12 Chemistry Department, Sharif University of Technology, Tehran, Iran; 13 Department of Research, Public Health Perspective Nepal, Pokhara-Lekhnath Metropolitan, Nepal; 14 Heidelberg Institute of Global Health (HIGH), Heidelberg University, Heidelberg, Germany; 15 T.H. Chan School of Public Health, Harvard University, Boston, Massachusetts, USA; 16 Occupational Health Department, Kermanshah University of Medical Sciences, Kermanshah, Iran; 17 Health Human Resources Research Center, Department of Health Economics, School of Management & Information Sciences, Shiraz University of Medical Sciences, Shiraz, Iran; 18 The George Institute for Global Health, New Delhi, India; 19 Social Determinants of Health Research Center, Babol University of Medical Sciences, Babol, Iran; 20 Department of Global Health and Social Medicine, Harvard University, Boston, Massachusetts, USA; 21 Partners In Health, Boston, Massachusetts, USA; 22 Applied Molecular Biosciences Unit, University of Porto, Porto, Portugal; 23 Institute of Public Health, University of Porto, Porto, Portugal; 24 Division of Plastic Surgery, University of Washington, Seattle, Massachusetts, USA; 25 Institute of Public Health Kalyani, Kalyani, India; 26 School of Health Science, Orebro University, Orebro, Sweden; 27 Toxoplasmosis Research Center, Mazandaran University of Medical Sciences, Sari, Iran; 28 Department of Epidemiology, Shiraz University of Medical Sciences, Shiraz, Iran; 29 Center of Excellence in Public Health Nutrition, Nguyen Tat Thanh University, Ho Chi Minh, Vietnam; 30 Center of Excellence in Behavioral Medicine, Nguyen Tat Thanh University, Ho Chi Minh, Vietnam; 31 Public Health Department, Saint Paul’s Hospital Millennium Medical College, Addis Ababa, Ethiopia; 32 REQUIMTE/LAQV, University of Porto, Porto, Portugal; 33 Psychiatry Department, Kaiser Permanente, Fontana, California, USA; 34 School of Health Sciences, A.T. Still University, Mesa, Missouri, USA; 35 School of Public Health Medicine, Bielefeld University, Bielefeld, Germany; 36 Gene Expression & Regulation Program, Cancer Institute (W.I.A), Philadelphia, Pennsylvania, USA; 37 Department of Dermatology, Kobe University, Kobe, Japan; 38 Nursing Department, Addis Ababa University, Addis Ababa, Ethiopia; 39 Nursing Department College of Health Science, Aksum University, Aksum, Ethiopia; 40 Nursing Department, Mekelle University, Mekelle, Ethiopia; 41 Faculty of Allied Health Sciences, The University of Lahore, Lahore, Pakistan; 42 Chairman BOG, Afro-Asian Institute Lahore, Pakistan; 43 Department of Public Health, Erasmus University Medical Center, Rotterdam, The Netherlands; 44 School of Health and Environmental Studies, Hamdan Bin Mohammed Smart University, Dubai, United Arab Emirates; 45 Faculty of Dentistry, Department of Legal Medicine and Bioethics, Carol Davila University of Medicine and Pharmacy, Bucharest, Romania; 46 Clinical Legal Medicine Department, National Institute of Legal Medicine Mina Minovici, Bucharest, Romania; 47 Division of Information and Computing Technology, College of Science and Engineering, Hamad Bin Khalifa University, Doha, Qatar; 48 Qatar Foundation for Education, Science, and Community Development, Doha, Qatar; 49 School of Public Health, University of the Western Cape, Bellville, South Africa; 50 Department of Public Health, Walter Sisulu University, Mthatha, South Africa; 51 Department of Community Medicine, University of Ibadan, Ibadan, Nigeria; 52 Research Institute for Endocrine Sciences, Shahid Beheshti University of Medical Sciences, Tehran, Iran; 53 Institute of Medicine, University of Colombo, Colombo, Sri Lanka; 54 Faculty of Graduate Studies, University of Colombo, Colombo, Sri Lanka; 55 Department of Nutrition and Dietetics, Mekelle University, Mekelle, Ethiopia; 56 Hematology-Oncology and Stem Cell Transplantation Research Center, Tehran University of Medical Sciences, Tehran, Iran; 57 Hematologic Malignancies Research Center, Tehran University of Medical Sciences, Tehran, Iran; 58 Department of Public Health and Community Medicine, Jordan University of Science and Technology, Ramtha, Jordan; 59 Department of Global Health, University of Washington, Seattle, Washington, USA; 60 Epidemiology and Biostatistics Department, Health Services Academy, Islamabad, Pakistan; 61 Department of Public Health, School of Health, Mazandaran University of Sciences, Sari, Iran; 62 Department of Preventive Cardiology, National Cerebral and Cardiovascular Center, Suita, Japan; 63 University of Melbourne, Melbourne, Victoria, Australia; 64 Pathology Department, College of Medicine, Imam Abdulrahman Bin Faisal University, Dammam, Saudi Arabia; 65 Department of Public Health, Trnava University, Trnava, Slovakia; 66 Health Education and Research Department, SDM College of Medical Sciences & Hospital, Dharwad, India; 67 Health University, Rajiv Gandhi University of Health Sciences, Bangalore, India; 68 Digestive Diseases Research Institute, Tehran University of Medical Sciences, Tehran, Iran; 69 Non-communicable Diseases Research Center, Shiraz University of Medical Sciences, Shiraz, Iran; 70 Ophthalmology Department, Iran University of Medical Sciences, Tehran, Iran; 71 Ophthalmology Department, University of Manitoba, Winnipeg, Manitoba, Canada; 72 Plastic Surgery Department, Iran University of Medical Sciences, Tehran, Iran; 73 Public Risk Management Institute, Mississauga, Ontario, Canada; 74 Trade and Competitiveness, World Bank, New York city, New York, USA; 75 Mekelle University, Mekelle, Ethiopia; 76 Forensic Medicine Division, Department of Pathology, College of Medicine, Imam Abdulrahman Bin Faisal University, Dammam, Saudi Arabia; 77 Breast Surgery Unit, Helsinki University Hospital, Helsinki, Finland; 78 Center for Innovation in Medical Education, Pomeranian Medical University, Szczecin, Poland; 79 Pacific Institute for Research & Evaluation, Calverton, Maryland, USA; 80 School of Public Health, Curtin University, Perth, Western Australia, Australia; 81 Department of Epidemiology and Biostatistics, Shahrekord University of Medical Sciences, Shahrekord, Iran; 82 Department of Nursing, Shahroud University of Medical Sciences, Shahroud, Iran; 83 Department of Surgery, University of Washington, Seattle, Washington, USA; 84 General Surgery Department, Carol Davila University of Medicine and Pharmacy, Bucharest, Romania; 85 Emergency Hospital of Bucharest, Carol Davila University of Medicine and Pharmacy, Bucharest, Romania; 86 Institute for Global Health Innovations, Duy Tan University, Hanoi, Vietnam; 87 Department of Psychiatry and Behavioural Neurosciences, McMaster University, Hamilton, Ontario, Canada; 88 Department of Psychiatry, University of Lagos, Lagos, Nigeria; 89 Department of Pathology and Molecular Medicine, McMaster University, Hamilton, Ontario, Canada; 90 Forensic Medicine and Toxicology Department, Manipal Academy of Higher Education, Mangaluru, India; 91 Department of Chemistry, Sharif University of Technology, Tehran, Iran; 92 Biomedical Engineering Department, Amirkabir University of Technology, Tehran, Iran; 93 College of G raduate Health Sciences, A.T. Still University, Mesa, Arizona, USA; 94 Medichem, Barcelona, Spain; 95 Sina Trauma and Surgery Research Center, Tehran University of Medical Sciences, Tehran, Iran; 96 Department of Primary Care and Public Health, Imperial College London, London, UK; 97 Academic Public Health Department, Public Health England, London, UK; 98 WHO Collaborating Centre for Public Health Education and Training, Imperial College London, London, UK; 99 University College London Hospitals, London, UK; 100 Surgery Department, University of Minnesota, Minneapolis, Minnesota, USA; 101 Surgery Department, University Teaching Hospital of Kigali, Kigali, Rwanda; 102 Public Health Department, Wollega University, Nekemte, Ethiopia; 103 Public Health Department, Addis Ababa University, Addis Ababa, Ethiopia; 104 Public Health Planning and Evidence Practice Area, National Health Systems Resource Centre, New Delhi, India; 105 Department of Public Health Sciences, Karolinska Institutet, Stockholm, Sweden; 106 Faculty of Public Health, Kermanshah University of Medical Sciences, Kermanshah, Iran; 107 School of Health and Policy Management, Faculty of Health, York University, Toronto, Ontario, Canada; 108 Department of Entomology, Ain Shams University, Cairo, Egypt; 109 UGC Centre of Advanced Study in Psychology, Utkal University, Bhubaneswar, India; 110 Udyam-Global Association for Sustainable Development, Bhubaneswar, India; 111 Department of Public Health Sciences, University of North Carolina at Charlotte, Charlotte, North Carolina, USA; 112 Department of Psychology, University of Alabama at Birmingham, Birmingham, Alabama, USA; 113 Emergency Department, Manian Medical Centre, Erode, India; 114 National Institute of Infectious Diseases, Tokyo, Japan; 115 Medical Surgical Nursing Department, Urmia University of Medical Science, Urmia, Iran; 116 Emergency Nursing Department, Zanjan University of Medical Sciences, Zanjan, Iran; 117 Department of Psychology, Deakin University, Burwood, Victoria, Australia; 118 HIV/STI Surveillance Research Center and WHO Collaborating Center for HIV Surveillance, Institute for Futures Studies in Health, Kerman University of Medical Sciences, Kerman, Iran; 119 Department of Epidemiology and Biostatistics, School of Public Health, Tehran University of Medical Sciences, Tehran, Iran; 120 Department of Health Economics, Hanoi Medical University, Hanoi, Vietnam; 121 Argentine Society of Medicine, Buenos Aires, Argentina; 122 Velez Sarsfield Hospital, Buenos Aires, Argentina; 123 Department of Psychology and Counselling, University of Melbourne, Melbourne, Victoria, Australia; 124 Department of Medicine, University of Melbourne, St Albans, Victoria, Australia; 125 School of Allied Health Sciences, Addis Ababa University, Addis Ababa, Ethiopia; 126 Department of Epidemiology, University Hospital of Setif, Setif, Algeria; 127 Student Research Committee, Babol University of Medical Sciences, Babol, Iran; 128 Department of Preventive Medicine, Wuhan University, Wuhan, China; 129 Department of Health Metrics Sciences, School of Medicine, University of Washington, Seattle, Washington, USA

**Keywords:** burn, descriptive epidemiology, burden of disease

## Abstract

**Background:**

Past research has shown how fires, heat and hot substances are important causes of health loss globally. Detailed estimates of the morbidity and mortality from these injuries could help drive preventative measures and improved access to care.

**Methods:**

We used the Global Burden of Disease 2017 framework to produce three main results. First, we produced results on incidence, prevalence, years lived with disability, deaths, years of life lost and disability-adjusted life years from 1990 to 2017 for 195 countries and territories. Second, we analysed these results to measure mortality-to-incidence ratios by location. Third, we reported the measures above in terms of the cause of fire, heat and hot substances and the types of bodily injuries that result.

**Results:**

Globally, there were 8 991 468 (7 481 218 to 10 740 897) new fire, heat and hot substance injuries in 2017 with 120 632 (101 630 to 129 383) deaths. At the global level, the age-standardised mortality caused by fire, heat and hot substances significantly declined from 1990 to 2017, but regionally there was variability in age-standardised incidence with some regions experiencing an increase (eg, Southern Latin America) and others experiencing a significant decrease (eg, High-income North America).

**Conclusions:**

The incidence and mortality of injuries that result from fire, heat and hot substances affect every region of the world but are most concentrated in middle and lower income areas. More resources should be invested in measuring these injuries as well as in improving infrastructure, advancing safety measures and ensuring access to care.

## Introduction

Burns and other injuries caused by exposure to fire, heat and hot substances can be severely disabling and can cause death even in the presence of healthcare services. While prevention of these injuries and safety programmes are integral to averting burden, it is also important to consider the role of medical services. The severity spectrum of such injuries may necessitate complex treatment by burn surgeons and intensive care services that are not reliably available in all areas of the world. When it comes to injuries such as burns, medical and surgical care advents such as split-thickness skin grafting in select anatomical locations, fluid and electrolyte repletion, and infection control have emerged as critical elements of care for victims of these injuries, in some scenarios mitigating risk of death and long-term disability.^1^ Comprehensive medical infrastructure for treating burn injuries is therefore a critical component of a healthcare system. Given the care requirements that severe injuries can demand, a detailed epidemiological assessment of the morbidity and mortality that can result from fire, heat and hot substances is important. Moreover, these injuries may become increasingly topical as there is increasing emphasis on ensuring that all areas of the world have access to standard-of-care interventions that have led to improved patient outcomes across various domains of illness and injury in resource-rich settings.^1^

Previous epidemiological research, which largely focuses on burns as opposed to all injuries that can result from fire, heat and hot substances, has found that the burden of morbidity and mortality from these injuries is distributed unevenly across income groups, with higher burden in lower income regions of the world but with persistent incidence and mortality even in higher income areas. Using mortality data from the WHO and economic data from the World Bank, Peck and Pressman explored the relationship between income and burn injuries, showing that these injuries presented the greatest burden in low and middle-income countries.^2^ There is some consensus in the literature that, with some exceptions, overall incidence and mortality are trending towards global declines, and several studies have concluded that still further decreases should be possible by increasing socioeconomic status, improving working conditions and launching focused safety awareness campaigns.^2 3^ Some country-specific increases in morbidity and mortality have been attributed to issues with injury documentation biases as well as increasing admission rates to burn centres as more patients seek treatment for minor injuries.^3^ Other country assessments, such as in Iran, have examined trends by age, sex, urbanicity and injury circumstances in order to make sense of trends over time.^4 5^ However, it is unclear whether these trends have continued in more recent years and in specific geographies. Changing population structures may also affect the morbidity and mortality risk of these injuries, as children under 4 years of age and adults over 60 years of age have been shown to have the highest rates of burn injuries and burn-related deaths.^6^ Injuries of this nature in younger populations are particularly concerning given the long period of disability that can follow. Hyder and colleagues conducted an injury surveillance study in four low and middle-income countries and found 17% of children with burn injuries experienced disability lasting longer than 6 weeks and 8% were estimated to have lifelong disability due to their burn injury.^7^ These findings point to the importance of comprehensive measurement of the burden of burns across countries, ages, sexes and time in helping to guide safety efforts, prevention interventions and resource planning.

Existing research on this topic is limited in several respects. These include data sparsity in low and middle-income countries where burden is estimated to be highest. Changes in criteria for and behaviour in seeking treatment from burn centres for injuries have led to difficulties in comparing data over time. In this paper, we aimed to address these limitations in three ways. First, we sought to provide updated estimates for all countries, including low and middle-income countries, using all available data sources with fatal and non-fatal cases. Second, we used covariates to help improve estimation in data-sparse locations. And, third, we generated estimates using modern spatiotemporal statistical modelling techniques.^8^ We also aimed to further explore the time trends of burn injuries across the socioeconomic development spectrum and to produce estimates for morbidity and mortality across all global locations, age groups, sexes and over time.

A worldwide network of collaborators contribute to the Global Burden of Disease (GBD) study, a comprehensive assessment of hundreds of diseases, injuries and risk factors. Using annually updated data and methods, the study produces estimates of all-cause mortality, causes of death (cause-specific mortality rates, years of life lost (YLL)), non-fatal health outcomes (ie, incidence, prevalence and years lived with disability (YLD)) and risk factors. These estimates are calculated across a range of years for all age groups, sexes and 195 countries and territories, grouped into 21 regions and seven super-regions. This level of estimation detail permits more granular analyses of morbidity and mortality across demographics, geographies and causes and nature of injuries.

To investigate the morbidity and mortality caused by fire, heat and hot substances, we used the GBD 2017 framework and findings. We then explored several themes that emerged from this investigation.

## Methods

### GBD 2017 study

The GBD 2017 study methods and results are described in extensive detail elsewhere, including description of the analytical estimation framework used to measure deaths, YLLs, incidence, prevalence, YLDs and disability-adjusted life years (DALY).^8–13^ A summary overview of the GBD study including key statistical methods is provided in online supplementary appendix 1. What follows is a description of the methodological components specific to injuries and fire, heat and hot substance estimation within the GBD framework.

10.1136/injuryprev-2019-043299.supp1 Supplementary data

### GBD injury classification

The GBD cause hierarchy categorises both the cause of injuries and the nature of injuries. External cause-of-injury categories or ‘codes’ are used for incidents such as falls and road injuries, as well as for fire, heat and hot substances. External cause-of-injury codes are mutually exclusive and collectively exhaustive within the injury estimation process. Nature of injury codes in turn categorise the injuries that result from an external cause, for example, the surface burn that can occur in a fire. Nature of injury codes group injuries into 47 mutually exclusive and collectively exhaustive categories, quantifying the various disabling outcomes of each cause of injury according to the International Classification of Diseases (ICD), specifically chapters S and T in ICD-10 and codes 800–999 in ICD-9. More information on nature of injury groups is provided in GBD literature.^8^

### Mortality and YLLs from fire, heat and hot substances

The approach for modelling and estimating causes of death including deaths from fire, heat and hot substances is described in related GBD publications.^14^ A brief overview of this process is as follows. First, all available data sources for cause of death were accessed and mapped into the GBD cause list. Sources used for modelling fire, heat and hot substances include vital registration records, verbal autopsies, mortality surveillance, censuses, surveys, hospital data and mortuary data. For fire, heat and hot substances, we used ICD-9 codes E890–E899 and E924 and ICD-10 codes X00–X06.9 and X08–X19.9. Some sources, particularly verbal autopsy studies, typically do not codify cause of death using the ICD classification system and instead use custom cause of death categories; in these cases, the corresponding causes were mapped to the GBD cause list (eg, ‘fires’ as a cause in a verbal autopsy study would map to fire, heat and hot substances in GBD). Ill-defined causes of death are then redistributed to different causes, including fire, heat and hot substances via a process known as garbage code redistribution, which is conducted for each age, sex, country or territory, year and ICD type.^15 16^ This process allows for an individual who dies later from such an injury to still be included in the cause-specific mortality process.

After cause mapping, models for fire, heat and hot substances were conducted using the GBD Cause of Death Ensemble modelling (CODEm) method to produce estimates by age, sex, country, year and cause. CODEm builds an optimised cause of death model based on testing a large variety of possible models to estimate trends in causes of death using an algorithm to select varying combinations of covariates that are run through several modelling classes.^17^ An ensemble of best performing models is then created based on measured performance in out-of-sample validity testing. We also used covariates in these models, which included lag-distributed income per capita, education per capita in years, alcohol use in litres per capita, an indicator for opium cultivation, population density over 1000/km^2^, a summary exposure value for violent injuries, Socio-demographic Index (SDI) and Healthcare Access and Quality index.^18^ Deaths from each cause are then scaled to fit the overall mortality estimate for each demographic and location group in GBD.

YLLs due to fire, heat and hot substances are calculated by multiplying deaths by the residual life expectancy at the age of death as derived from the GBD 2017 reference model life table. YLLs are intended to show how many years of life are lost when a death occurs at an age less than the life expectancy; for example, if the life expectancy is 80 and a death occurs at age 5, then 75 years of life were lost.

### Incidence, prevalence and YLDs due to fire, heat and hot substances

The approach to estimate non-fatal injury outcomes (incidence, prevalence and YLDs) in GBD is provided in related publications.^8 19^ A summary of these methods for fire, heat and hot substances is as follows. We applied DisMod-MR V.2.1 (an epidemiological meta-regression tool) to incidence data for fire, heat and hot substances from emergency department and hospital records and survey data to estimate inpatient and outpatient incidence by location, year, age and sex. This modelling process also used cause-specific mortality rates to guide model fits in data-sparse areas. We use a prior to assume that case fatality rates are higher in lower income settings by adding lag-distributed income per capita as a covariate on excess mortality, which causes a negative relationship between income and mortality. To determine the disability experienced by victims of fire, heat and hot substance injuries who experience multiple injuries, we developed a hierarchy to select the nature of injury that would lead to the largest long-term burden when an individual experiences multiple injuries. This calculation uses a combination of likelihood of long-term disability and the corresponding GBD disability weights, which are described in more detail in the GBD literature.^19^ The severity hierarchy was established using pooled data sets of follow-up studies from China, the Netherlands and the USA, where health status 1 year after injury could be mapped to existing GBD disability weights.^20–26^

Since injury disability is related to nature of injury (eg, burns covering more than 20% of the body or a digit amputation) rather than cause of injury (eg, exposure to a house fire as opposed to a road injury leading to a burn), we estimated the proportion of each cause-of-injury category, including fire, heat and hot substances, that results in a particular nature of injury category (eg, burns or digit amputation). These proportions were estimated using a Dirichlet regression method based on proportions of cause-nature combinations measured in dual-coded (ie, both cause of injury and nature of injury coded) hospital and emergency department data sets and data used elsewhere in GBD estimation as well as from the National Injury Surveillance System in China.^8,27^ Applying these matrices to our cause-of-injury incidence from DisMod-MR V.2.1, we produced incidence of injury warranting hospital admission and incidence of injury warranting other healthcare by cause and nature of injury. We then estimated short-term disability for each nature‐of‐injury category in the fire, heat and hot substance cause-of-injury category based on average duration for treated cases for each nature of injury and for inpatient and outpatient injuries from the Dutch Injury Surveillance System.^23^ Then, we estimated proportions of permanent disability (ie, longer than 1 year) for each nature of injury category using long-term follow-up studies.^20–26^ Then, we applied the ordinary differential equation solver used in the DisMod-MR V.2.1 computational engine to estimate the long-term prevalence for each nature of injury category from incidence and the long-term mortality risk for each nature of injury, since some nature of injury categories increase mortality risk above the background mortality rate. YLDs were calculated as the product of the prevalence of a health state and a disability weight, adjusted for comorbidity with other diseases and injuries as described in GBD literature.^8^

### Mortality-to-incidence ratio

For select analyses in the Results section, we calculated the ratio of age-standardised mortality rates to age-standardised incidence rates of fires, heat and hot substances. These calculations were based on the same estimates reported in the Results section.

### Socio-demographic Index

SDI is an indicator that incorporates lag-distributed income per capita, mean educational attainment among people 15 years or older and total fertility rate under the age of 25. The SDI ranges from 0 to 1; 0 is assigned to the GBD location with the lowest income per capita, lowest educational attainment and highest fertility rate from 1980 to 2017, and 1 represents the level above which higher income per capita, higher educational attainment and lower fertility under 25 are no longer associated with health improvements across results from the GBD study. We show some results by SDI quintile in order to illustrate morbidity and mortality trends along different levels of development.

### Guidelines for Accurate and Transparent Health Estimates Reporting compliance

This study complies with the Guidelines for Accurate and Transparent Health Estimates Reporting recommendations (online supplementary appendix 2). All analytical code is available via healthdata.org.

10.1136/injuryprev-2019-043299.supp2 Supplementary data

## Results

Results were produced for 195 countries and territories in 1990 and 2017. Additional results with detail by age, sex, year and location are available via healthdata.org.

### Incidence

**Figure 1:**
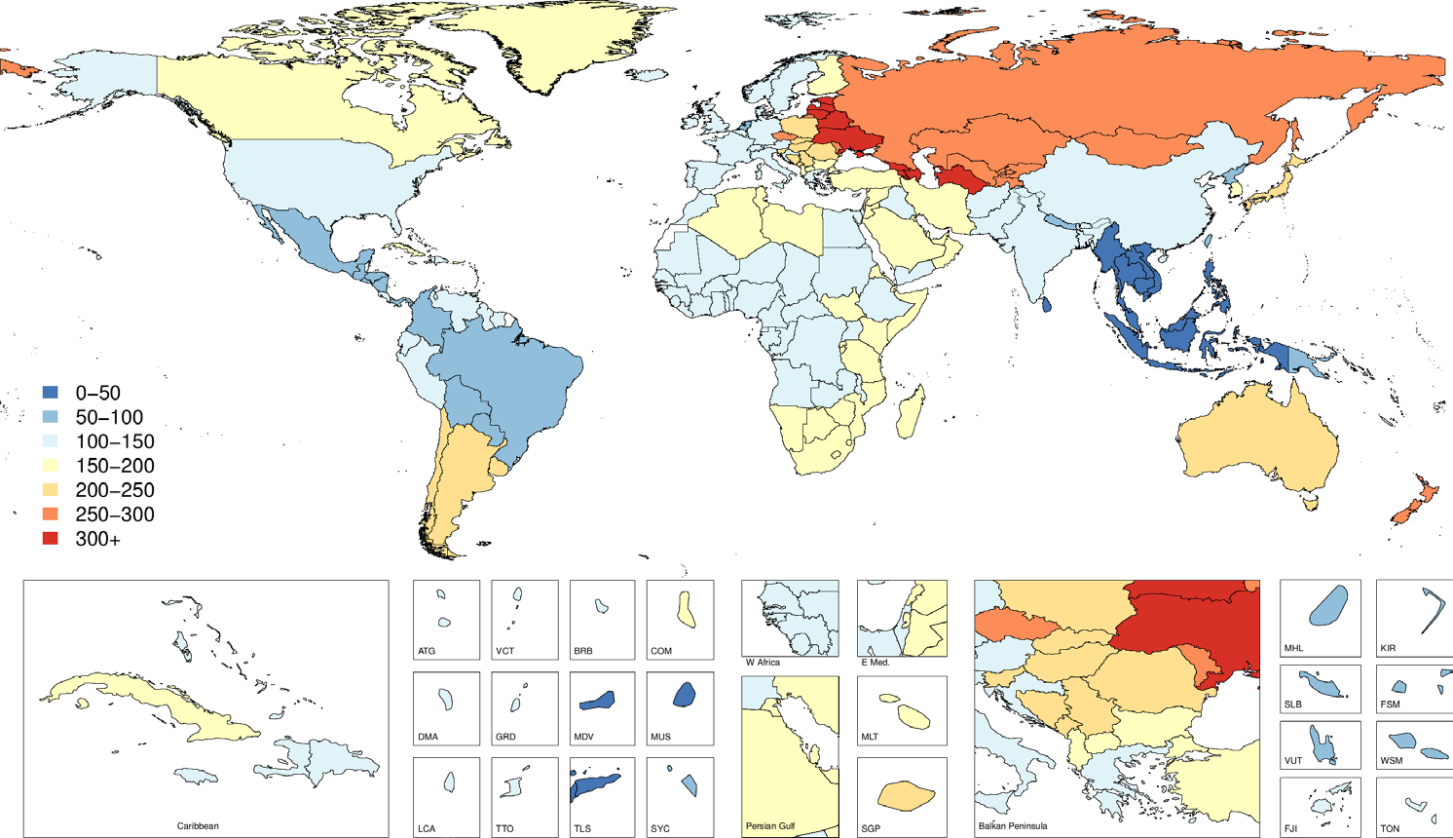
Map of age-standardised incidence per 100 000 of fire, heat and hot substance injuries by country and territory in 2017.

**Figure 2:**
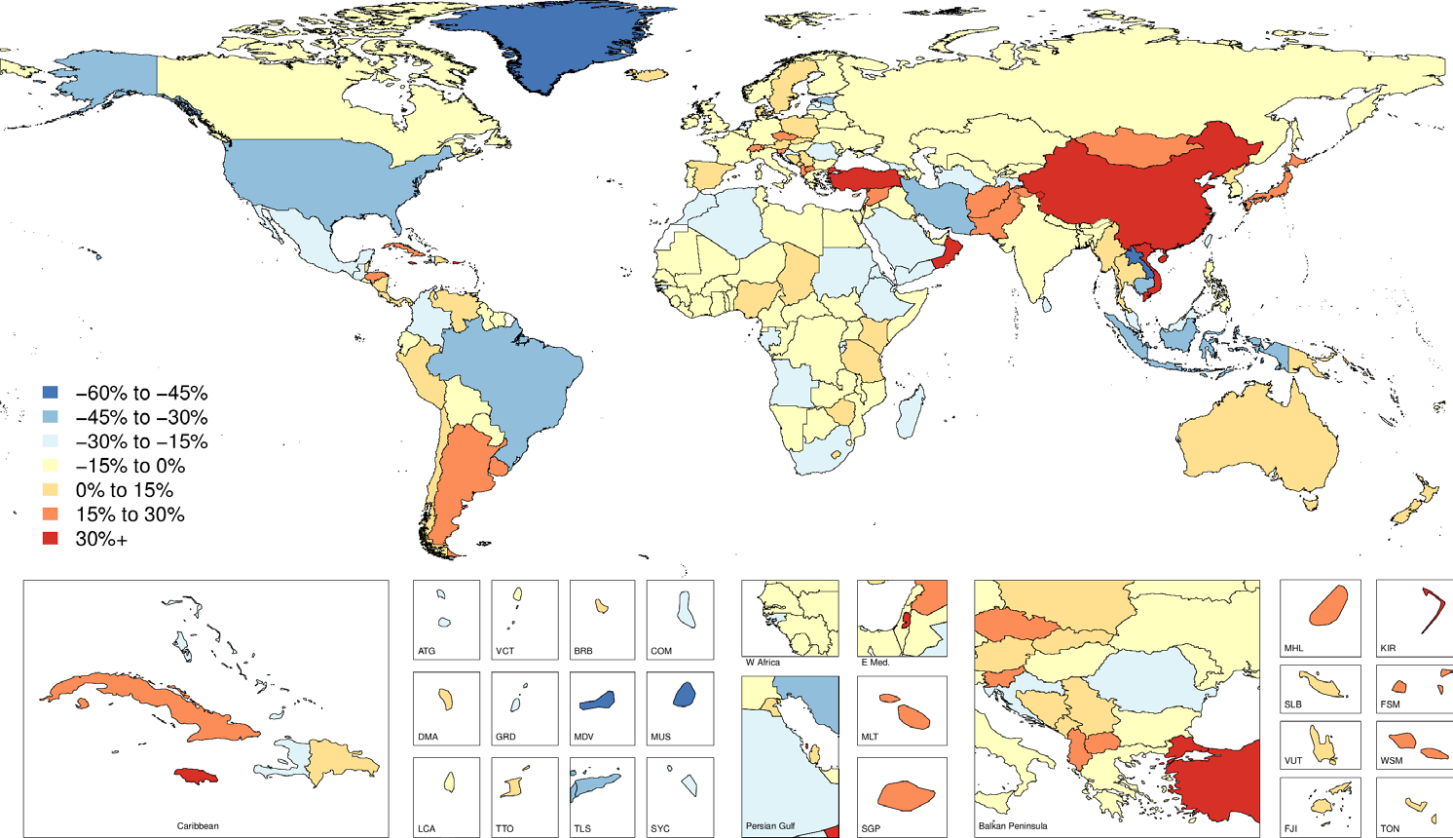
Map of percent change for age-standardised incidence per 100 000 of fire, heat and hot substance injuries by country and territory between 1990 and 2017.

Online supplementary appendix table 1 presents the all-age incidence counts and the age-standardised incidence rates for 2017 as well as the percentage change in age-standardised rates from 1990 to 2017. Figure 1 shows these geographical patterns by country with age-standardised incidence rates by country and territory for 2017. Figure 2 provides per cent change in age-standardised incidence rates by country and territory. Globally, the age-standardised incidence was 119 (95% uncertainty interval (UI) 99 to 142) per 100 000 in 2017, representing a non-significant decline of 5.4% (−11.1 to 0.3) from 1990 to 2017, and equating to 8 991 468 (7 481 218 to 10 740 897) cases in 2017. The age-standardised incidence rate did not experience significant change for any SDI quintiles except the high SDI quintile, where it decreased significantly, by 12.0% (−17.4 to −6.7). The regions with the highest age-standardised incidence rates in 2017 were Eastern Europe with 303 (256 to 359) cases per 100 000, Central Asia with 298 (251 to 353) cases per 100 000 and Southern Latin America with 226 (187 to 272) cases per 100 000. Among the 21 GBD regions, 9 experienced significant decreases in age-standardised incidence rates (Eastern Europe, Eastern sub-Saharan Africa, North Africa and Middle East, Central Asia, Central Latin America, Southeast Asia, Southern sub-Saharan Africa, Tropical Latin America, High-income North America), 3 regions experienced significant increases in age-standardised incidence rates (East Asia, Southern Latin America, High-income Asia Pacific) and the remaining 9 regions experienced no significant change in age-standardised incidence rates (Australasia, Oceania, Caribbean, South Asia, Central Europe, Western sub-Saharan Africa, Western Europe, Andean Latin America and Central sub-Saharan Africa).

10.1136/injuryprev-2019-043299.supp3 Supplementary data

### Prevalence

Online supplementary appendix table 1 shows the all-age prevalence counts and the age-standardised prevalence for 2017 as well as the percentage change in age-standardised prevalence from 1990 to 2017. Globally, the age-standardised prevalence was 1247 (95% UI 1065 to 1452) per 100 000 in 2017, representing a decline of 9.7% (−11.7 to −7.8) from 1990 to 2017, and equating to 99 746 814 (85 298 471 to 115 988 070) cases in 2017. East Asia had the highest number of prevalent cases in 2017 with 21 434 056 (17 943 830 to 25 366 091) cases across all ages and both sexes. The age-standardised prevalence rate decreased in every SDI quintile except the middle SDI quintile (where it did not change), with the largest decline in the high SDI quintile, at 11.4% (−13.0 to −10.0). The regions with the highest age-standardised prevalence rates were Central Asia with 2581 (2203 to 2981) cases per 100 000, Eastern Europe with 2346 (2006 to 2743) cases per 100 000 and High-income Asia Pacific with 2257 (1918 to 2658) cases per 100 000. Among the 21 GBD regions, 14 experienced significant decreases in age-standardised prevalence rates, 3 regions experienced significant increases in age-standardised prevalence (Australasia, High-income Asia Pacific, East Asia) and the remaining 4 regions experienced no significant change in age-standardised prevalence (Southern Latin America, Oceania, Caribbean, South Asia).

### Cause-specific mortality

**Figure 3:**
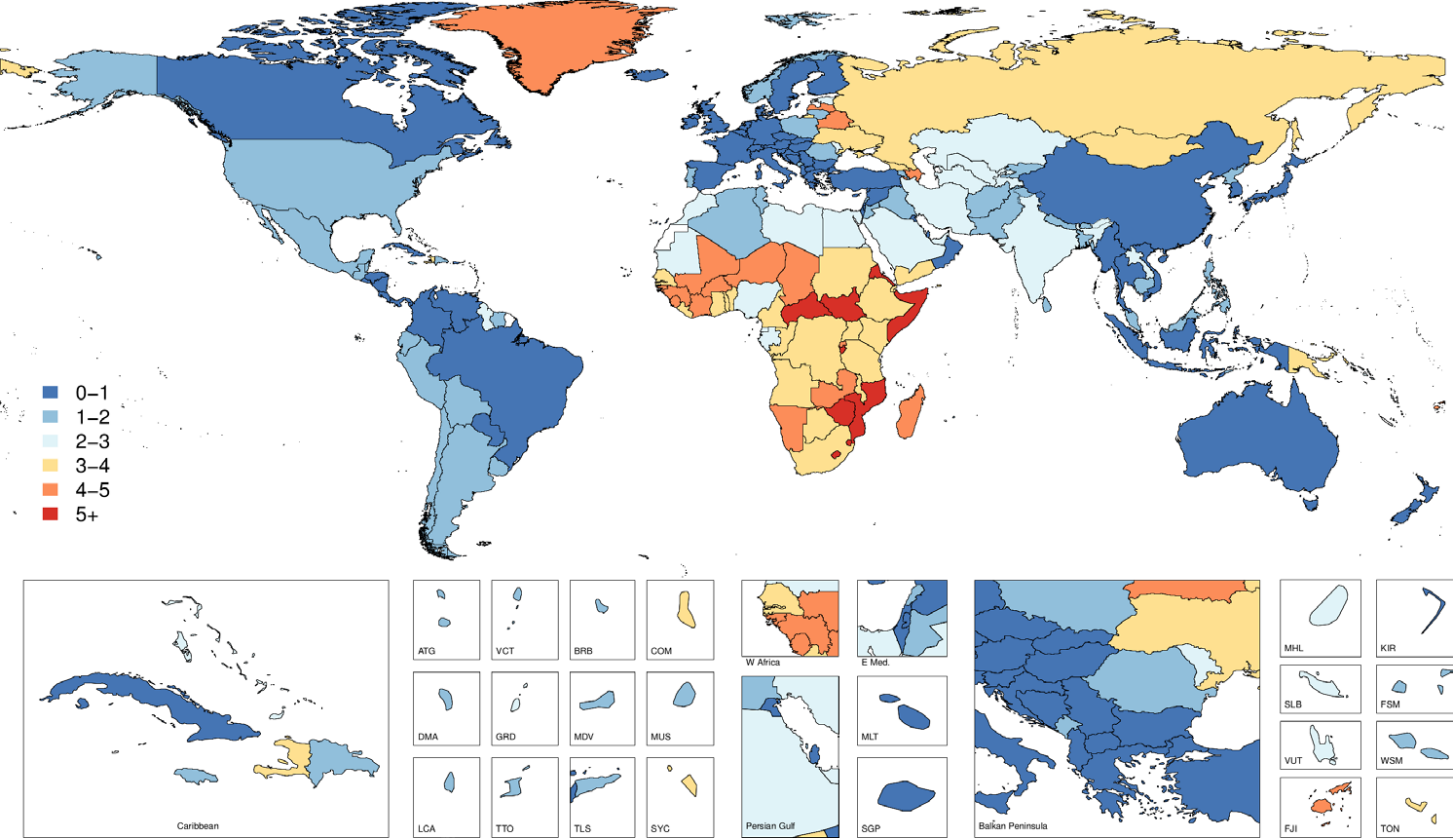
Map of age-standardised mortality per 100 000 of fire, heat and hot substance injuries by country and territory in 2017.

**Figure 4:**
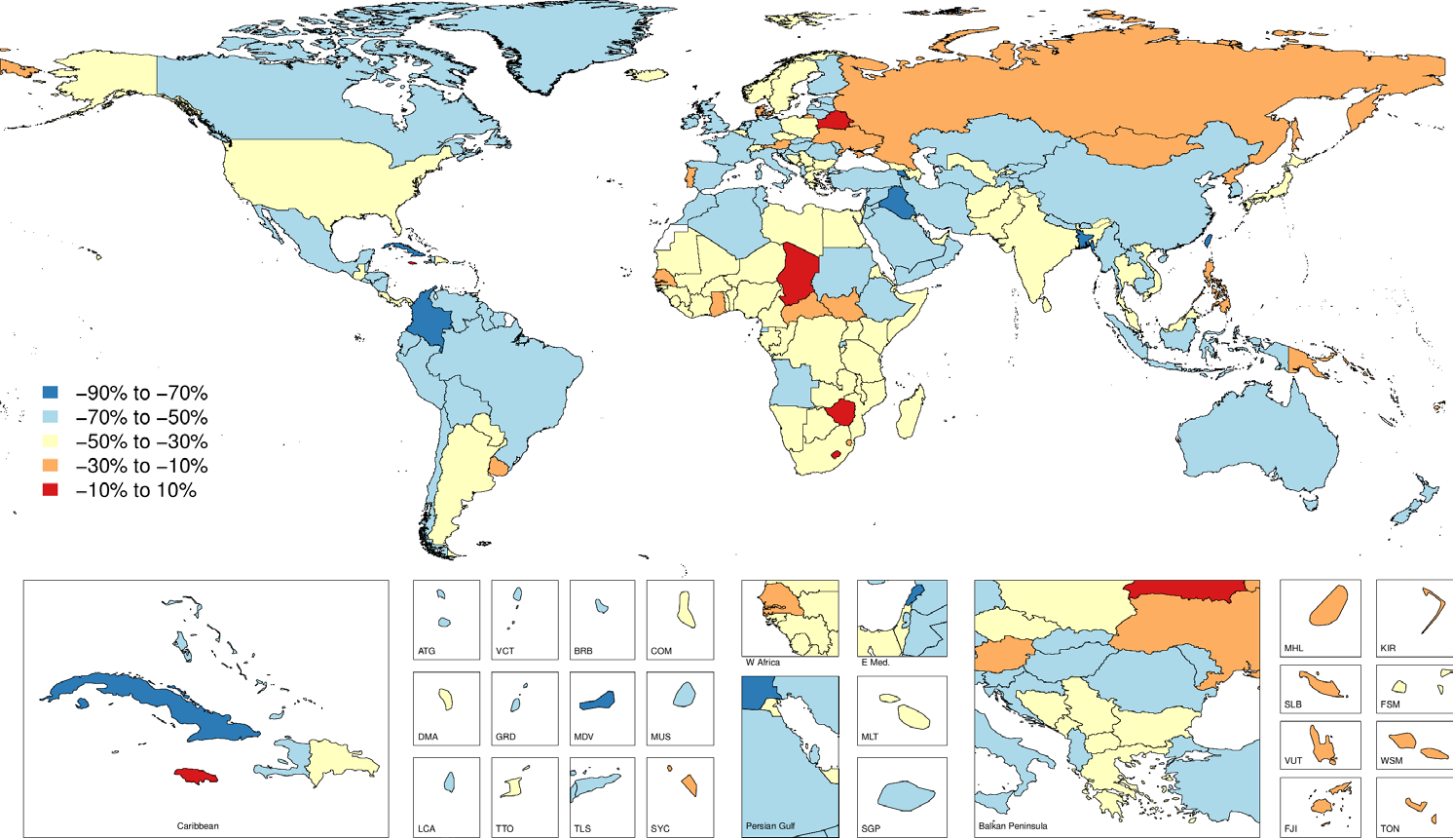
Map of percent change for age-standardised mortality of fire, heat and hot substance injuries by country and territory between 1990 and 2017.

Online supplementary appendix table 2 shows the all-age death counts and the age-standardised mortality rates for 2017 as well as the percentage change in age-standardised rates from 1990 to 2017. Figure 3 shows mortality rates by country and territory for 2017, while figure 4 shows changes in mortality rates by country and territory from 1990 to 2017. Globally, the age-standardised mortality rate was 1.6 (95% UI 1.3 to 1.7), which equated to 120 632 (101 630 to 129 383) deaths in 2017 and represented a 46.6% (−49.7 to −38.8) decrease in age-standardised mortality from 1990 to 2017. Every SDI quintile experienced a significant decrease in age-standardised mortality rates, with the greatest decline in the high SDI quintile, by 55.2% (−56.1 to −54.2) from 1990 to 2017. The regions with the highest age-standardised mortality rates were Southern sub-Saharan Africa with 4.3 (3.6 to 5.1) deaths per 100 000, Eastern sub-Saharan Africa with 4.0 (3.4 to 4.7) deaths per 100 000 and Central sub-Saharan Africa with 3.7 (2.4 to 4.7) deaths per 100 000. South Asia had the highest number of deaths, with 31 684 (23 098 to 36 828) deaths estimated in 2017.

10.1136/injuryprev-2019-043299.supp4 Supplementary data

### YLDs, YLLs and DALYs

Online supplementary appendix table 3 shows the counts, age-standardised rates and percentage change from 1990 to 2017 of YLDs, YLLs and DALYs. Globally, fire, heat and hot substances resulted in 5 286 270 (95% UI 4 308 890 to 5 836 389) YLLs, 3 177 003 (2 210 387 to 4 396 730) YLDs and 8 463 273 (7 034 048 to 9 881 099) DALYs, reflecting age-standardised rates of 71 (58 to 79) per 100 000, 40 (28 to 55) per 100 000 and 111 (93 to 129) per 100 000, respectively. Age-standardised YLLs, YLDs and DALYs declined by 50.8% (−55.8 to −39.3), 24.4% (−29.4 to −19.3) and 43.7% (−49.3 to −34.1), respectively, between 1990 and 2017. The region with the highest age-standardised DALY rate was Southern sub-Saharan Africa with 237 (198 to 281) DALYs per 100 000 which represented 171 (137 to 208) YLLs per 100 000 and 67 (48 to 88) YLDs per 100 000.

10.1136/injuryprev-2019-043299.supp5 Supplementary data

### Mortality-to-incidence ratios

**Figure 5:**
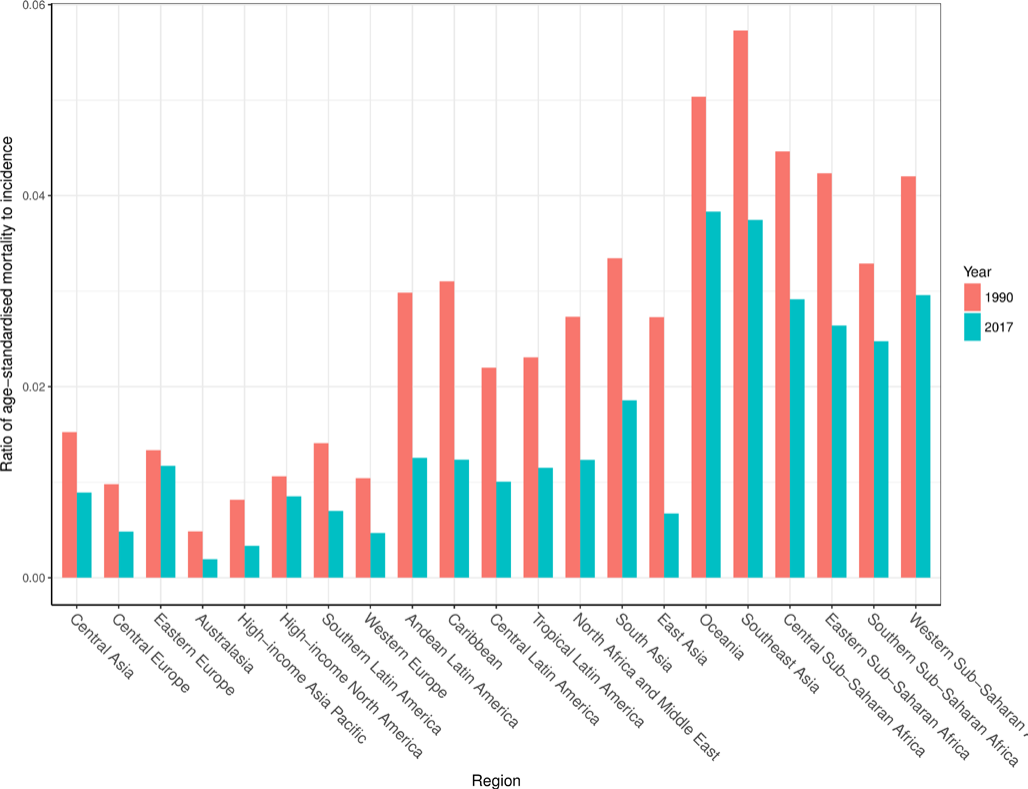
Mortality-to-incidence ratios for 1990 and 2017 by GBD region.

Figure 5 shows the ratios of age-standardised mortality rates to age-standardised incidence rates by region in 1990 and 2017, which approximates the risk of death given a burn injury. This figure shows how the mortality-to-incidence ratios (MIR) vary by both time and location. Oceania had the highest MIR in 2017, while Australasia had the lowest, following the patterns of percentage DALYs caused by YLDs as described above. While MIR varied substantially across regions, it also declined in every region from 1990 to 2017.

### Nature of injuries caused by fire, heat and hot substances

**Figure 6:**
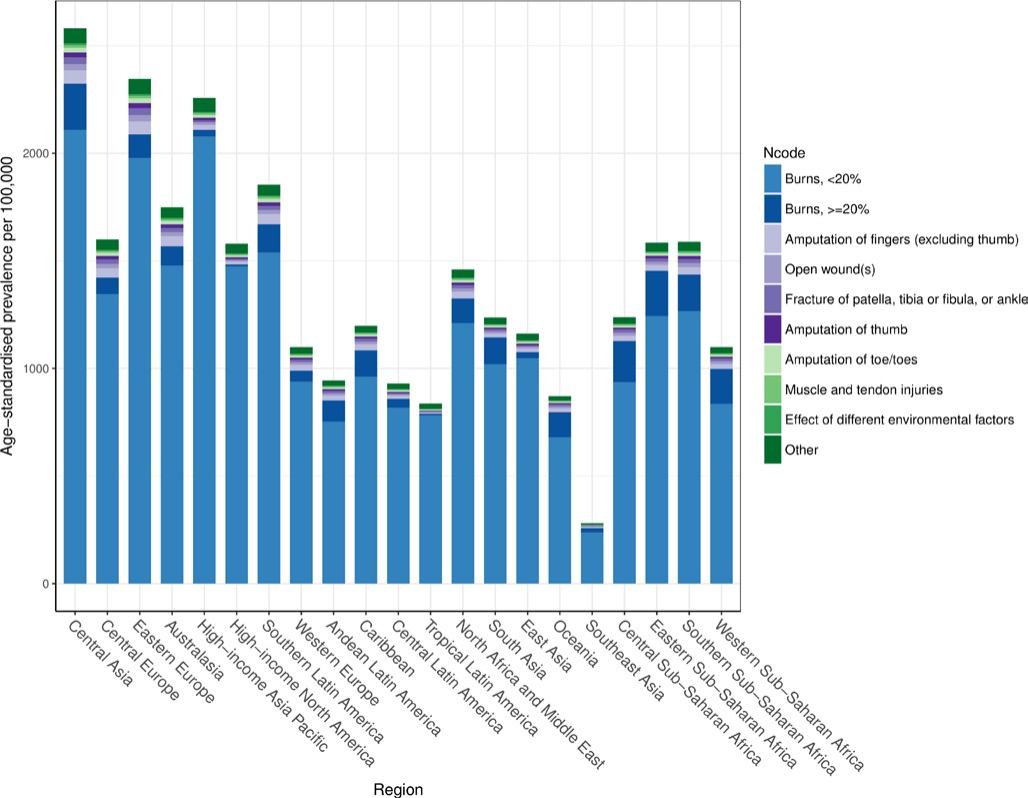
Composition of age-standardised prevalence by nature of injury category among all fire, heat and hot substance prevalent cases.

The average disability weight globally after comorbidity adjustment was 0.032, meaning that the average person suffering from a fire, heat and hot substance injury lost 3.2% of their full health status. This relatively small disability weight likely represents the large percentage of fire, heat and hot substance victims who experience less severe injuries. Figure 6 shows the distribution of nature of injury codes among the fire, heat and hot substance external cause codes for age-standardised prevalence by region. This figure shows that the leading cause of disability for victims of fire, heat and hot substances is—by far—burns affecting less than 20% surface area. Burns affecting over 20% of the body surface area are a relatively small proportion of all disability, as are the other nature of injury codes.

## Discussion

Our study is the first to report global, regional, national and SDI-based estimates for fire, heat and hot substances in terms of incidence, prevalence, mortality, YLDs, YLLs and DALYs with the GBD 2017 study framework and findings. These results are of great value to health professionals and policymakers who can reduce burden and set priorities to develop and implement curative and prevention programmes to reduce incidence and ensure proper recovery in the event of these injuries. Our results point to three central themes in the epidemiology of injuries due to fire, heat and hot substances.

The first theme pertains to the relationship between burden and geography, both in terms of level of development and region of the world. As mentioned in the Introduction section, other epidemiological studies have observed this relationship and have provided some perspective on the reasons for this relationship. Our study observed similar trends, with SDI quintile and geographical region largely impacting the burden in terms of incidence, mortality and DALYs, as evidenced in the results tables.

The second theme is an extension of this observation. Development and geography appear to govern the incidence and the mortality of fires, heat and hot substances. The relationship between geographical region and mortality becomes more pronounced in the results shown in figure 5, which suggests that while lower income areas are more susceptible to fire, heat and hot substances as a cause of injury, they also experience higher mortality rates when these injuries occur. In other words, region appears to affect the risk of these injuries and explains the higher fatal burden driven by these causes. The higher MIRs in these regions suggest that one of the factors driving the relationship between income and health loss from fire, heat and hot substances is a lack of access to treatment and care facilities. Indeed, the body of literature that has explored the relationship between rapid access and treatment to care in the event of fire-related injuries substantiates the likely reason for the MIR distributions observed in this study.^28^ It is possible that the assumed relationship between income and mortality as described in the modelling process is contributing to this effect, and future research should be focused on using more data sets that allow for computation of case fatality rates to provide more guidance for future modelling updates. Similarly, it would be valuable to measure the effects of smoke alarms and smoking regulations on the incidence of these injuries or other variables that may be more relevant in certain geographies such as the use of certain types of cooking fuel.

The third theme is that across global locations and income groups, the burden of these injuries in terms of fatal and non-fatal health outcomes generally decreased between 1990 and 2017. Nine of 21 GBD regions experienced a significantly decreased burden from fires, heat and hot substances. It is likely these trends are partially explained by improvements in income over the 28-year period of our study as well as general improvements in safety and healthcare access and quality and decreases in smoking in certain areas of the world. Exceptions to this trend in terms of age-standardised incidence included areas of Latin America and Asia with countries such as China, Vietnam and Argentina within these regions demonstrating an increase in age-standardised incidence over time. There are several possible reasons for these exceptions. It is possible that population expansion and urban crowding in densely populated locations have led to living and working conditions that increase the risk of fire-related injuries, particularly in areas where development growth has not been commensurate with population growth and density. These trends could lead to unsafe infrastructure and heightened risk of fire injures in concentrated population spaces. While establishing the underlying reason for the fire, heat or hot substance injury was outside the scope of this study. Future GBD studies could potentially explore these reasons in more detail, particularly as more clinical data with detailed ICD coding become available or as other literature and survey-based estimates are added to the GBD.

There are a number of avenues for future research. Research on the epidemiology of fire, heat and hot substances might further measure the decreased quality of life that results from a fire-related injury and that is not necessarily captured in the GBD disability weight measurement. Such research might focus specifically on the economic implications of injury. For example, some studies have attempted to quantify the lost human capital resulting from burn injuries. One study in Spain estimated the mean annual cost (direct and indirect) of burn-related fatalities and hospital admissions was US$99 773, which included the cost of temporary and permanent disability in addition to medical care. The study also noted that burns were the costliest condition in their analysis, outranking stroke and HIV/AIDS.^29^ In the USA, one study estimated that the cost in terms of human capital per medically treated burn injury amounted to US$15 733 (2017 US$) after accounting for acute and long-term treatment, wage loss following injury and work loss in the event of permanent disability.^30^ A report released by the English Office of the Deputy Prime Minister notes the ethical barriers to and difficulties of associating monetary values with a human’s worth but recognises the necessity of the practice as these monetary estimates play an important role in policy development in terms of measuring both the immediate and long-term consequences of a serious burn injury, including lifelong disability, loss of work for the injured and family caregivers, psychological suffering and medical costs.^31^

Our study had several limitations which should be considered in the interpretation and utilisation of these results. First, a general limitation in the GBD study is that some areas of the world where the risk of an injury caused by fire, heat and hot substances is thought to be high can at times have relatively low data coverage for high-quality vital registration mortality data as well as hospital and clinical data that are used to inform our non-fatal modelling process. In addition, some individuals suffering minor injuries from these causes may not reliably seek medical care. An ongoing objective of the GBD study is to continue adding high-quality cause of death as well as incidence and prevalence data to our database and modelling framework.

A second limitation pertains to the number and type of data sets that inform the relationship between the cause of injury and the injuries that result, such as burns, amputations and other bodily harm. These relationships likely vary by age, sex, location and year, and currently we do not have hospital or clinical data from many lower-income areas of the world where the incidence of these injuries appears to be highest. Therefore, it will be advantageous in future iterations of this study to continue adding clinical data that include both cause-of-injury ICD codes and nature of injury codes so these relationships can continue to be measured with improved accuracy, which could also lead to more detailed sequelae estimates including contractures. Current research and development with the Global Burn Registry Form may also lead to improved data collection methods for these injuries.^7 32 33^

An additional limitation pertains to the burden of self-inflicted burns in areas of South and Southeast Asia. Prior research has characterised the high number of deliberate burn cases in India, Sri Lanka, Nepal and other countries.^34–36^ While the external cause for these injuries should in theory be classified as self-harm or intentional injury in the GBD hierarchy, it is possible that at the hospital level, diagnosis classification avoids certain codes due to stigmatisation of self-harm. Additionally, this study focuses on the burden of fire, heat and hot substances and does not include analysis of burns that result from other causes, which is important as other research has identified burns caused by interpersonal violence and gender-based violence as critical issues in countries including India and Nepal.^37 38^ Future research should also consider burns specifically from causes such as self-immolation, which would inform more focused prevention and intervention strategies.^39^

A final limitation is that we currently do not estimate the underlying reason or circumstances for exposure to fire, heat and hot substances, for example, exposure from cooking fires versus occupational injuries in the workplace. The available literature suggests that these mechanisms vary by country, depending on income, cultural idiosyncrasies and other factors.^2 6 40 41^ For example, one study noted that burn incidents in North Karnataka, India, tended to be concentrated among females wearing synthetic clothing who were exposed to open flames in the kitchen.^42^ Identifying underlying reasons for a fire, heat, and hot substance injury could help inform policy and discourse focused on mitigating the specific risks of exposure to these injuries for given populations.

## Conclusion

The incidence and mortality due to fire, heat and hot substances are generally in decline, though there are important exceptions to these patterns among fatal and non-fatal health outcomes. This study includes burden estimates for every country and should be used to inform priorities and goals in health policy. The variation in these patterns highlights the need for universal access to care services that can mitigate disability and death from these injuries, as well as the importance of injury prevention methods that emphasise safety in consumer products and residential and occupational spaces as populations grow and concentrate as well as education and policy-level interventions. In addition, it will be important to continue collecting more detailed data on the underlying reasons for exposure to fire, heat and hot substances and to continue expanding the geographical coverage of data sources in epidemiological studies on the burden of these injuries.

**What is already known on the subject**

- Existing research suggests that exposure to fire, heat and hot substances causes significant morbidity and mortality globally, and that the burden of these injuries falls disproportionately in lower resource settings.

**What this study adds**

- This study provides further evidence that the burden of morbidity and mortality due to fire, heat and hot substances more heavily afflicts lower income locations.
- This research found that from 1990 to 2017, just three of 21 Global Burden of Disease regions experienced a significant increase in age-standardised incidence rates of injury caused by fire, heat and hot substances.
- This study measured wide variation in terms of trends over time across countries with some countries such as China increasing by more than 30% in terms of age-standardised incidence rates, while other countries such as Greenland decreased by more than 45%.

## References

[1] Capek KD, Sousse LE, Hundeshagen G, et al Contemporary burn survival. J Am Coll Surg 2018;226:453–63. 10.1016/j.jamcollsurg.2017.12.045 29530306 PMC6027619

[2] Peck M, Pressman MA The correlation between burn mortality rates from fire and flame and economic status of countries. Burns 2013;39:1054–9. 10.1016/j.burns.2013.04.010 23768720

[3] Smolle C, Cambiaso-Daniel J, Forbes AA, et al Recent trends in burn epidemiology worldwide: a systematic review. Burns 2017;43:249–57. 10.1016/j.burns.2016.08.013 27600982 PMC5616188

[4] Abouie A, Salamati P, Hafezi-Nejad N, et al Incidence and cost of non-fatal burns in Iran: a nationwide population-based study. Int J Inj Contr Saf Promot 2018;25:23–30. 10.1080/17457300.2017.1310739 28387170

[5] Sadeghian F, Saeedi Moghaddam S, Saadat S, et al The trend of burn mortality in Iran - A study of fire, heat and hot substance-related fatal injuries from 1990 to 2015. Burns 2019;45:228–40. 10.1016/j.burns.2018.09.006 30274812

[6] Forjuoh SN Burns in low- and middle-income countries: a review of available literature on descriptive epidemiology, risk factors, treatment, and prevention. Burns 2006;32:529–37. 10.1016/j.burns.2006.04.002 16777340

[7] Hyder AA, Sugerman DE, Puvanachandra P, et al Global childhood unintentional injury surveillance in four cities in developing countries: a pilot study. Bull World Health Organ 2009;87:345–52. 10.2471/BLT.08.055798 19551252 PMC2678776

[8] James SL, Abate D, Abate KH, et al Global, regional, and national incidence, prevalence, and years lived with disability for 354 diseases and injuries for 195 countries and territories, 1990–2017: a systematic analysis for the global burden of disease study 2017. The Lancet 2018;392:1789–858. 10.1016/S0140-6736(18)32279-7 PMC6227754 30496104

[9] Dicker D, Nguyen G, Abate D, et al Global, regional, and national age-sex-specific mortality and life expectancy, 1950–2017: a systematic analysis for the global burden of disease study 2017. The Lancet 2018;392:1684–735. 10.1016/S0140-6736(18)31891-9 PMC6227504 30496102

[10] Kyu HH, Abate D, Abate KH, et al Global, regional, and national disability-adjusted life-years (DALYs) for 359 diseases and injuries and healthy life expectancy (HALE) for 195 countries and territories, 1990–2017: a systematic analysis for the global burden of disease study 2017. The Lancet 2018;392:1859–922. 10.1016/S0140-6736(18)32335-3 PMC6252083 30415748

[11] Murray CJL, Callender CSKH, Kulikoff XR, et al Population and fertility by age and sex for 195 countries and territories, 1950–2017: a systematic analysis for the global burden of disease study 2017. The Lancet 2018;392:1995–2051. 10.1016/S0140-6736(18)32278-5 PMC6227915 30496106

[12] Roth GA, Abate D, Abate KH, et al Global, regional, and national age-sex-specific mortality for 282 causes of death in 195 countries and territories, 1980–2017: a systematic analysis for the global burden of disease study 2017. The Lancet 2018;392:1736–88. 10.1016/S0140-6736(18)32203-7 PMC6227606 30496103

[13] Stanaway JD, Afshin A, Gakidou E, et al Global, regional, and national comparative risk assessment of 84 behavioural, environmental and occupational, and metabolic risks or clusters of risks for 195 countries and territories, 1990–2017: a systematic analysis for the global burden of disease study 2017. The Lancet 2018;392:1923–94. 10.1016/S0140-6736(18)32225-6 PMC6227755 30496105

[14] Naghavi M, Abajobir AA, Abbafati C, et al Global, regional, and national age-sex specific mortality for 264 causes of death, 1980–2016: a systematic analysis for the global burden of disease study 2016. The Lancet 2017;390:1151–210. 10.1016/S0140-6736(17)32152-9 PMC5605883 28919116

[15] Foreman KJ, Naghavi M, Ezzati M Improving the usefulness of US mortality data: new methods for reclassification of underlying cause of death. Popul Health Metr 2016;14:14 10.1186/s12963-016-0082-4 27127419 PMC4848792

[16] Naghavi M, Makela S, Foreman K, et al Algorithms for enhancing public health utility of national causes-of-death data. Popul Health Metr 2010;8:9 10.1186/1478-7954-8-9 20459720 PMC2873308

[17] Foreman KJ, Lozano R, Lopez AD, et al Modeling causes of death: an integrated approach using CODEm. Popul Health Metr 2012;10:1 10.1186/1478-7954-10-1 22226226 PMC3315398

[18] Fullman N, Yearwood J, Abay SM, et al Measuring performance on the healthcare access and quality index for 195 countries and territories and selected subnational locations: a systematic analysis from the global burden of disease study 2016. Lancet 2018;391:2236–71. 10.1016/S0140-6736(18)30994-2 29893224 PMC5986687

[19] Vos T, Abajobir AA, Abate KH, et al Global, regional, and national incidence, prevalence, and years lived with disability for 328 diseases and injuries for 195 countries, 1990–2016: a systematic analysis for the global burden of disease study 2016. The Lancet 2017;390:1211–59. 10.1016/S0140-6736(17)32154-2 PMC5605509 28919117

[20] Mackenzie EJ, Rivara FP, Jurkovich GJ, et al The national study on costs and outcomes of trauma. J Trauma 2007;63(6 Suppl):S54–S67. 10.1097/TA.0b013e31815acb09 18091213

[21] FederalRegister Traumatic Brain Injury(TBI) Follow-Up Registry and Surveillance of TBI in the Emergency Department (ED); Notice of Availability of Funds. Available: https://www.federalregister.gov/documents/2002/05/08/02-11359/traumatic-brain-injurytbi-follow-up-registry-and-surveillance-of-tbi-in-the-emergency-department-ed [Accessed 14 May 2018].

[22] GHDx China Zhuhai Study 2006-2007 - China CDC. Available: http://ghdx.healthdata.org/record/china-zhuhai-study-2006-2007-china-cdc [Accessed 15 May 2018].

[23] GHDx Functional outcome at 2.5, 5, 9, and 24 months after injury in the Netherlands. Available: http://ghdx.healthdata.org/record/functional-outcome-25-5-9-and-24-months-after-injury-netherlands [Accessed 15 May 2018]. 10.1097/TA.0b013e31802b71c9 17215744

[24] GHDx Health-Related quality of life after burns: a prospective multicentre cohort study with 18 months follow-up. Available: http://ghdx.healthdata.org/record/health-related-quality-life-after-burns-prospective-multicentre-cohort-study-18-months-follow [Accessed 15 May 2018]. 10.1097/ta.0b013e3182199072 22439227

[25] GHDx Netherlands injury surveillance system 2007. Available: http://ghdx.healthdata.org/record/netherlands-injury-surveillance-system-2007 [Accessed 15 May 2018].

[26] GHDx Netherlands injury surveillance system 2010. Available: http://ghdx.healthdata.org/record/netherlands-injury-surveillance-system-2010 [Accessed 14 May 2018].

[27] GHDx China National Injury Surveillance System 2006 - China CDC. Available: http://ghdx.healthdata.org/record/china-national-injury-surveillance-system-2006-china-cdc [Accessed 1 Jul 2018].

[28] Wood F Burn care: the challenges of research. Burns Trauma 2013;1 10.4103/2321-3868.123071 PMC4978089 27574632

[29] Sanchez JLA, Bastida JL, Martínez MM, et al Socio-Economic cost and health-related quality of life of burn victims in Spain. Burns 2008;34:975–81. 10.1016/j.burns.2007.12.011 18472221

[30] Seifert J Incidence and economic burden of injuries in the United States. J Epidemiol Community Health 2007;61 10.1136/jech.2007.059717

[31] The economic cost of fire: estimates for 2004, 2006 Available: http://webarchive.nationalarchives.gov.uk/20120919224256/http://www.communities.gov.uk/documents/fire/pdf/144524.pdf [Accessed 29 Jun 2018].

[32] Ivers RQ, Hunter K, Clapham K, et al Understanding burn injuries in Aboriginal and Torres Strait Islander children: protocol for a prospective cohort study. BMJ Open 2015;5:e009826 10.1136/bmjopen-2015-009826 PMC4606434 26463225

[33] WHO Global burn registry. who. Available: http://www.who.int/violence_injury_prevention/burns/gbr/en/ [Accessed 7 May 2019].

[34] Laloë V Patterns of deliberate self-burning in various parts of the world. A review. Burns 2004;30:207–15. 10.1016/j.burns.2003.10.018 15082345

[35] Mohanty MK, Arun M, Monteiro FNP, et al Self-Inflicted burns fatalities in Manipal, India. Med Sci Law 2005;45:27–30. 10.1258/rsmmsl.45.1.27 15745270

[36] Laloë V Epidemiology and mortality of burns in a general Hospital of eastern Sri Lanka. Burns 2002;28:778–81. 10.1016/S0305-4179(02)00202-4 12464477

[37] Natarajan M Differences between intentional and non-intentional burns in India: implications for prevention. Burns 2014;40:1033–9. 10.1016/j.burns.2013.12.002 24433938

[38] Lama BB, Duke JM, Sharma NP, et al Intentional burns in Nepal: a comparative study. Burns 2015;41:1306–14. 10.1016/j.burns.2015.01.006 25716765

[39] Rezaeian M The geographical belt of self-immolation. Burns 2017;43:896–7. 10.1016/j.burns.2017.01.001 29032971

[40] Peck MD Epidemiology of burns throughout the world. Part I: distribution and risk factors. Burns 2011;37:1087–100. 10.1016/j.burns.2011.06.005 21802856

[41] Peck MD, Kruger GE, van der Merwe AE, et al Burns and fires from non-electric domestic appliances in low and middle income countries Part I. The scope of the problem. Burns 2008;34:303–11. 10.1016/j.burns.2007.08.014 18206314

[42] Shankar G, Naik VA, Powar R Epidemiolgical study of burn injuries admitted in two hospitals of North Karnataka. Indian J Community Med 2010;35:509–12. 10.4103/0970-0218.74363 21278873 PMC3026131

