# Supplementary material for: Epidemiology of injuries from fire, heat and hot substances: global, regional and national morbidity and mortality estimates from the Global Burden of Disease 2017 study
Source: Inj Prev. 2019 Dec 18;26(Suppl 1):i36–45. doi: 10.1136/injuryprev-2019-043299 (PMC7571358; doi:10.1136/injuryprev-2019-043299)
Supplement: Supplementary data [file injuryprev-2019-043299supp002.pdf]

Appendix 2

GATHER checklist of information that should be included in reports of global health estimates, with description of compliance and location of information for GBD 2017.

#	GATHER checklist item	Description of compliance	Reference
**Objectives and funding**			
1	Define the indicators, populations, and time periods for which estimates were made.	Narrative provided in paper and appendix describing indicators, definitions, and populations	Main text (Methods) and appendix
2	List the funding sources for the work.	Funding sources listed in paper	Summary (Funding)
**Data Inputs**			
For all data inputs from multiple sources that are synthesised as part of the study:			
3	Describe how the data were identified and how the data were accessed.	Narrative description of data seeking methods provided	Main text (Methods) and appendix
4	Specify the inclusion and exclusion criteria. Identify all ad-hoc exclusions.	Narrative about inclusion and exclusion criteria by data type provided; ad hoc exclusions in cause-specific write-ups	Main text (Methods) and appendix
5	Provide information on all included data sources and their main characteristics. For each data source used, report reference information or contact name/institution, population represented, data collection method, year(s) of data collection, sex and age range, diagnostic criteria or measurement method, and sample size, as relevant.	An interactive, online data source tool that provides metadata for data sources by component, geography, cause, risk, or impairment has been developed	Online data citation tools: http://ghdx.healthdata.org/gbd-2017
6	Identify and describe any categories of input data that have potentially important biases (e.g., based on characteristics listed in item 5).	Summary of known biases by cause included in appendix	Appendix
For data inputs that contribute to the analysis but were not synthesised as part of the study:			
7	Describe and give sources for any other data inputs.	Included in online data source tool	http://ghdx.healthdata.org/gbd-2017
For all data inputs:			
8	Provide all data inputs in a file format from which data can be efficiently extracted (e.g., a spreadsheet as opposed to a PDF), including all relevant meta-data listed in item 5. For any data inputs that cannot be shared due to ethical or legal reasons, such as third-party ownership, provide a contact name or the name of the institution that retains the right to the data.	Downloads of input data available through online tools, including data visualisation tools and data query tools; input data not available in tools will be made available upon request	Online data visualisation tools, data query tools, and the Global Health Data Exchange
**Data analysis**			

9	Provide a conceptual overview of the data analysis method. A diagram may be helpful.	Flow diagrams of the overall methodological processes, as well as cause-specific modelling processes, have been provided	Main text (Methods) and appendix
10	Provide a detailed description of all steps of the analysis, including mathematical formulae. This description should cover, as relevant, data cleaning, data pre-processing, data adjustments and weighting of data sources, and mathematical or statistical model(s).	Flow diagrams and corresponding methodological write-ups for each cause, as well as the databases and modelling processes, have been provided	Main text (Methods) and appendix
11	Describe how candidate models were evaluated and how the final model(s) were selected.	Provided in the methodological write-ups	Appendix
12	Provide the results of an evaluation of model performance, if done, as well as the results of any relevant sensitivity analysis.	Provided in the methodological write-ups	Appendix
13	Describe methods for calculating uncertainty of the estimates. State which sources of uncertainty were, and were not, accounted for in the uncertainty analysis.	Appendix	Appendix
14	State how analytic or statistical source code used to generate estimates can be accessed.	Appendix	http://ghdx.healthdata.org/gbd-2017/code
**Results and Discussion**			
15	Provide published estimates in a file format from which data can be efficiently extracted.	GBD 2017 results are available through online data visualisation tools, the Global Health Data Exchange, and the online data query tool	Main text, and online data tools (data visualisation tools, data query tools, and the Global Health Data Exchange)
16	Report a quantitative measure of the uncertainty of the estimates (e.g. uncertainty intervals).	Uncertainty intervals are provided with all results	Main text, appendix, and online data tools (data visualisation tools, data query tools, and the Global Health Data Exchange)
17	Interpret results in light of existing evidence. If updating a previous set of estimates, describe the reasons for changes in estimates.	Discussion of methodological changes between GBD rounds provided in the narrative of the manuscript and appendix	Main text (Methods and Discussion) and appendix
18	Discuss limitations of the estimates. Include a discussion of any modelling assumptions or data limitations that affect interpretation of the estimates.	Discussion of limitations provided in the narrative of the main paper, as well as in the methodological write-ups in the appendix	Main text (Limitations) and appendix
