# Supplementary material for: Epidemiology of injuries from fire, heat and hot substances: global, regional and national morbidity and mortality estimates from the Global Burden of Disease 2017 study
Source: Inj Prev. 2019 Dec 18;26(Suppl 1):i36–45. doi: 10.1136/injuryprev-2019-043299 (PMC7571358; doi:10.1136/injuryprev-2019-043299)
Supplement: Supplementary data [file injuryprev-2019-043299supp003.pdf]

Table 1: Incidence and prevalence for 2017 and percentage change of age-standardised rates between 1990 and 2017 by location for fire, heat, and hot substances

Location	Incidence (95% UI)			Prevalence (95% UI)		
	2017 counts	2017 age-standardised rates per 100,000	Percentage change in age-standardised rates between 1990 and 2017	2017 counts	2017 age-standardised rates per 100,000	Percentage change in age-standardised rates between 1990 and 2017
**Global**	**8 991 468 (7 481 218 to 10 740 897)**	**119 (99 to 142)**	**-5.4 (-11.1 to 0.3)**	**99 746 814 (85 298 471 to 115 988 070)**	**1 247 (1 065 to 1 452)**	**-9.7 (-11.7 to -7.8)**
**Low SDI**	**1 614 670 (1 317 645 to 1 989 642)**	**120 (100 to 144)**	**-6.6 (-13.7 to 0.1)**	**12 270 568 (10 587 721 to 14 153 833)**	**1 213 (1 049 to 1 398)**	**-8.8 (-11.0 to -6.4)**
**Low-middle SDI**	**1 981 535 (1 635 732 to 2 415 489)**	**111 (93 to 134)**	**-0.1 (-6.6 to 6.3)**	**17 080 559 (14 654 098 to 19 754 153)**	**1 126 (970 to 1 299)**	**-6.0 (-8.3 to -4.0)**
**Middle SDI**	**1 806 274 (1 484 524 to 2 163 891)**	**89 (73 to 107)**	**3.7 (-2.5 to 10.2)**	**22 154 701 (18 718 049 to 25 997 440)**	**974 (822 to 1 147)**	**-2.8 (-5.8 to 0.2)**
**High-middle SDI**	**2 090 241 (1 761 446 to 2 455 491)**	**160 (133 to 190)**	**-3.6 (-9.9 to 2.5)**	**24 551 278 (20 806 660 to 28 686 636)**	**1 467 (1 241 to 1 722)**	**-8.5 (-11.4 to -5.4)**
**High SDI**	**1 471 747 (1 230 327 to 1 723 680)**	**153 (126 to 183)**	**-12.0 (-17.4 to -6.7)**	**23 299 055 (19 856 719 to 27 161 366)**	**1 567 (1 332 to 1 828)**	**-11.4 (-13.0 to -10.0)**
**Central Europe, Eastern Europe, and Central Asia**	**1 132 860 (970 064 to 1 313 154)**	**279 (236 to 331)**	**-6.6 (-12.3 to -1.3)**	**11 325 202 (9 705 049 to 13 148 167)**	**2 184 (1 857 to 2 536)**	**-9.7 (-12.1 to -7.0)**
**Central Asia**	**275 531 (232 548 to 326 341)**	**298 (251 to 353)**	**-13.0 (-20.8 to -5.5)**	**2 296 262 (1 956 542 to 2 656 620)**	**2 581 (2 203 to 2 981)**	**-16.3 (-19.0 to -13.3)**
Armenia	8 775 (7 326 to 10 427)	308 (255 to 372)	-14.8 (-24.1 to -4.4)	94 338 (79 584 to 111 039)	2 619 (2 203 to 3 085)	-17.4 (-21.8 to -12.7)
Azerbaijan	36 095 (31 023 to 42 108)	349 (297 to 407)	-12.9 (-21.0 to -4.9)	318 895 (273 157 to 366 653)	2 938 (2 521 to 3 382)	-20.9 (-24.9 to -17.2)
Georgia	11 297 (9 804 to 12 907)	322 (276 to 376)	-17.5 (-24.2 to -10.5)	120 981 (103 952 to 138 795)	2 622 (2 246 to 3 027)	-19.7 (-22.3 to -16.8)
Kazakhstan	53 228 (45 070 to 62 711)	298 (251 to 357)	-14.7 (-24.0 to -5.9)	456 271 (386 689 to 533 805)	2 481 (2 104 to 2 900)	-17.1 (-21.2 to -12.4)
Kyrgyzstan	18 071 (15 004 to 21 927)	274 (229 to 330)	-6.2 (-15.1 to 2.7)	141 743 (120 056 to 164 510)	2 457 (2 091 to 2 847)	-4.8 (-9.0 to -0.5)
Mongolia	9 830 (8 330 to 11 606)	293 (248 to 349)	22.3 (15.0 to 29.9)	85 221 (73 177 to 98 424)	2 705 (2 320 to 3 120)	13.6 (9.9 to 17.3)
Tajikistan	27 576 (22 977 to 33 240)	285 (240 to 339)	-19.6 (-26.8 to -12.0)	204 826 (174 496 to 237 236)	2 640 (2 263 to 3 031)	-21.8 (-25.1 to -18.1)
Turkmenistan	15 523 (13 122 to 18 369)	304 (257 to 363)	-25.9 (-33.1 to -18.0)	129 351 (110 038 to 150 863)	2 690 (2 293 to 3 125)	-31.9 (-34.8 to -28.6)
Uzbekistan	95 136 (79 413 to 113 872)	284 (238 to 340)	-6.2 (-15.3 to 2.4)	744 634 (632 698 to 864 656)	2 464 (2 100 to 2 849)	-11.3 (-15.4 to -7.3)
**Central Europe**	**220 207 (187 759 to 253 843)**	**208 (172 to 248)**	**-1.8 (-7.9 to 4.0)**	**2 434 812 (2 064 530 to 2 841 238)**	**1 599 (1 337 to 1 899)**	**-8.1 (-10.5 to -5.8)**
Albania	5 171 (4 297 to 6 063)	198 (163 to 236)	18.1 (10.8 to 26.1)	52 155 (43 765 to 61 113)	1 544 (1 285 to 1 841)	2.7 (-2.3 to 6.6)
Bosnia and Herzegovina	6 373 (5 314 to 7 432)	205 (168 to 249)	14.5 (7.4 to 21.6)	72 307 (61 517 to 84 647)	1 619 (1 357 to 1 925)	1.7 (-1.6 to 5.5)
Bulgaria	12 645 (10 617 to 14 618)	198 (163 to 236)	-2.9 (-11.3 to 5.2)	150 059 (127 733 to 175 238)	1 532 (1 285 to 1 817)	-9.3 (-14.6 to -4.9)
Croatia	5 481 (4 799 to 6 230)	135 (117 to 158)	-18.3 (-25.0 to -11.4)	58 814 (50 210 to 68 443)	1 004 (852 to 1 178)	-20.6 (-24.6 to -17.7)
Czech Republic	23 596 (20 028 to 27 234)	250 (208 to 298)	23.6 (16.1 to 31.7)	272 379 (231 065 to 319 835)	1 946 (1 634 to 2 337)	22.3 (18.1 to 28.7)
Hungary	17 666 (14 832 to 20 657)	203 (168 to 248)	-5.5 (-14.7 to 2.6)	205 569 (173 953 to 239 758)	1 574 (1 313 to 1 869)	-5.8 (-9.4 to -2.0)
Macedonia	3 993 (3 311 to 4 685)	197 (162 to 239)	21.4 (14.4 to 29.1)	42 337 (35 745 to 49 527)	1 543 (1 289 to 1 828)	8.2 (2.9 to 11.9)
Montenegro	1 196 (1 007 to 1 400)	203 (167 to 246)	5.0 (-2.0 to 13.6)	12 308 (10 354 to 14 398)	1 576 (1 312 to 1 860)	1.4 (-2.5 to 5.3)
Poland	75 772 (64 684 to 87 828)	211 (175 to 254)	3.1 (-4.1 to 10.9)	822 569 (693 934 to 962 791)	1 621 (1 352 to 1 924)	-4.0 (-7.8 to 0.5)
Romania	37 774 (32 424 to 43 592)	205 (171 to 242)	-25.2 (-32.6 to -17.6)	410 483 (348 027 to 476 278)	1 568 (1 313 to 1 850)	-32.1 (-35.5 to -28.7)
Serbia	16 313 (13 616 to 19 137)	200 (165 to 241)	8.8 (0.3 to 18.1)	177 902 (149 642 to 207 283)	1 557 (1 294 to 1 844)	1.7 (-2.1 to 5.8)
Slovakia	9 999 (8 359 to 11 700)	201 (165 to 241)	7.3 (0.6 to 14.5)	109 359 (92 397 to 128 132)	1 560 (1 306 to 1 852)	4.7 (1.6 to 9.4)
Slovenia	4 227 (3 575 to 4 817)	225 (190 to 265)	19.5 (11.8 to 27.5)	48 571 (41 272 to 57 013)	1 727 (1 440 to 2 072)	18.8 (14.0 to 23.7)
**Eastern Europe**	**637 123 (543 015 to 738 624)**	**303 (256 to 359)**	**-8.0 (-13.4 to -2.5)**	**6 594 128 (5 615 482 to 7 709 342)**	**2 346 (2 006 to 2 743)**	**-10.2 (-13.2 to -7.1)**
Belarus	33 658 (29 315 to 38 363)	346 (299 to 402)	-4.3 (-11.6 to 4.1)	330 068 (280 806 to 382 585)	2 556 (2 189 to 2 970)	-9.4 (-14.0 to -5.6)
Estonia	4 192 (3 636 to 4 824)	327 (278 to 385)	-33.7 (-40.2 to -26.5)	45 353 (38 625 to 52 681)	2 480 (2 113 to 2 889)	-32.4 (-36.0 to -28.3)
Latvia	6 532 (5 653 to 7 497)	339 (290 to 397)	-27.0 (-33.7 to -19.2)	70 472 (60 417 to 82 268)	2 531 (2 167 to 2 949)	-27.7 (-31.7 to -23.7)
Lithuania	8 071 (6 931 to 9 283)	305 (258 to 363)	-5.5 (-14.3 to 4.4)	94 576 (80 799 to 110 234)	2 392 (2 031 to 2 802)	-3.3 (-7.5 to 1.2)
Moldova	10 236 (8 797 to 11 944)	280 (237 to 334)	-10.0 (-18.5 to -1.7)	110 185 (94 307 to 127 755)	2 293 (1 953 to 2 656)	-11.6 (-16.1 to -6.7)
Russian Federation	437 326 (371 488 to 508 232)	299 (252 to 355)	-8.8 (-14.4 to -3.2)	4 482 636 (3 814 918 to 5 250 886)	2 318 (1 973 to 2 715)	-11.0 (-13.9 to -8.1)
Ukraine	137 108 (116 205 to 159 814)	309 (261 to 366)	-2.9 (-8.9 to 4.3)	1 460 839 (1 236 321 to 1 705 616)	2 382 (2 022 to 2 782)	-5.5 (-8.8 to 0.0)
**High-income**	**1 351 422 (1 133 053 to 1 588 240)**	**151 (125 to 182)**	**-13.4 (-18.6 to -8.1)**	**21 070 756 (17 986 161 to 24 493 202)**	**1 528 (1 298 to 1 785)**	**-14.4 (-16.2 to -12.8)**
**Australasia**	**52 135 (43 400 to 61 911)**	**219 (180 to 267)**	**5.3 (-0.7 to 11.8)**	**585 729 (495 935 to 704 719)**	**1 749 (1 475 to 2 163)**	**5.7 (2.4 to 9.4)**
Australia	41 299 (34 279 to 49 650)	205 (167 to 255)	6.5 (-0.7 to 14.5)	470 835 (396 677 to 564 393)	1 659 (1 388 to 2 034)	7.8 (4.3 to 12.3)
New Zealand	10 836 (9 233 to 12 653)	292 (245 to 348)	3.5 (-4.5 to 11.3)	114 894 (97 702 to 141 215)	2 231 (1 884 to 2 785)	0.1 (-6.2 to 7.3)
**High-income Asia-Pacific**	**282 319 (234 187 to 329 399)**	**200 (164 to 241)**	**10.9 (4.0 to 17.9)**	**5 867 339 (4 996 817 to 6 851 226)**	**2 257 (1 918 to 2 658)**	**11.2 (9.1 to 13.7)**
Brunei	855 (720 to 1 008)	200 (168 to 238)	-11.8 (-19.8 to -3.5)	7 313 (6 196 to 8 531)	1 652 (1 403 to 1 924)	-16.6 (-20.2 to -12.7)
Japan	195 311 (162 386 to 227 205)	209 (171 to 253)	23.4 (17.9 to 29.5)	4 726 517 (4 029 268 to 5 512 003)	2 605 (2 207 to 3 082)	24.9 (23.1 to 27.0)
South Korea	76 311 (62 788 to 90 620)	180 (148 to 218)	-12.8 (-22.2 to -2.9)	1 021 988 (871 751 to 1 192 249)	1 512 (1 288 to 1 784)	-18.3 (-22.2 to -13.3)
Singapore	9 842 (8 057 to 11 687)	200 (164 to 242)	24.9 (18.9 to 31.8)	111 521 (94 118 to 130 630)	1 689 (1 428 to 1 998)	20.6 (17.2 to 24.6)
**High-income North America**	**433 643 (361 983 to 506 449)**	**132 (110 to 157)**	**-38.1 (-42.3 to -33.9)**	**7 193 870 (6 126 490 to 8 428 848)**	**1 580 (1 339 to 1 852)**	**-38.8 (-41.3 to -37.1)**
Canada	53 291 (44 057 to 62 693)	171 (141 to 204)	-13.2 (-21.1 to -4.6)	669 504 (570 829 to 784 262)	1 437 (1 219 to 1 685)	-11.1 (-14.8 to -7.4)
Greenland	102 (88 to 117)	191 (163 to 220)	-48.6 (-53.6 to -43.5)	1 040 (888 to 1 207)	1 606 (1 373 to 1 870)	-51.4 (-53.7 to -48.7)
USA	380 242 (317 912 to 444 301)	128 (107 to 152)	-40.4 (-44.4 to -36.4)	6 523 200 (5 553 925 to 7 652 530)	1 597 (1 354 to 1 874)	-40.6 (-43.0 to -38.9)
**Southern Latin America**	**133 683 (111 828 to 158 052)**	**226 (187 to 272)**	**14.7 (5.0 to 25.2)**	**1 308 219 (1 110 035 to 1 634 142)**	**1 854 (1 570 to 2 340)**	**2.9 (-1.4 to 8.2)**
Argentina	90 451 (74 393 to 108 975)	222 (181 to 272)	22.2 (10.6 to 33.6)	873 179 (737 608 to 1 086 964)	1 863 (1 574 to 2 338)	10.7 (6.1 to 15.9)
Chile	36 946 (31 899 to 42 260)	243 (206 to 284)	1.0 (-9.4 to 12.5)	367 650 (310 160 to 467 425)	1 861 (1 563 to 2 394)	-13.3 (-19.2 to -6.8)

Location	Incidence (95% UI)			Prevalence (95% UI)		
	2017 counts	2017 age-standardised rates per 100,000	Percentage change in age-standardised rates between 1990 and 2017	2017 counts	2017 age-standardised rates per 100,000	Percentage change in age-standardised rates between 1990 and 2017
Uruguay	6 281<br>(5 184 to 7 530)	213<br>(173 to 262)	15.7<br>(5.2 to 27.9)	67 332<br>(56 536 to 83 028)	1 741<br>(1 457 to 2 172)	4.5<br>(0.0 to 8.6)
**Western Europe**	**449 641<br>(373 154 to 531 728)**	**130<br>(106 to 156)**	**-3.2<br>(-8.3 to 3.1)**	**6 115 599<br>(5 229 409 to 7 082 058)**	**1 099<br>(933 to 1 298)**	**-2.3<br>(-4.1 to -0.5)**
Andorra	86<br>(70 to 103)	135<br>(110 to 166)	-7.5<br>(-14.5 to -0.5)	1 173<br>(999 to 1 373)	1 128<br>(957 to 1 336)	-6.2<br>(-11.2 to -2.3)
Austria	9 437<br>(7 736 to 11 285)	135<br>(109 to 165)	9.8<br>(4.2 to 16.4)	127 304<br>(108 290 to 148 281)	1 121<br>(957 to 1 336)	9.9<br>(5.9 to 14.1)
Belgium	12 286<br>(10 664 to 14 156)	129<br>(110 to 153)	-10.9<br>(-16.6 to -3.7)	150 566<br>(130 254 to 175 417)	1 043<br>(897 to 1 219)	-11.6<br>(-15.3 to -8.0)
Cyprus	1 424<br>(1 167 to 1 697)	131<br>(107 to 162)	5.7<br>(-1.1 to 11.9)	16 742<br>(14 295 to 19 545)	1 094<br>(934 to 1 291)	2.1<br>(-1.3 to 6.0)
Denmark	6 440<br>(5 336 to 7 618)	138<br>(112 to 167)	6.8<br>(-0.1 to 13.7)	82 713<br>(70 270 to 96 333)	1 144<br>(971 to 1 356)	7.8<br>(2.8 to 11.8)
Finland	7 094<br>(6 010 to 8 252)	159<br>(132 to 191)	-10.9<br>(-17.2 to -4.2)	92 739<br>(79 016 to 108 182)	1 300<br>(1 096 to 1 545)	-8.7<br>(-11.9 to -5.1)
France	76 032<br>(62 927 to 90 421)	141<br>(115 to 172)	-4.5<br>(-11.4 to 3.4)	965 878<br>(823 880 to 1 125 637)	1 172<br>(998 to 1 379)	-2.3<br>(-5.8 to 1.6)
Germany	85 467<br>(70 979 to 100 827)	134<br>(109 to 164)	-2.6<br>(-9.9 to 5.4)	1 233 734<br>(1 052 566 to 1 440 324)	1 117<br>(955 to 1 326)	-1.5<br>(-5.7 to 2.5)
Greece	9 603<br>(7 824 to 11 395)	120<br>(97 to 147)	-0.0<br>(-6.6 to 7.4)	138 377<br>(118 413 to 161 554)	997<br>(845 to 1 179)	0.3<br>(-3.0 to 3.6)
Iceland	393<br>(319 to 473)	133<br>(108 to 164)	0.4<br>(-6.3 to 7.4)	4 417<br>(3 782 to 5 153)	1 111<br>(948 to 1 322)	1.6<br>(-2.2 to 5.6)
Ireland	5 724<br>(4 649 to 6 890)	133<br>(108 to 163)	-12.8<br>(-20.6 to -5.0)	63 581<br>(54 041 to 74 085)	1 114<br>(944 to 1 319)	-11.1<br>(-14.8 to -7.5)
Israel	11 182<br>(9 100 to 13 711)	129<br>(105 to 158)	-4.8<br>(-11.4 to 1.9)	100 784<br>(85 697 to 119 787)	1 074<br>(912 to 1 289)	-6.5<br>(-9.8 to -3.2)
Italy	47 325<br>(39 091 to 56 461)	101<br>(83 to 124)	-9.7<br>(-16.9 to -0.4)	682 626<br>(585 168 to 788 371)	830<br>(710 to 985)	-9.8<br>(-12.1 to -6.9)
Luxembourg	688<br>(561 to 823)	138<br>(112 to 167)	8.7<br>(2.3 to 16.5)	8 368<br>(7 097 to 9 724)	1 147<br>(971 to 1 366)	10.0<br>(4.5 to 13.3)
Malta	588<br>(489 to 692)	169<br>(139 to 203)	26.1<br>(19.3 to 33.5)	7 947<br>(6 823 to 9 280)	1 404<br>(1 201 to 1 660)	25.9<br>(22.9 to 29.6)
Netherlands	11 517<br>(9 653 to 13 727)	84<br>(69 to 102)	-16.9<br>(-23.9 to -9.2)	149 879<br>(127 681 to 174 628)	695<br>(589 to 832)	-17.1<br>(-21.6 to -13.0)
Norway	6 675<br>(5 518 to 7 915)	150<br>(122 to 182)	-13.4<br>(-19.1 to -6.8)	118 008<br>(100 173 to 137 937)	1 805<br>(1 528 to 2 134)	-11.3<br>(-14.0 to -8.8)
Portugal	10 331<br>(8 531 to 12 231)	127<br>(103 to 155)	-13.1<br>(-20.8 to -4.2)	151 494<br>(130 405 to 175 926)	1 063<br>(909 to 1 260)	-15.2<br>(-19.0 to -11.5)
Spain	49 995<br>(40 604 to 59 747)	136<br>(110 to 168)	0.9<br>(-7.1 to 10.1)	693 524<br>(594 583 to 800 875)	1 139<br>(971 to 1 347)	2.3<br>(-1.1 to 5.8)
Sweden	12 266<br>(10 124 to 14 583)	148<br>(120 to 180)	2.1<br>(-3.6 to 7.6)	234 501<br>(197 786 to 273 854)	1 832<br>(1 542 to 2 160)	3.3<br>(-0.2 to 6.8)
Switzerland	9 113<br>(7 833 to 10 526)	136<br>(114 to 159)	17.9<br>(9.2 to 28.3)	118 607<br>(101 160 to 138 958)	1 098<br>(937 to 1 315)	14.8<br>(9.3 to 20.7)
United Kingdom	75 509<br>(62 051 to 89 694)	137<br>(111 to 168)	-0.5<br>(-6.8 to 5.7)	966 300<br>(823 728 to 1 129 218)	1 171<br>(991 to 1 384)	0.9<br>(-1.7 to 3.3)
**Latin America and Caribbean**	**499 432<br>(412 826 to 598 881)**	**89<br>(73 to 108)**	**-19.4<br>(-24.3 to -14.3)**	**5 470 429<br>(4 620 037 to 6 606 896)**	**920<br>(778 to 1 111)**	**-28.5<br>(-30.1 to -26.7)**
**Andean Latin America**	**66 869<br>(54 431 to 81 190)**	**107<br>(88 to 130)**	**-3.3<br>(-13.0 to 6.8)**	**558 745<br>(468 384 to 665 076)**	**944<br>(794 to 1 121)**	**-13.9<br>(-17.5 to -9.8)**
Bolivia	11 883<br>(9 687 to 14 487)	100<br>(82 to 121)	-14.1<br>(-24.0 to -2.5)	98 166<br>(83 665 to 115 771)	934<br>(801 to 1 098)	-20.9<br>(-24.4 to -16.5)
Ecuador	18 389<br>(15 039 to 22 307)	110<br>(90 to 133)	-6.3<br>(-16.3 to 4.2)	155 017<br>(130 960 to 184 471)	954<br>(808 to 1 130)	-16.7<br>(-20.9 to -12.3)
Peru	36 597<br>(29 654 to 44 593)	109<br>(89 to 133)	2.4<br>(-8.1 to 13.3)	305 562<br>(254 016 to 366 265)	942<br>(784 to 1 128)	-10.4<br>(-15.1 to -5.6)
**Caribbean**	**57 212<br>(46 775 to 69 028)**	**130<br>(106 to 159)**	**2.4<br>(-5.4 to 10.8)**	**582 228<br>(493 275 to 699 698)**	**1 198<br>(1 015 to 1 442)**	**1.2<br>(-2.0 to 4.3)**
Antigua and Barbuda	111<br>(92 to 131)	140<br>(115 to 168)	-17.4<br>(-26.8 to -8.9)	1 144<br>(954 to 1 377)	1 160<br>(968 to 1 407)	-21.7<br>(-25.4 to -17.5)
The Bahamas	481<br>(406 to 566)	139<br>(115 to 167)	-22.7<br>(-31.1 to -14.3)	4 694<br>(3 973 to 5 605)	1 169<br>(989 to 1 397)	-25.1<br>(-28.5 to -21.9)
Barbados	337<br>(278 to 401)	140<br>(112 to 171)	10.3<br>(-0.8 to 22.0)	4 137<br>(3 465 to 5 029)	1 163<br>(967 to 1 438)	4.9<br>(-0.3 to 11.1)
Belize	519<br>(421 to 640)	127<br>(104 to 155)	7.1<br>(-3.2 to 18.7)	4 272<br>(3 594 to 5 178)	1 167<br>(993 to 1 400)	3.2<br>(-0.4 to 7.5)
Bermuda	83<br>(68 to 99)	166<br>(133 to 206)	49.8<br>(40.4 to 60.4)	1 131<br>(955 to 1 352)	1 351<br>(1 128 to 1 678)	37.4<br>(30.8 to 47.3)
Cuba	14 254<br>(11 715 to 17 176)	156<br>(126 to 195)	21.7<br>(9.4 to 35.9)	175 934<br>(147 332 to 209 975)	1 270<br>(1 056 to 1 546)	14.3<br>(7.8 to 21.9)
Dominica	82<br>(68 to 97)	134<br>(110 to 161)	5.6<br>(-3.7 to 15.0)	897<br>(759 to 1 070)	1 171<br>(988 to 1 414)	1.0<br>(-4.3 to 6.1)
Dominican Republic	13 422<br>(11 004 to 16 199)	130<br>(106 to 157)	14.3<br>(4.7 to 24.4)	118 348<br>(99 621 to 141 905)	1 144<br>(965 to 1 368)	-1.1<br>(-5.1 to 3.5)
Grenada	136<br>(113 to 164)	135<br>(111 to 165)	-16.1<br>(-25.3 to -6.4)	1 476<br>(1 247 to 1 772)	1 214<br>(1 023 to 1 465)	-18.9<br>(-22.7 to -13.6)
Guyana	908<br>(747 to 1 101)	122<br>(101 to 148)	-4.2<br>(-14.3 to 6.2)	8 289<br>(7 093 to 9 864)	1 143<br>(979 to 1 355)	-8.3<br>(-11.5 to -4.5)
Haiti	14 211<br>(11 398 to 17 418)	111<br>(91 to 135)	-23.8<br>(-31.3 to -15.7)	120 754<br>(103 031 to 144 631)	1 136<br>(975 to 1 346)	-26.7<br>(-29.4 to -24.2)
Jamaica	3 544<br>(2 878 to 4 299)	139<br>(112 to 171)	31.8<br>(23.2 to 42.7)	35 913<br>(30 017 to 43 434)	1 233<br>(1 031 to 1 489)	23.0<br>(18.5 to 29.7)
Puerto Rico	4 353<br>(3 606 to 5 228)	150<br>(122 to 186)	33.3<br>(23.7 to 44.1)	55 066<br>(45 890 to 67 160)	1 221<br>(1 005 to 1 527)	29.0<br>(24.7 to 36.1)
Saint Lucia	210<br>(173 to 253)	135<br>(109 to 165)	-10.2<br>(-20.5 to 1.3)	2 301<br>(1 944 to 2 771)	1 178<br>(993 to 1 433)	-14.0<br>(-17.8 to -9.5)
Saint Vincent and the Grenadines	135<br>(111 to 162)	129<br>(105 to 157)	-3.2<br>(-13.8 to 6.6)	1 441<br>(1 217 to 1 726)	1 165<br>(983 to 1 401)	-5.1<br>(-9.1 to 0.4)
Suriname	691<br>(572 to 834)	125<br>(103 to 153)	-10.0<br>(-19.5 to 0.5)	6 836<br>(5 819 to 8 116)	1 149<br>(977 to 1 370)	-14.3<br>(-18.2 to -10.8)
Trinidad and Tobago	1 545<br>(1 280 to 1 844)	125<br>(101 to 153)	3.7<br>(-5.7 to 14.5)	17 115<br>(14 416 to 20 357)	1 086<br>(914 to 1 300)	-1.5<br>(-4.9 to 2.0)
Virgin Islands	131<br>(108 to 157)	149<br>(121 to 184)	18.8<br>(8.6 to 29.7)	1 543<br>(1 292 to 1 846)	1 214<br>(1 010 to 1 485)	9.3<br>(3.8 to 16.0)
**Central Latin America**	**230 986<br>(187 010 to 283 511)**	**93<br>(75 to 114)**	**-17.6<br>(-23.8 to -11.2)**	**2 356 860<br>(1 962 758 to 3 004 398)**	**930<br>(776 to 1 183)**	**-28.3<br>(-30.6 to -24.9)**
Colombia	46 283<br>(36 498 to 57 458)	98<br>(76 to 124)	-19.7<br>(-31.4 to -7.7)	420 433<br>(350 994 to 540 244)	805<br>(670 to 1 037)	-29.6<br>(-34.6 to -24.5)
Costa Rica	4 433<br>(3 518 to 5 550)	107<br>(84 to 136)	14.2<br>(5.4 to 24.9)	41 344<br>(34 415 to 54 694)	846<br>(705 to 1 127)	3.6<br>(-0.4 to 8.9)
El Salvador	5 291<br>(4 148 to 6 696)	91<br>(72 to 116)	-11.7<br>(-22.3 to -0.4)	46 606<br>(38 496 to 60 380)	771<br>(638 to 992)	-25.3<br>(-29.0 to -21.7)
Guatemala	17 027<br>(13 540 to 21 433)	96<br>(77 to 119)	-16.6<br>(-25.4 to -7.5)	133 304<br>(111 302 to 168 467)	870<br>(733 to 1 088)	-24.2<br>(-27.3 to -20.6)
Honduras	10 193<br>(7 948 to 12 930)	98<br>(78 to 122)	22.0<br>(9.9 to 33.6)	80 949<br>(68 041 to 103 007)	941<br>(793 to 1 174)	10.3<br>(6.2 to 14.9)
Mexico	103 991<br>(85 433 to 126 800)	84<br>(69 to 103)	-27.4<br>(-32.2 to -22.1)	1 257 156<br>(1 042 374 to 1 588 421)	1 003<br>(832 to 1 262)	-34.7<br>(-37.0 to -31.2)
Nicaragua	6 393<br>(4 990 to 8 182)	97<br>(76 to 123)	4.9<br>(-5.8 to 16.7)	50 577<br>(41 844 to 66 179)	844<br>(702 to 1 085)	-7.3<br>(-11.2 to -3.0)

Location	Incidence (95% UI)			Prevalence (95% UI)		
	2017 counts	2017 age-standardised rates per 100,000	Percentage change in age-standardised rates between 1990 and 2017	2017 counts	2017 age-standardised rates per 100,000	Percentage change in age-standardised rates between 1990 and 2017
Panama	3 794 (2 986 to 4 763)	100 (78 to 125)	14.8 (5.8 to 24.7)	32 527 (27 278 to 41 350)	819 (687 to 1 042)	1.6 (-2.6 to 5.8)
Venezuela	33 581 (26 704 to 41 459)	114 (90 to 142)	10.5 (0.4 to 20.8)	293 963 (245 105 to 378 751)	946 (791 to 1 217)	-1.2 (-5.2 to 3.0)
**Tropical Latin America**	**144 365 (123 808 to 167 622)**	**69 (59 to 81)**	**-33.4 (-36.4 to -30.1)**	**1 972 596 (1 689 689 to 2 307 851)**	**837 (717 to 982)**	**-37.7 (-38.7 to -36.5)**
Brazil	138 318 (118 914 to 160 521)	68 (58 to 80)	-34.2 (-37.4 to -30.9)	1 922 962 (1 647 527 to 2 249 362)	838 (718 to 984)	-38.1 (-39.1 to -36.9)
Paraguay	6 047 (4 929 to 7 323)	86 (70 to 103)	-8.2 (-16.2 to -0.3)	49 634 (42 410 to 58 489)	769 (660 to 899)	-16.0 (-19.0 to -12.7)
**North Africa and Middle East**	**970 054 (809 558 to 1 167 472)**	**155 (129 to 185)**	**-12.0 (-18.6 to -5.6)**	**8 155 575 (6 907 068 to 9 510 849)**	**1 460 (1 242 to 1 701)**	**-18.4 (-21.3 to -15.6)**
**North Africa and Middle East**	**970 054 (809 558 to 1 167 472)**	**155 (129 to 185)**	**-12.0 (-18.6 to -5.6)**	**8 155 575 (6 907 068 to 9 510 849)**	**1 460 (1 242 to 1 701)**	**-18.4 (-21.3 to -15.6)**
Afghanistan	43 319 (34 546 to 54 349)	115 (94 to 140)	17.1 (8.4 to 25.6)	284 391 (246 426 to 332 100)	1 249 (1 081 to 1 444)	17.8 (13.8 to 22.8)
Algeria	64 036 (53 406 to 76 404)	155 (129 to 187)	-18.0 (-26.2 to -9.6)	568 438 (485 738 to 662 035)	1 423 (1 215 to 1 655)	-24.8 (-28.8 to -20.8)
Bahrain	2 624 (2 119 to 3 153)	180 (148 to 218)	33.4 (25.0 to 42.3)	25 554 (21 510 to 30 218)	1 545 (1 309 to 1 822)	18.3 (13.4 to 23.6)
Egypt	150 049 (125 373 to 181 023)	146 (122 to 175)	-10.5 (-18.6 to -1.7)	1 155 490 (977 792 to 1 342 276)	1 365 (1 162 to 1 580)	-20.5 (-24.0 to -17.1)
Iran	140 466 (117 880 to 166 800)	167 (141 to 199)	-32.0 (-36.7 to -27.6)	1 259 904 (1 069 195 to 1 477 384)	1 462 (1 238 to 1 709)	-39.1 (-41.6 to -37.0)
Iraq	68 583 (55 483 to 84 661)	146 (119 to 177)	-10.0 (-20.3 to 1.1)	507 829 (435 041 to 596 233)	1 440 (1 235 to 1 679)	-11.3 (-15.4 to -7.8)
Jordan	17 259 (14 077 to 20 966)	150 (124 to 181)	-14.5 (-24.9 to -4.0)	121 586 (102 700 to 143 533)	1 323 (1 129 to 1 555)	-22.4 (-27.0 to -17.9)
Kuwait	8 008 (6 576 to 9 701)	183 (150 to 220)	12.3 (2.8 to 20.6)	71 060 (59 090 to 85 515)	1 546 (1 295 to 1 844)	10.1 (4.9 to 16.3)
Lebanon	15 813 (13 141 to 19 113)	176 (147 to 211)	-7.5 (-16.1 to 2.7)	117 676 (98 590 to 139 966)	1 512 (1 272 to 1 786)	-17.0 (-21.5 to -11.3)
Libya	11 018 (9 216 to 13 162)	153 (128 to 183)	-13.6 (-21.1 to -6.3)	88 503 (74 466 to 104 120)	1 326 (1 123 to 1 559)	-20.6 (-24.2 to -16.0)
Morocco	51 347 (43 182 to 61 453)	143 (119 to 170)	-20.0 (-27.3 to -11.9)	478 936 (408 570 to 555 606)	1 335 (1 140 to 1 548)	-26.3 (-29.6 to -22.3)
Palestine	9 417 (7 607 to 11 576)	177 (143 to 215)	46.5 (35.6 to 58.9)	65 839 (55 976 to 77 455)	1 708 (1 460 to 1 989)	41.2 (36.4 to 46.7)
Oman	8 029 (6 423 to 9 999)	166 (134 to 201)	35.5 (25.2 to 45.6)	61 015 (50 572 to 72 980)	1 398 (1 169 to 1 661)	20.8 (15.9 to 26.2)
Qatar	5 023 (4 018 to 6 180)	174 (143 to 210)	12.8 (3.2 to 22.6)	41 974 (35 143 to 49 961)	1 444 (1 220 to 1 698)	3.9 (-1.1 to 9.5)
Saudi Arabia	61 754 (51 627 to 74 331)	169 (141 to 202)	-23.1 (-30.5 to -15.4)	474 632 (398 902 to 561 117)	1 391 (1 176 to 1 638)	-31.7 (-35.5 to -28.0)
Sudan	59 568 (48 991 to 72 398)	135 (113 to 160)	-20.7 (-26.9 to -13.7)	554 994 (467 958 to 658 019)	1 871 (1 595 to 2 172)	-25.0 (-28.3 to -21.9)
Syria	32 050 (25 880 to 39 976)	167 (138 to 203)	23.3 (10.5 to 35.8)	246 406 (209 435 to 290 419)	1 505 (1 283 to 1 762)	12.3 (6.6 to 18.2)
Tunisia	17 721 (14 681 to 21 228)	159 (130 to 190)	-10.5 (-19.8 to -0.9)	175 589 (149 035 to 205 637)	1 402 (1 192 to 1 638)	-20.0 (-24.4 to -15.5)
Turkey	141 919 (116 004 to 172 206)	179 (147 to 219)	38.0 (29.2 to 48.0)	1 382 321 (1 155 233 to 1 623 241)	1 569 (1 312 to 1 841)	17.8 (11.9 to 24.7)
United Arab Emirates	16 156 (12 831 to 19 605)	168 (139 to 201)	-1.9 (-10.5 to 6.7)	160 064 (133 562 to 190 172)	1 388 (1 166 to 1 638)	-11.5 (-16.5 to -5.6)
Yemen	44 990 (36 961 to 54 667)	137 (115 to 164)	-28.7 (-34.3 to -22.9)	305 757 (261 937 to 355 925)	1 359 (1 172 to 1 562)	-32.6 (-35.4 to -29.8)
**South Asia**	**2 053 339 (1 686 233 to 2 506 760)**	**110 (91 to 133)**	**0.1 (-7.0 to 6.8)**	**20 409 524 (17 554 220 to 23 823 865)**	**1 237 (1 064 to 1 442)**	**-1.6 (-4.1 to 0.9)**
**South Asia**	**2 053 339 (1 686 233 to 2 506 760)**	**110 (91 to 133)**	**0.1 (-7.0 to 6.8)**	**20 409 524 (17 554 220 to 23 823 865)**	**1 237 (1 064 to 1 442)**	**-1.6 (-4.1 to 0.9)**
Bangladesh	179 400 (145 990 to 223 235)	109 (89 to 134)	-3.5 (-14.6 to 7.8)	1 630 435 (1 402 709 to 1 911 028)	1 102 (949 to 1 293)	-8.3 (-12.9 to -2.8)
Bhutan	1 119 (910 to 1 375)	107 (88 to 131)	-1.8 (-12.6 to 8.1)	9 649 (8 283 to 11 227)	1 087 (936 to 1 261)	-9.8 (-14.6 to -5.2)
India	1 589 628 (1 307 428 to 1 941 097)	110 (91 to 134)	-1.5 (-8.2 to 5.1)	16 500 527 (14 203 250 to 19 285 633)	1 264 (1 090 to 1 473)	-2.5 (-5.1 to -0.1)
Nepal	30 694 (25 026 to 37 840)	97 (80 to 119)	-2.5 (-11.5 to 7.1)	274 906 (236 375 to 318 921)	1 029 (888 to 1 192)	-4.9 (-8.8 to -0.2)
Pakistan	252 498 (206 047 to 310 720)	111 (91 to 134)	20.1 (11.4 to 29.2)	1 994 006 (1 702 237 to 2 316 468)	1 161 (996 to 1 347)	18.1 (14.0 to 24.1)
**Southeast Asia, East Asia, and Oceania**	**1 570 370 (1 268 175 to 1 904 021)**	**78 (63 to 96)**	**23.9 (15.9 to 31.8)**	**23 411 324 (19 634 321 to 27 685 884)**	**918 (766 to 1 089)**	**17.2 (13.1 to 22.0)**
**East Asia**	**1 385 196 (1 121 266 to 1 679 792)**	**107 (86 to 131)**	**43.7 (34.4 to 54.0)**	**21 434 056 (17 943 830 to 25 366 091)**	**1 162 (967 to 1 384)**	**28.4 (23.4 to 34.2)**
China	1 331 789 (1 078 274 to 1 615 908)	108 (87 to 133)	46.0 (36.7 to 56.2)	20 680 371 (17 312 143 to 24 472 798)	1 179 (981 to 1 404)	29.6 (24.6 to 35.6)
North Korea	16 053 (13 037 to 19 601)	69 (55 to 85)	7.9 (-0.3 to 16.6)	201 050 (170 888 to 235 286)	675 (575 to 793)	7.1 (3.7 to 12.2)
Taiwan (Province of China)	15 040 (12 141 to 18 053)	80 (64 to 99)	-23.4 (-32.6 to -12.3)	207 344 (173 633 to 247 015)	684 (570 to 814)	-28.9 (-33.1 to -24.3)
**Oceania**	**11 339 (9 449 to 13 745)**	**83 (70 to 100)**	**3.0 (-3.6 to 10.1)**	**91 658 (79 259 to 106 019)**	**871 (756 to 1 001)**	**2.6 (-0.8 to 6.2)**
American Samoa	54 (44 to 67)	96 (79 to 117)	18.9 (10.7 to 27.8)	476 (403 to 560)	917 (777 to 1 078)	13.8 (9.1 to 18.5)
Federated States of Micronesia	89 (73 to 109)	81 (67 to 98)	15.0 (5.6 to 24.8)	798 (684 to 925)	845 (727 to 977)	11.2 (5.9 to 16.7)
Fiji	1 033 (882 to 1 210)	111 (95 to 130)	1.2 (-6.4 to 9.5)	9 940 (8 612 to 11 474)	1 120 (970 to 1 293)	-1.3 (-5.5 to 3.6)
Guam	164 (134 to 199)	101 (82 to 122)	30.1 (22.8 to 37.8)	1 610 (1 349 to 1 902)	916 (768 to 1 081)	25.7 (21.2 to 30.5)
Kiribati	101 (81 to 125)	79 (63 to 97)	49.5 (38.0 to 60.1)	884 (763 to 1 026)	878 (758 to 1 018)	48.0 (42.6 to 54.1)
Marshall Islands	50 (41 to 60)	82 (67 to 98)	18.1 (8.7 to 27.5)	419 (357 to 488)	835 (711 to 972)	16.0 (11.0 to 21.2)
Northern Mariana Islands	41 (33 to 49)	102 (83 to 122)	11.9 (3.3 to 20.5)	469 (392 to 551)	890 (738 to 1 047)	7.8 (1.5 to 14.1)
Papua New Guinea	8 050 (6 675 to 9 819)	79 (66 to 96)	4.6 (-3.2 to 12.7)	62 837 (54 477 to 72 680)	826 (717 to 950)	3.0 (-1.2 to 7.2)
Samoa	191 (154 to 236)	91 (74 to 111)	19.8 (11.0 to 28.8)	1 572 (1 355 to 1 812)	932 (805 to 1 078)	15.3 (11.1 to 19.8)
Solomon Islands	550 (451 to 673)	79 (65 to 96)	8.2 (-1.0 to 17.6)	4 370 (3 772 to 5 073)	848 (732 to 984)	9.2 (4.7 to 14.8)
Tonga	123 (102 to 147)	116 (99 to 137)	10.2 (2.8 to 18.3)	1 056 (912 to 1 223)	1 144 (989 to 1 326)	6.4 (2.7 to 10.6)
Vanuatu	268 (221 to 327)	87 (72 to 105)	8.2 (0.4 to 15.7)	2 176 (1 875 to 2 503)	922 (795 to 1 060)	8.9 (3.6 to 14.2)
**Southeast Asia**	**173 835 (138 150 to 214 303)**	**27 (21 to 33)**	**-19.4 (-25.2 to -13.3)**	**1 885 610 (1 593 040 to 2 237 486)**	**281 (238 to 333)**	**-29.0 (-31.6 to -25.4)**
Cambodia	5 308 (4 310 to 6 518)	32 (26 to 39)	-36.6 (-43.8 to -29.1)	47 578 (40 773 to 54 826)	322 (277 to 369)	-39.6 (-42.6 to -36.2)
Indonesia	43 568 (32 094 to 59 063)	17 (13 to 23)	-44.8 (-52.0 to -36.1)	624 895 (513 873 to 759 744)	241 (198 to 292)	-48.6 (-51.2 to -44.7)

Location	Incidence (95% UI)			Prevalence (95% UI)		
	2017 counts	2017 age-standardised rates per 100,000	Percentage change in age-standardised rates between 1990 and 2017	2017 counts	2017 age-standardised rates per 100,000	Percentage change in age-standardised rates between 1990 and 2017
Laos	2 342 (1 910 to 2 872)	32 (26 to 39)	-46.3 (-52.2 to -40.1)	20 143 (17 400 to 23 394)	327 (283 to 379)	-48.7 (-51.5 to -46.2)
Malaysia	10 669 (8 530 to 13 038)	35 (28 to 43)	-19.0 (-27.4 to -10.6)	90 001 (76 310 to 106 055)	297 (253 to 350)	-28.1 (-31.7 to -23.1)
Maldives	157 (126 to 194)	34 (28 to 42)	-50.9 (-57.0 to -44.4)	1 294 (1 089 to 1 535)	287 (243 to 339)	-58.1 (-60.7 to -55.1)
Mauritius	545 (459 to 642)	46 (38 to 56)	-55.3 (-60.8 to -49.5)	6 228 (5 273 to 7 283)	407 (344 to 477)	-59.1 (-61.6 to -56.3)
Myanmar	13 333 (10 485 to 16 708)	25 (20 to 32)	8.3 (-0.6 to 17.0)	135 878 (115 942 to 159 947)	257 (220 to 302)	2.2 (-1.6 to 6.9)
Philippines	28 656 (22 866 to 35 670)	27 (21 to 33)	-14.7 (-23.1 to -6.6)	238 089 (204 145 to 279 652)	252 (217 to 295)	-19.4 (-23.0 to -14.2)
Sri Lanka	8 451 (6 949 to 10 131)	41 (33 to 49)	-29.5 (-37.0 to -21.6)	82 934 (69 810 to 96 500)	350 (294 to 408)	-36.4 (-40.4 to -31.8)
Seychelles	59 (50 to 69)	58 (50 to 68)	-18.4 (-24.4 to -12.4)	551 (475 to 639)	490 (422 to 571)	-24.4 (-27.7 to -20.6)
Thailand	23 476 (19 170 to 28 300)	37 (30 to 46)	5.4 (-3.3 to 15.6)	274 615 (233 511 to 323 278)	316 (269 to 373)	-7.3 (-11.6 to -1.7)
Timor-Leste	425 (340 to 533)	31 (25 to 38)	-38.9 (-45.9 to -31.2)	3 213 (2 749 to 3 776)	309 (265 to 359)	-43.4 (-46.0 to -40.7)
Vietnam	36 617 (30 020 to 43 990)	39 (32 to 48)	33.1 (23.7 to 44.9)	357 709 (305 685 to 421 749)	353 (301 to 418)	13.5 (8.4 to 20.3)
**Sub-Saharan Africa**	**1 413 990 (1 152 708 to 1 741 406)**	**135 (114 to 162)**	**-10.4 (-16.5 to -4.3)**	**9 904 005 (8 478 636 to 11 515 704)**	**1 347 (1 162 to 1 545)**	**-10.1 (-12.4 to -7.7)**
**Central sub-Saharan Africa**	**157 036 (127 446 to 194 089)**	**126 (106 to 151)**	**-6.5 (-13.9 to 0.4)**	**1 081 118 (929 091 to 1 257 250)**	**1 239 (1 072 to 1 422)**	**-6.5 (-9.3 to -3.3)**
Angola	36 622 (29 898 to 45 201)	129 (109 to 154)	-19.9 (-27.6 to -12.7)	236 389 (203 080 to 273 292)	1 243 (1 079 to 1 421)	-20.8 (-23.9 to -17.3)
Central African Republic	5 759 (4 728 to 7 081)	120 (101 to 143)	-5.4 (-12.6 to 1.7)	42 955 (37 147 to 50 200)	1 192 (1 035 to 1 382)	-2.5 (-6.1 to 1.7)
Congo (Brazzaville)	6 439 (5 306 to 7 830)	128 (108 to 152)	-14.6 (-22.2 to -7.3)	49 080 (42 048 to 57 415)	1 224 (1 059 to 1 425)	-14.0 (-17.4 to -10.6)
DR Congo	104 087 (84 370 to 129 518)	126 (105 to 151)	-1.0 (-8.6 to 6.6)	723 415 (621 257 to 840 087)	1 241 (1 074 to 1 430)	-0.8 (-4.0 to 2.9)
Equatorial Guinea	1 770 (1 435 to 2 204)	129 (108 to 154)	-10.0 (-18.4 to -1.6)	10 487 (8 922 to 12 432)	1 155 (992 to 1 339)	-18.6 (-22.1 to -15.1)
Gabon	2 358 (1 959 to 2 863)	136 (115 to 163)	-16.7 (-23.8 to -9.7)	18 791 (16 203 to 22 032)	1 290 (1 118 to 1 501)	-15.9 (-18.8 to -12.4)
**Eastern sub-Saharan Africa**	**614 040 (499 660 to 762 348)**	**153 (129 to 182)**	**-9.6 (-15.8 to -3.1)**	**4 342 276 (3 731 137 to 5 063 428)**	**1 585 (1 370 to 1 821)**	**-7.5 (-9.8 to -5.0)**
Burundi	17 323 (14 064 to 21 332)	155 (130 to 184)	-19.9 (-27.3 to -11.8)	116 743 (100 218 to 135 860)	1 521 (1 317 to 1 739)	-18.4 (-21.3 to -15.0)
Comoros	1 181 (978 to 1 429)	161 (136 to 192)	-23.6 (-30.5 to -16.3)	9 618 (8 338 to 11 099)	1 575 (1 371 to 1 801)	-24.4 (-26.9 to -20.7)
Djibouti	1 782 (1 480 to 2 167)	160 (135 to 191)	-18.5 (-25.2 to -10.7)	14 317 (12 366 to 16 530)	1 526 (1 324 to 1 744)	-19.9 (-22.5 to -16.8)
Eritrea	9 613 (7 886 to 11 837)	161 (136 to 191)	-16.8 (-23.2 to -9.4)	67 460 (57 935 to 78 250)	1 554 (1 350 to 1 780)	-16.2 (-19.5 to -12.9)
Ethiopia	137 872 (110 708 to 173 116)	131 (109 to 158)	-19.9 (-26.1 to -13.1)	938 727 (809 707 to 1 087 402)	1 323 (1 147 to 1 512)	-17.4 (-20.3 to -14.2)
Kenya	88 475 (72 244 to 109 252)	180 (151 to 215)	4.7 (-1.4 to 11.0)	820 740 (698 237 to 963 761)	2 386 (2 045 to 2 763)	5.5 (2.9 to 8.2)
Madagascar	43 174 (35 167 to 53 156)	164 (139 to 195)	-17.4 (-25.5 to -9.4)	296 813 (255 944 to 343 638)	1 599 (1 389 to 1 828)	-18.7 (-21.5 to -15.4)
Malawi	25 404 (20 468 to 31 824)	142 (119 to 171)	-2.7 (-10.2 to 6.7)	171 202 (145 966 to 198 201)	1 382 (1 190 to 1 582)	-2.0 (-5.5 to 2.5)
Mozambique	48 690 (39 499 to 60 069)	159 (134 to 187)	-8.6 (-16.0 to -0.1)	303 499 (258 788 to 355 370)	1 487 (1 285 to 1 710)	-15.0 (-18.2 to -10.8)
Rwanda	19 947 (16 274 to 24 649)	156 (130 to 187)	-22.8 (-30.4 to -15.3)	141 451 (121 080 to 164 723)	1 504 (1 304 to 1 727)	-23.2 (-26.6 to -18.9)
Somalia	27 414 (22 285 to 33 471)	161 (136 to 191)	-7.3 (-13.9 to -0.9)	186 857 (161 055 to 214 905)	1 616 (1 407 to 1 837)	-5.8 (-9.1 to -2.6)
South Sudan	18 746 (15 418 to 22 787)	188 (159 to 219)	-4.5 (-10.8 to 1.6)	122 734 (105 858 to 141 432)	1 809 (1 585 to 2 065)	-2.5 (-5.2 to 0.4)
Tanzania	88 041 (71 465 to 108 588)	157 (131 to 187)	1.6 (-5.5 to 8.8)	602 711 (517 967 to 701 098)	1 534 (1 325 to 1 763)	2.1 (-1.8 to 5.5)
Uganda	59 462 (47 754 to 74 943)	147 (124 to 177)	-3.7 (-10.8 to 4.3)	369 942 (316 258 to 431 063)	1 440 (1 242 to 1 653)	0.1 (-3.9 to 4.0)
Zambia	26 533 (21 473 to 32 863)	149 (125 to 177)	-12.0 (-19.1 to -3.8)	176 736 (151 352 to 207 541)	1 444 (1 252 to 1 660)	-8.8 (-12.5 to -5.1)
**Southern sub-Saharan Africa**	**137 781 (115 442 to 166 331)**	**174 (148 to 208)**	**-21.9 (-27.1 to -16.9)**	**1 114 603 (960 254 to 1 290 061)**	**1 588 (1 373 to 1 836)**	**-25.7 (-28.3 to -22.9)**
Botswana	4 204 (3 484 to 5 119)	178 (149 to 213)	-8.9 (-15.9 to -2.2)	32 835 (28 007 to 38 005)	1 615 (1 384 to 1 855)	-11.1 (-15.2 to -7.5)
Lesotho	3 762 (3 186 to 4 457)	190 (163 to 222)	12.1 (4.7 to 19.7)	28 469 (24 655 to 32 703)	1 746 (1 515 to 1 999)	8.6 (4.3 to 13.7)
Namibia	4 309 (3 607 to 5 235)	179 (152 to 213)	-13.5 (-20.7 to -6.0)	32 204 (27 779 to 37 251)	1 667 (1 442 to 1 920)	-15.9 (-19.2 to -12.2)
South Africa	99 021 (82 757 to 119 206)	176 (147 to 210)	-28.6 (-33.8 to -23.4)	832 956 (717 120 to 968 809)	1 573 (1 358 to 1 826)	-32.9 (-35.6 to -30.2)
Swaziland	2 079 (1 747 to 2 495)	182 (155 to 214)	-5.0 (-11.9 to 1.6)	14 303 (12 356 to 16 478)	1 653 (1 435 to 1 893)	-9.5 (-13.0 to -6.7)
Zimbabwe	24 406 (20 139 to 29 467)	163 (138 to 192)	12.6 (5.7 to 19.4)	173 837 (150 535 to 200 731)	1 582 (1 382 to 1 818)	18.0 (14.6 to 22.4)
**Western sub-Saharan Africa**	**505 133 (409 118 to 624 274)**	**114 (95 to 137)**	**-2.1 (-8.8 to 5.0)**	**3 366 008 (2 877 751 to 3 911 617)**	**1 099 (945 to 1 260)**	**-4.7 (-7.4 to -1.7)**
Benin	13 336 (10 770 to 16 489)	113 (95 to 136)	-6.6 (-14.3 to 1.3)	89 979 (76 958 to 105 488)	1 122 (965 to 1 293)	-8.4 (-11.6 to -4.4)
Burkina Faso	25 735 (21 088 to 31 338)	122 (103 to 143)	-10.4 (-17.4 to -2.5)	172 576 (148 627 to 201 488)	1 181 (1 026 to 1 356)	-11.9 (-15.1 to -8.7)
Cameroon	31 302 (25 306 to 38 741)	110 (92 to 132)	-10.3 (-17.5 to -3.1)	212 492 (182 263 to 247 450)	1 065 (919 to 1 226)	-11.5 (-14.9 to -7.6)
Cape Verde	661 (541 to 811)	119 (98 to 143)	-1.6 (-9.3 to 6.3)	5 555 (4 708 to 6 492)	1 079 (919 to 1 255)	-9.5 (-13.3 to -5.8)
Chad	17 922 (14 467 to 22 183)	118 (99 to 140)	4.0 (-2.7 to 10.4)	111 972 (95 857 to 130 357)	1 170 (1 013 to 1 341)	2.7 (-0.4 to 6.3)
Cote d'Ivoire	28 824 (23 574 to 35 301)	114 (96 to 136)	-7.9 (-15.4 to -0.5)	208 336 (180 075 to 240 369)	1 122 (974 to 1 282)	-7.3 (-10.2 to -4.1)
The Gambia	2 480 (2 002 to 3 082)	114 (95 to 137)	-4.9 (-11.8 to 2.2)	16 852 (14 467 to 19 629)	1 095 (946 to 1 259)	-6.0 (-8.9 to -2.5)
Ghana	35 819 (29 483 to 43 965)	116 (97 to 138)	-2.9 (-9.9 to 4.7)	266 021 (228 117 to 309 918)	1 102 (950 to 1 271)	-7.0 (-10.5 to -3.1)
Guinea	13 831 (11 273 to 17 037)	116 (98 to 139)	-8.2 (-15.6 to -0.9)	96 437 (83 272 to 110 674)	1 156 (1 002 to 1 317)	-10.7 (-13.6 to -7.2)
Guinea-Bissau	2 023 (1 638 to 2 502)	108 (90 to 129)	-17.4 (-24.4 to -9.9)	13 998 (12 023 to 16 227)	1 073 (928 to 1 231)	-17.7 (-21.1 to -14.2)
Liberia	5 215 (4 207 to 6 506)	108 (89 to 130)	-5.1 (-13.0 to 2.6)	36 869 (31 925 to 42 657)	1 061 (921 to 1 218)	-7.2 (-12.0 to -3.3)
Mali	24 101 (19 462 to 29 560)	117 (98 to 139)	-9.1 (-17.4 to -1.3)	154 198 (132 725 to 180 431)	1 138 (988 to 1 312)	-11.9 (-16.0 to -8.0)
Mauritania	4 533 (3 648 to 5 615)	113 (93 to 136)	-11.3 (-19.5 to -3.6)	31 726 (27 131 to 36 861)	1 088 (937 to 1 250)	-12.7 (-16.2 to -8.8)

Location	Incidence (95% UI)			Prevalence (95% UI)		
	2017 counts	2017 age-standardised rates per 100,000	Percentage change in age-standardised rates between 1990 and 2017	2017 counts	2017 age-standardised rates per 100,000	Percentage change in age-standardised rates between 1990 and 2017
Niger	25 073<br>(20 081 to 31 118)	118<br>(99 to 141)	-9.0<br>(-17.0 to -1.0)	153 609<br>(132 365 to 178 604)	1 175<br>(1 019 to 1 347)	-10.6<br>(-13.8 to -7.2)
Nigeria	240 091<br>(192 869 to 299 498)	113<br>(93 to 136)	4.3<br>(-2.6 to 12.9)	1 548 028<br>(1 315 914 to 1 799 406)	1 073<br>(920 to 1 233)	0.4<br>(-3.3 to 4.2)
Sao Tome and Principe	238<br>(189 to 298)	112<br>(90 to 136)	23.9<br>(16.5 to 32.8)	1 784<br>(1 515 to 2 089)	1 115<br>(951 to 1 290)	15.6<br>(11.7 to 20.2)
Senegal	16 678<br>(13 506 to 20 588)	113<br>(94 to 136)	-10.4<br>(-17.7 to -3.3)	121 399<br>(104 644 to 140 908)	1 118<br>(972 to 1 286)	-11.4<br>(-14.9 to -6.6)
Sierra Leone	8 906<br>(7 286 to 10 956)	112<br>(94 to 133)	-7.1<br>(-14.3 to 1.0)	62 464<br>(53 598 to 72 409)	1 093<br>(945 to 1 253)	-9.6<br>(-13.1 to -5.9)
Togo	8 361<br>(6 819 to 10 334)	108<br>(90 to 131)	-6.0<br>(-13.4 to 1.8)	61 678<br>(52 612 to 71 662)	1 071<br>(922 to 1 234)	-7.6<br>(-11.3 to -3.6)
