# Supplementary material for: Epidemiology of injuries from fire, heat and hot substances: global, regional and national morbidity and mortality estimates from the Global Burden of Disease 2017 study
Source: Inj Prev. 2019 Dec 18;26(Suppl 1):i36–45. doi: 10.1136/injuryprev-2019-043299 (PMC7571358; doi:10.1136/injuryprev-2019-043299)
Supplement: Supplementary data [file injuryprev-2019-043299supp004.pdf]

Table 2: Mortality for 2017 and percentage change of age-standardised rates between 1990 and 2017 by location for fire, heat, and hot substances

Location	Mortality (95% UI)		
	2017 counts	2017 age-standardised rates per 100,000	Percentage change in age-standardised rates between 1990 and 2017
**Global**	**120 632 (101 630 to 129 383)**	**1.6 (1.3 to 1.7)**	**-46.6 (-49.7 to -38.8)**
**Low SDI**	**29 548 (23 482 to 33 505)**	**3.0 (2.4 to 3.3)**	**-45.9 (-52.3 to -33.7)**
**Low-middle SDI**	**29 584 (22 729 to 33 290)**	**2.0 (1.6 to 2.3)**	**-43.8 (-50.3 to -32.8)**
**Middle SDI**	**23 369 (18 393 to 25 776)**	**1.2 (0.9 to 1.3)**	**-53.0 (-56.1 to -43.8)**
**High-middle SDI**	**22 495 (20 687 to 23 584)**	**1.4 (1.3 to 1.5)**	**-51.1 (-54.9 to -44.2)**
**High SDI**	**15 374 (15 078 to 15 664)**	**0.8 (0.8 to 0.8)**	**-55.2 (-56.1 to -54.2)**
**Central Europe, Eastern Europe, and Central Asia**	**14 031 (13 610 to 14 503)**	**2.7 (2.6 to 2.8)**	**-30.8 (-32.8 to -28.3)**
**Central Asia**	**2 277 (2 082 to 2 555)**	**2.7 (2.4 to 3.0)**	**-49.1 (-53.3 to -43.8)**
Armenia	37 (34 to 39)	1.0 (1.0 to 1.1)	-81.1 (-83.1 to -79.1)
Azerbaijan	393 (293 to 608)	4.1 (3.0 to 6.1)	-37.1 (-54.0 to -9.7)
Georgia	178 (162 to 196)	3.8 (3.4 to 4.1)	-38.4 (-44.6 to -30.7)
Kazakhstan	434 (394 to 475)	2.4 (2.2 to 2.6)	-53.1 (-57.8 to -48.2)
Kyrgyzstan	96 (88 to 105)	1.7 (1.6 to 1.8)	-61.9 (-65.7 to -57.8)
Mongolia	101 (57 to 153)	3.1 (1.8 to 4.5)	-10.4 (-52.0 to 58.3)
Tajikistan	243 (185 to 351)	2.8 (2.2 to 3.9)	-52.6 (-62.8 to -24.0)
Turkmenistan	116 (101 to 131)	2.4 (2.1 to 2.7)	-61.4 (-66.3 to -56.1)
Uzbekistan	680 (605 to 766)	2.3 (2.1 to 2.6)	-45.3 (-52.0 to -37.5)
**Central Europe**	**1 754 (1 680 to 1 826)**	**1.0 (1.0 to 1.0)**	**-51.6 (-53.6 to -49.1)**
Albania	17 (13 to 25)	0.5 (0.4 to 0.7)	-51.8 (-64.3 to -32.0)
Bosnia and Herzegovina	41 (33 to 51)	0.8 (0.7 to 1.0)	-37.3 (-58.2 to -8.9)
Bulgaria	109 (100 to 118)	1.0 (0.9 to 1.1)	-47.4 (-52.2 to -42.2)
Croatia	46 (42 to 49)	0.6 (0.5 to 0.6)	-69.6 (-72.2 to -67.0)
Czech Republic	147 (137 to 159)	0.8 (0.7 to 0.9)	-36.6 (-41.4 to -30.9)
Hungary	130 (121 to 141)	0.8 (0.7 to 0.8)	-68.6 (-71.2 to -65.9)
Macedonia	15 (11 to 18)	0.5 (0.4 to 0.7)	-35.8 (-54.1 to -17.5)
Montenegro	9 (7 to 10)	1.0 (0.9 to 1.2)	-34.0 (-43.1 to -21.1)
Poland	631 (580 to 679)	1.1 (1.0 to 1.2)	-41.8 (-46.5 to -36.9)
Romania	435 (407 to 464)	1.5 (1.4 to 1.6)	-56.2 (-59.7 to -52.5)
Serbia	109 (88 to 126)	0.8 (0.7 to 0.9)	-44.2 (-52.5 to -27.7)
Slovakia	53 (43 to 61)	0.7 (0.6 to 0.8)	-52.5 (-63.1 to -43.6)
Slovenia	12 (11 to 14)	0.3 (0.3 to 0.4)	-62.8 (-66.6 to -58.4)

Location	Mortality (95% UI)		
	2017 counts	2017 age-standardised rates per 100,000	Percentage change in age-standardised rates between 1990 and 2017
**Eastern Europe**	**10 000 (9 714 to 10 259)**	**3.6 (3.4 to 3.6)**	**-19.2 (-21.5 to -16.9)**
Belarus	623 (573 to 678)	4.6 (4.3 to 5.0)	0.0 (-10.1 to 11.5)
Estonia	57 (50 to 65)	2.9 (2.5 to 3.3)	-65.8 (-70.7 to -60.0)
Latvia	120 (107 to 134)	4.1 (3.6 to 4.5)	-50.5 (-56.0 to -43.6)
Lithuania	78 (71 to 85)	1.8 (1.7 to 2.0)	-56.6 (-60.7 to -52.3)
Moldova	119 (111 to 127)	2.5 (2.3 to 2.6)	-54.6 (-58.7 to -50.0)
Russian Federation	7 063 (6 844 to 7 250)	3.6 (3.5 to 3.7)	-15.5 (-19.2 to -12.7)
Ukraine	1 941 (1 820 to 2 072)	3.3 (3.1 to 3.5)	-22.8 (-29.1 to -16.1)
**High-income**	**15 261 (14 963 to 15 526)**	**0.8 (0.8 to 0.8)**	**-52.8 (-53.7 to -51.8)**
**Australasia**	**185 (170 to 200)**	**0.4 (0.4 to 0.5)**	**-58.2 (-61.8 to -54.5)**
Australia	162 (148 to 177)	0.4 (0.4 to 0.5)	-57.2 (-61.5 to -52.8)
New Zealand	23 (21 to 24)	0.4 (0.4 to 0.4)	-63.2 (-66.0 to -60.4)
**High-income Asia-Pacific**	**2 556 (2 457 to 2 645)**	**0.7 (0.6 to 0.7)**	**-54.8 (-56.8 to -52.9)**
Brunei	6 (5 to 7)	1.7 (1.5 to 2.0)	-46.4 (-55.6 to -36.4)
Japan	1 923 (1 853 to 1 992)	0.6 (0.6 to 0.7)	-42.5 (-45.0 to -40.0)
South Korea	614 (563 to 662)	0.9 (0.8 to 0.9)	-68.1 (-70.8 to -65.5)
Singapore	13 (12 to 14)	0.2 (0.2 to 0.2)	-58.5 (-62.1 to -54.7)
**High-income North America**	**6 023 (5 864 to 6 169)**	**1.1 (1.1 to 1.2)**	**-50.3 (-51.8 to -48.8)**
Canada	515 (484 to 550)	0.9 (0.8 to 0.9)	-55.6 (-58.6 to -52.1)
Greenland	2 (2 to 3)	4.4 (3.8 to 5.4)	-59.8 (-68.1 to -37.8)
USA	5 505 (5 354 to 5 650)	1.2 (1.1 to 1.2)	-49.7 (-51.3 to -48.1)
**Southern Latin America**	**1 203 (1 121 to 1 304)**	**1.6 (1.5 to 1.7)**	**-43.1 (-47.3 to -38.1)**
Argentina	756 (688 to 835)	1.5 (1.4 to 1.7)	-38.3 (-44.2 to -31.4)
Chile	362 (324 to 404)	1.7 (1.5 to 1.9)	-56.6 (-61.3 to -51.2)
Uruguay	85 (76 to 94)	1.7 (1.6 to 1.9)	-15.5 (-24.9 to -5.9)
**Western Europe**	**5 295 (5 140 to 5 440)**	**0.6 (0.6 to 0.6)**	**-56.5 (-58.1 to -55.1)**
Andorra	1 (1 to 1)	0.6 (0.5 to 0.8)	-46.6 (-60.2 to -27.3)
Austria	88 (82 to 94)	0.5 (0.5 to 0.6)	-25.0 (-30.9 to -18.5)
Belgium	182 (169 to 195)	0.8 (0.8 to 0.9)	-46.0 (-50.4 to -41.2)
Cyprus	12 (8 to 15)	0.7 (0.5 to 0.9)	-55.9 (-62.0 to -47.2)
Denmark	99 (92 to 106)	1.0 (0.9 to 1.0)	-20.3 (-27.0 to -13.2)
Finland	73 (68 to 79)	0.8 (0.8 to 0.9)	-61.0 (-64.5 to -57.1)

Location	Mortality (95% UI)		
	2017 counts	2017 age-standardised rates per 100,000	Percentage change in age-standardised rates between 1990 and 2017
France	1 211<br>(1 134 to 1 292)	0.9<br>(0.8 to 0.9)	-60.6<br>(-63.4 to -57.7)
Germany	777<br>(706 to 855)	0.5<br>(0.4 to 0.5)	-66.8<br>(-70.0 to -63.2)
Greece	142<br>(132 to 153)	0.6<br>(0.6 to 0.7)	-48.8<br>(-53.0 to -44.6)
Iceland	3<br>(3 to 3)	0.6<br>(0.6 to 0.6)	-48.1<br>(-52.2 to -43.6)
Ireland	41<br>(38 to 45)	0.6<br>(0.6 to 0.7)	-67.8<br>(-71.1 to -64.7)
Israel	78<br>(72 to 84)	0.7<br>(0.6 to 0.7)	-47.5<br>(-51.6 to -42.9)
Italy	907<br>(847 to 966)	0.5<br>(0.5 to 0.6)	-51.3<br>(-54.6 to -47.5)
Luxembourg	5<br>(4 to 5)	0.5<br>(0.4 to 0.5)	-59.7<br>(-64.0 to -54.8)
Malta	3<br>(2 to 3)	0.4<br>(0.3 to 0.4)	-45.5<br>(-50.7 to -40.7)
Netherlands	153<br>(142 to 163)	0.5<br>(0.4 to 0.5)	-56.9<br>(-60.4 to -53.4)
Norway	92<br>(89 to 95)	1.0<br>(1.0 to 1.0)	-48.4<br>(-50.9 to -46.4)
Portugal	236<br>(227 to 246)	1.3<br>(1.2 to 1.3)	-29.1<br>(-33.3 to -25.1)
Spain	343<br>(321 to 368)	0.4<br>(0.4 to 0.4)	-65.5<br>(-68.2 to -62.6)
Sweden	176<br>(166 to 187)	0.9<br>(0.8 to 0.9)	-35.4<br>(-39.6 to -31.0)
Switzerland	41<br>(38 to 45)	0.3<br>(0.3 to 0.3)	-46.0<br>(-51.2 to -40.5)
United Kingdom	627<br>(615 to 637)	0.5<br>(0.5 to 0.6)	-52.1<br>(-53.2 to -51.0)
**Latin America and Caribbean**	**5 483<br>(5 250 to 5 730)**	**1.0<br>(0.9 to 1.0)**	**-63.3<br>(-64.8 to -60.8)**
**Andean Latin America**	**780<br>(667 to 960)**	**1.3<br>(1.2 to 1.6)**	**-59.3<br>(-65.8 to -45.5)**
Bolivia	185<br>(136 to 236)	1.9<br>(1.5 to 2.3)	-63.4<br>(-73.2 to -45.6)
Ecuador	216<br>(197 to 236)	1.4<br>(1.3 to 1.5)	-53.3<br>(-58.0 to -48.3)
Peru	379<br>(288 to 517)	1.2<br>(0.9 to 1.6)	-59.8<br>(-70.5 to -41.0)
**Caribbean**	**751<br>(587 to 917)**	**1.6<br>(1.2 to 2.0)**	**-59.2<br>(-66.7 to -50.3)**
Antigua and Barbuda	2<br>(2 to 2)	2.0<br>(1.8 to 2.2)	-57.8<br>(-62.4 to -53.1)
The Bahamas	9<br>(8 to 10)	2.5<br>(2.3 to 2.8)	-54.9<br>(-60.1 to -49.8)
Barbados	5<br>(5 to 6)	1.2<br>(1.1 to 1.3)	-57.5<br>(-61.7 to -53.2)
Belize	4<br>(4 to 5)	1.3<br>(1.3 to 1.4)	-57.7<br>(-62.0 to -52.9)
Bermuda	0<br>(0 to 0)	0.2<br>(0.2 to 0.3)	-68.6<br>(-72.2 to -65.1)
Cuba	110<br>(99 to 122)	0.6<br>(0.6 to 0.7)	-78.5<br>(-80.6 to -76.0)
Dominica	2<br>(1 to 2)	1.9<br>(1.8 to 2.1)	-45.7<br>(-51.2 to -39.2)
Dominican Republic	132<br>(106 to 167)	1.4<br>(1.1 to 1.7)	-46.0<br>(-56.6 to -27.8)
Grenada	3<br>(3 to 3)	2.0<br>(1.8 to 2.2)	-61.6<br>(-65.5 to -57.4)
Guyana	13<br>(12 to 15)	2.1<br>(1.9 to 2.4)	-54.8<br>(-60.5 to -49.0)

Location	Mortality (95% UI)		
	2017 counts	2017 age-standardised rates per 100,000	Percentage change in age-standardised rates between 1990 and 2017
Haiti	323 (182 to 468)	3.6 (2.2 to 5.0)	-59.1 (-72.2 to -39.8)
Jamaica	35 (30 to 39)	1.2 (1.0 to 1.3)	-9.6 (-23.2 to 4.1)
Puerto Rico	44 (41 to 47)	0.7 (0.6 to 0.7)	-60.4 (-63.7 to -56.9)
Saint Lucia	3 (3 to 3)	1.6 (1.4 to 1.7)	-64.7 (-68.0 to -61.2)
Saint Vincent and the Grenadines	2 (2 to 2)	1.8 (1.7 to 1.9)	-56.1 (-60.0 to -51.8)
Suriname	10 (9 to 11)	1.9 (1.7 to 2.0)	-59.1 (-64.0 to -53.9)
Trinidad and Tobago	24 (21 to 28)	1.7 (1.4 to 1.9)	-44.1 (-52.8 to -34.5)
Virgin Islands	2 (2 to 2)	1.3 (1.1 to 1.4)	-52.1 (-59.5 to -45.0)
**Central Latin America**	**2 211 (2 092 to 2 285)**	**0.9 (0.9 to 1.0)**	**-62.3 (-65.1 to -60.9)**
Colombia	217 (194 to 242)	0.4 (0.4 to 0.5)	-80.2 (-82.5 to -77.9)
Costa Rica	42 (39 to 46)	0.9 (0.8 to 0.9)	-35.3 (-41.8 to -29.2)
El Salvador	50 (37 to 74)	0.8 (0.6 to 1.2)	-61.8 (-74.2 to -37.2)
Guatemala	263 (235 to 292)	2.0 (1.8 to 2.2)	-41.6 (-48.1 to -35.1)
Honduras	46 (35 to 59)	0.6 (0.5 to 0.8)	-50.0 (-61.3 to -34.2)
Mexico	1 322 (1 229 to 1 365)	1.2 (1.1 to 1.2)	-60.1 (-63.6 to -58.5)
Nicaragua	27 (22 to 38)	0.5 (0.4 to 0.7)	-69.1 (-77.9 to -49.8)
Panama	25 (22 to 27)	0.6 (0.6 to 0.7)	-46.7 (-52.2 to -40.5)
Venezuela	219 (189 to 253)	0.8 (0.7 to 0.9)	-53.1 (-59.3 to -46.0)
**Tropical Latin America**	**1 742 (1 696 to 1 788)**	**0.8 (0.8 to 0.8)**	**-66.8 (-68.2 to -65.5)**
Brazil	1 684 (1 644 to 1 728)	0.8 (0.8 to 0.8)	-67.3 (-68.6 to -65.9)
Paraguay	57 (47 to 72)	1.0 (0.8 to 1.2)	-42.7 (-53.3 to -30.1)
**North Africa and Middle East**	**10 525 (7 384 to 12 427)**	**1.9 (1.3 to 2.2)**	**-60.2 (-66.0 to -50.8)**
**North Africa and Middle East**	**10 525 (7 384 to 12 427)**	**1.9 (1.3 to 2.2)**	**-60.2 (-66.0 to -50.8)**
Afghanistan	485 (383 to 614)	2.0 (1.6 to 2.3)	-40.4 (-58.5 to 43.0)
Algeria	736 (373 to 1 131)	1.9 (1.0 to 2.9)	-58.4 (-65.6 to -48.0)
Bahrain	10 (8 to 14)	1.0 (0.7 to 1.4)	-31.4 (-62.0 to 7.5)
Egypt	2 002 (1 382 to 2 583)	2.3 (1.7 to 2.9)	-45.9 (-54.6 to -31.6)
Iran	1 940 (1 648 to 2 157)	2.5 (2.1 to 2.8)	-68.9 (-75.9 to -53.2)
Iraq	429 (361 to 512)	1.0 (0.9 to 1.3)	-72.6 (-81.3 to -37.5)
Jordan	132 (110 to 162)	1.5 (1.3 to 1.8)	-63.1 (-69.9 to -51.4)
Kuwait	32 (29 to 35)	0.9 (0.8 to 0.9)	-40.3 (-46.7 to -33.7)
Lebanon	112 (53 to 192)	1.5 (0.7 to 2.4)	-71.9 (-80.2 to -63.2)

Location	Mortality (95% UI)		
	2017 counts	2017 age-standardised rates per 100,000	Percentage change in age-standardised rates between 1990 and 2017
Libya	135<br>(40 to 218)	2.2<br>(0.7 to 3.5)	-45.6<br>(-68.1 to -17.7)
Morocco	851<br>(412 to 1 239)	2.5<br>(1.2 to 3.7)	-55.0<br>(-64.2 to -42.7)
Palestine	26<br>(19 to 41)	0.8<br>(0.6 to 1.0)	-63.0<br>(-74.2 to -40.5)
Oman	18<br>(14 to 25)	0.7<br>(0.4 to 0.8)	-55.8<br>(-69.6 to -29.6)
Qatar	20<br>(13 to 28)	1.0<br>(0.7 to 1.3)	-53.0<br>(-68.3 to -26.4)
Saudi Arabia	710<br>(489 to 938)	2.5<br>(1.7 to 3.3)	-59.1<br>(-77.0 to -30.7)
Sudan	1 121<br>(483 to 1 752)	3.1<br>(1.4 to 4.7)	-63.5<br>(-73.4 to -40.7)
Syria	137<br>(105 to 181)	0.9<br>(0.7 to 1.2)	-66.8<br>(-77.3 to -50.9)
Tunisia	173<br>(84 to 284)	1.5<br>(0.7 to 2.5)	-60.4<br>(-69.5 to -46.5)
Turkey	420<br>(319 to 490)	0.5<br>(0.4 to 0.6)	-63.9<br>(-76.1 to -48.1)
United Arab Emirates	145<br>(100 to 197)	2.9<br>(1.3 to 4.3)	-46.1<br>(-66.2 to -21.4)
Yemen	881<br>(402 to 1 411)	3.5<br>(1.6 to 5.6)	-58.6<br>(-71.6 to -20.1)
**South Asia**	**31 684<br>(23 098 to 36 828)**	**2.0<br>(1.5 to 2.3)**	**-44.5<br>(-50.9 to -34.2)**
**South Asia**	**31 684<br>(23 098 to 36 828)**	**2.0<br>(1.5 to 2.3)**	**-44.5<br>(-50.9 to -34.2)**
Bangladesh	1 671<br>(1 337 to 2 276)	1.3<br>(1.1 to 1.6)	-74.3<br>(-81.0 to -56.9)
Bhutan	10<br>(6 to 15)	1.2<br>(0.8 to 1.8)	-64.2<br>(-74.7 to -48.3)
India	27 027<br>(18 672 to 31 468)	2.2<br>(1.6 to 2.5)	-39.8<br>(-48.9 to -26.0)
Nepal	373<br>(256 to 538)	1.5<br>(1.1 to 2.1)	-54.8<br>(-66.4 to -38.4)
Pakistan	2 603<br>(1 909 to 3 899)	1.4<br>(1.1 to 2.1)	-38.1<br>(-53.5 to -16.6)
**Southeast Asia, East Asia, and Oceania**	**17 752<br>(14 803 to 19 545)**	**0.8<br>(0.7 to 0.9)**	**-58.7<br>(-62.4 to -47.7)**
**East Asia**	**11 545<br>(9 244 to 12 783)**	**0.7<br>(0.6 to 0.8)**	**-64.6<br>(-68.3 to -52.3)**
China	10 836<br>(8 528 to 12 024)	0.7<br>(0.6 to 0.8)	-64.9<br>(-68.7 to -51.9)
North Korea	312<br>(200 to 454)	1.2<br>(0.8 to 1.8)	-22.0<br>(-43.8 to 3.4)
Taiwan (Province of China)	211<br>(199 to 225)	0.7<br>(0.6 to 0.7)	-75.1<br>(-76.8 to -73.3)
**Oceania**	**339<br>(208 to 452)**	**3.2<br>(2.2 to 4.1)**	**-21.6<br>(-35.9 to -4.1)**
American Samoa	1<br>(0 to 1)	1.2<br>(1.0 to 1.4)	-33.8<br>(-50.7 to -7.6)
Federated States of Micronesia	1<br>(1 to 2)	1.8<br>(1.2 to 2.4)	-33.0<br>(-53.1 to -9.9)
Fiji	31<br>(26 to 36)	4.1<br>(3.5 to 4.7)	-13.7<br>(-39.8 to 10.6)
Guam	1<br>(1 to 1)	0.7<br>(0.6 to 0.9)	-27.3<br>(-43.1 to 1.2)
Kiribati	1<br>(1 to 1)	0.9<br>(0.7 to 1.1)	-16.5<br>(-37.2 to 13.7)
Marshall Islands	1<br>(1 to 1)	2.3<br>(1.5 to 3.0)	-20.7<br>(-46.9 to 6.8)
Northern Mariana Islands	0<br>(0 to 1)	1.1<br>(0.9 to 1.3)	-41.5<br>(-55.4 to -21.6)

Location	Mortality (95% UI)		
	2017 counts	2017 age-standardised rates per 100,000	Percentage change in age-standardised rates between 1990 and 2017
Papua New Guinea	260<br>(139 to 363)	3.3<br>(1.9 to 4.4)	-23.1<br>(-38.4 to 0.5)
Samoa	3<br>(2 to 4)	1.6<br>(1.1 to 2.5)	-19.0<br>(-37.3 to 3.8)
Solomon Islands	12<br>(9 to 17)	2.4<br>(1.9 to 3.4)	-25.9<br>(-44.3 to -1.7)
Tonga	3<br>(2 to 4)	3.5<br>(2.8 to 4.4)	-21.7<br>(-38.5 to -0.9)
Vanuatu	5<br>(3 to 8)	2.3<br>(1.5 to 3.3)	-17.8<br>(-41.1 to 14.6)
**Southeast Asia**	**5 869<br>(5 183 to 6 489)**	**1.0<br>(0.9 to 1.1)**	**-47.3<br>(-52.4 to -38.0)**
Cambodia	255<br>(190 to 347)	2.0<br>(1.5 to 2.7)	-62.0<br>(-72.6 to -40.8)
Indonesia	1 759<br>(1 473 to 1 931)	0.8<br>(0.7 to 0.9)	-55.2<br>(-64.4 to -35.8)
Laos	136<br>(92 to 182)	2.3<br>(1.6 to 3.1)	-64.7<br>(-75.3 to -37.7)
Malaysia	389<br>(283 to 447)	1.5<br>(1.1 to 1.8)	-42.7<br>(-55.0 to -32.0)
Maldives	4<br>(4 to 6)	1.2<br>(1.1 to 1.5)	-76.4<br>(-82.1 to -53.9)
Mauritius	27<br>(25 to 30)	2.0<br>(1.8 to 2.2)	-70.0<br>(-72.7 to -66.9)
Myanmar	321<br>(246 to 462)	0.7<br>(0.5 to 1.0)	-55.5<br>(-68.1 to -32.6)
Philippines	1 187<br>(1 055 to 1 335)	1.6<br>(1.4 to 1.8)	-23.6<br>(-32.6 to -13.5)
Sri Lanka	419<br>(338 to 529)	2.0<br>(1.6 to 2.4)	-49.0<br>(-58.2 to -35.9)
Seychelles	4<br>(3 to 4)	3.8<br>(3.0 to 4.3)	-28.0<br>(-47.0 to -4.1)
Thailand	812<br>(586 to 978)	1.0<br>(0.7 to 1.2)	-32.0<br>(-60.8 to 4.4)
Timor-Leste	18<br>(12 to 25)	1.8<br>(1.2 to 2.4)	-64.6<br>(-77.1 to -46.2)
Vietnam	531<br>(443 to 657)	0.6<br>(0.5 to 0.7)	-44.2<br>(-56.5 to -26.8)
**Sub-Saharan Africa**	**25 897<br>(20 839 to 29 327)**	**3.7<br>(3.0 to 4.3)**	**-38.8<br>(-46.2 to -24.6)**
**Central sub-Saharan Africa**	**3 152<br>(1 938 to 4 169)**	**3.7<br>(2.4 to 4.7)**	**-38.9<br>(-53.0 to -14.9)**
Angola	711<br>(549 to 916)	3.8<br>(2.9 to 4.6)	-55.1<br>(-67.1 to -19.1)
Central African Republic	177<br>(79 to 282)	5.3<br>(2.7 to 7.7)	-26.9<br>(-47.5 to 4.8)
Congo (Brazzaville)	115<br>(71 to 155)	3.6<br>(2.1 to 4.7)	-45.7<br>(-57.7 to -22.5)
DR Congo	2 093<br>(1 106 to 2 927)	3.6<br>(2.0 to 4.8)	-31.6<br>(-48.8 to -8.3)
Equatorial Guinea	20<br>(13 to 30)	2.7<br>(1.8 to 4.0)	-64.5<br>(-78.3 to -31.9)
Gabon	35<br>(23 to 52)	2.9<br>(1.9 to 4.1)	-47.8<br>(-61.5 to -19.9)
**Eastern sub-Saharan Africa**	**9 574<br>(7 988 to 11 487)**	**4.0<br>(3.4 to 4.7)**	**-43.6<br>(-51.9 to -24.4)**
Burundi	331<br>(231 to 438)	5.2<br>(3.7 to 6.8)	-42.2<br>(-56.7 to -19.1)
Comoros	19<br>(15 to 26)	3.9<br>(3.0 to 5.0)	-47.3<br>(-60.3 to -26.9)
Djibouti	26<br>(17 to 42)	3.8<br>(2.6 to 5.8)	-46.4<br>(-62.6 to -20.3)
Eritrea	176<br>(125 to 219)	5.4<br>(3.9 to 6.6)	-42.6<br>(-55.9 to -19.6)

Location	Mortality (95% UI)		
	2017 counts	2017 age-standardised rates per 100,000	Percentage change in age-standardised rates between 1990 and 2017
Ethiopia	2 013<br>(1 651 to 2 659)	3.4<br>(2.8 to 4.4)	-56.7<br>(-66.8 to -30.6)
Kenya	1 003<br>(836 to 1 371)	3.8<br>(3.3 to 5.0)	-35.9<br>(-52.2 to -17.0)
Madagascar	682<br>(506 to 888)	4.4<br>(3.3 to 5.8)	-36.9<br>(-48.8 to -20.8)
Malawi	418<br>(341 to 519)	3.8<br>(3.2 to 4.6)	-42.2<br>(-58.4 to 1.4)
Mozambique	899<br>(719 to 1 139)	5.2<br>(4.3 to 6.4)	-40.3<br>(-52.8 to -18.5)
Rwanda	319<br>(234 to 490)	4.2<br>(3.2 to 6.1)	-52.5<br>(-65.2 to -27.0)
Somalia	550<br>(376 to 817)	5.6<br>(4.0 to 8.4)	-30.4<br>(-52.6 to 14.7)
South Sudan	428<br>(265 to 639)	6.5<br>(4.1 to 9.8)	-23.8<br>(-44.9 to 16.2)
Tanzania	1 460<br>(1 179 to 1 933)	3.7<br>(3.2 to 4.6)	-31.7<br>(-45.0 to -5.1)
Uganda	823<br>(619 to 1 103)	3.7<br>(2.9 to 4.8)	-39.4<br>(-54.0 to -16.4)
Zambia	422<br>(342 to 530)	4.2<br>(3.5 to 4.9)	-46.2<br>(-57.6 to -20.9)
**Southern sub-Saharan Africa**	**2 815<br>(2 324 to 3 413)**	**4.3<br>(3.6 to 5.1)**	**-41.2<br>(-51.3 to -33.2)**
Botswana	63<br>(43 to 99)	3.7<br>(2.7 to 5.2)	-42.0<br>(-55.4 to -21.4)
Lesotho	108<br>(80 to 136)	7.2<br>(5.3 to 9.0)	-5.8<br>(-45.9 to 32.3)
Namibia	75<br>(58 to 105)	4.2<br>(3.3 to 5.6)	-46.2<br>(-56.4 to -29.0)
South Africa	1 928<br>(1 534 to 2 451)	3.9<br>(3.2 to 4.8)	-49.7<br>(-57.9 to -39.6)
Swaziland	48<br>(36 to 62)	5.9<br>(4.4 to 7.5)	-23.1<br>(-45.2 to 0.8)
Zimbabwe	592<br>(429 to 738)	6.6<br>(4.5 to 8.5)	7.0<br>(-30.6 to 38.7)
**Western sub-Saharan Africa**	**10 355<br>(7 124 to 13 044)**	**3.4<br>(2.5 to 4.1)**	**-31.1<br>(-43.3 to -12.5)**
Benin	260<br>(173 to 364)	3.5<br>(2.4 to 4.8)	-33.7<br>(-50.2 to -14.2)
Burkina Faso	739<br>(556 to 976)	4.7<br>(3.8 to 5.7)	-33.8<br>(-46.1 to -15.4)
Cameroon	562<br>(325 to 749)	3.4<br>(1.9 to 4.6)	-33.0<br>(-53.3 to -11.3)
Cape Verde	7<br>(6 to 8)	1.4<br>(1.2 to 1.7)	-49.1<br>(-62.7 to -25.1)
Chad	549<br>(340 to 825)	4.9<br>(3.3 to 7.9)	-8.8<br>(-31.3 to 18.3)
Cote d'Ivoire	742<br>(581 to 930)	4.7<br>(3.9 to 5.7)	-30.1<br>(-45.4 to -7.7)
The Gambia	48<br>(36 to 65)	3.7<br>(2.7 to 4.6)	-27.7<br>(-46.9 to -5.9)
Ghana	639<br>(523 to 797)	3.4<br>(2.8 to 4.0)	-22.3<br>(-41.9 to -1.1)
Guinea	363<br>(238 to 494)	4.5<br>(3.0 to 6.1)	-30.9<br>(-50.6 to -3.9)
Guinea-Bissau	48<br>(33 to 63)	4.4<br>(2.8 to 5.5)	-38.8<br>(-55.0 to -15.3)
Liberia	87<br>(54 to 131)	3.1<br>(1.9 to 4.7)	-44.2<br>(-61.2 to -13.3)
Mali	775<br>(466 to 1 178)	4.5<br>(2.9 to 7.4)	-33.7<br>(-51.4 to -4.0)
Mauritania	68<br>(45 to 98)	2.7<br>(1.8 to 3.7)	-41.9<br>(-56.1 to -20.8)

Location	Mortality (95% UI)		
	2017 counts	2017 age-standardised rates per 100,000	Percentage change in age-standardised rates between 1990 and 2017
Niger	657<br>(402 to 1 107)	4.4<br>(2.8 to 8.8)	-39.1<br>(-58.4 to -6.1)
Nigeria	4 085<br>(2 183 to 5 957)	2.6<br>(1.6 to 3.5)	-34.5<br>(-50.7 to -7.1)
Sao Tome and Principe	1<br>(1 to 2)	0.7<br>(0.4 to 1.0)	-34.2<br>(-56.6 to -7.1)
Senegal	372<br>(257 to 563)	3.8<br>(2.6 to 5.6)	-26.2<br>(-43.3 to -2.4)
Sierra Leone	217<br>(124 to 295)	4.0<br>(2.4 to 5.3)	-33.2<br>(-53.3 to -6.4)
Togo	134<br>(86 to 179)	3.0<br>(1.9 to 3.9)	-35.5<br>(-53.1 to -13.1)
