# Supplementary material for: Epidemiology of injuries from fire, heat and hot substances: global, regional and national morbidity and mortality estimates from the Global Burden of Disease 2017 study
Source: Inj Prev. 2019 Dec 18;26(Suppl 1):i36–45. doi: 10.1136/injuryprev-2019-043299 (PMC7571358; doi:10.1136/injuryprev-2019-043299)
Supplement: Supplementary data [file injuryprev-2019-043299supp005.pdf]

Table 3: YLLs, YLDs, and DALYs for 2017 and percentage change of age-standardised rates between 1990 and 2017 by location for fire, heat, and hot substances

Location	YLLs (95% UI)			YLDs (95% UI)			DALYs (95% UI)		
	2017 counts	2017 age-standardised rates per 100,000	Percentage change in age-standardised rates between 1990 and 2017	2017 counts	2017 age-standardised rates per 100,000	Percentage change in age-standardised rates between 1990 and 2017	2017 counts	2017 age-standardised rates per 100,000	Percentage change in age-standardised rates between 1990 and 2017
**Global**	5 286 270 (4 308 900 to 5 836 389)	71 (58 to 79)	-50.8 (-55.8 to -39.3)	3 177 003 (2 210 387 to 4 396 730)	40 (28 to 55)	-24.4 (-29.4 to -19.3)	8 463 273 (7 034 048 to 9 881 099)	111 (93 to 129)	-43.7 (-49.3 to -34.1)
**Low SDI**	1 642 466 (1 295 333 to 1 890 515)	124 (98 to 141)	-50.1 (-59.0 to -28.3)	801 023 (570 067 to 1 040 725)	71 (51 to 92)	-23.7 (-27.6 to -19.2)	2 443 489 (2 055 446 to 2 826 710)	196 (165 to 226)	-42.9 (-50.9 to -26.7)
**Low-middle SDI**	1 551 209 (1 161 619 to 1 794 468)	91 (68 to 104)	-49.4 (-55.7 to -34.0)	799 992 (572 733 to 1 049 107)	50 (36 to 65)	-27.8 (-32.7 to -21.9)	2 351 200 (1 917 408 to 2 725 990)	140 (115 to 162)	-43.4 (-49.8 to -31.7)
**Middle SDI**	947 456 (728 799 to 1 083 180)	47 (36 to 54)	-59.4 (-64.5 to -49.0)	536 794 (343 751 to 797 623)	24 (15 to 35)	-32.6 (-42.8 to -21.9)	1 484 250 (1 164 928 to 1 817 093)	70 (55 to 86)	-53.1 (-58.6 to -44.0)
**High-middle SDI**	797 343 (733 325 to 850 617)	56 (51 to 60)	-58.7 (-62.1 to -50.8)	555 979 (346 879 to 846 839)	34 (21 to 51)	-38.5 (-49.1 to -28.0)	1 353 322 (1 136 617 to 1 652 248)	90 (76 to 108)	-52.9 (-58.0 to -45.8)
**High SDI**	338 742 (332 242 to 346 298)	26 (26 to 27)	-63.3 (-64.3 to -62.3)	474 173 (282 555 to 753 441)	33 (20 to 51)	-15.4 (-18.5 to -13.2)	812 915 (621 279 to 1 090 124)	59 (46 to 78)	-46.4 (-51.7 to -40.9)
**Central Europe, Eastern Europe, and Central Asia**	507 349 (490 191 to 527 592)	114 (109 to 119)	-40.5 (-42.9 to -37.1)	282 527 (185 212 to 416 053)	57 (38 to 83)	-30.9 (-38.4 to -23.4)	789 876 (688 601 to 923 740)	171 (150 to 197)	-37.6 (-40.6 to -34.0)
**Central Asia**	114 697 (103 643 to 128 808)	124 (112 to 139)	-55.4 (-60.1 to -49.0)	75 016 (51 712 to 104 990)	82 (56 to 115)	-38.0 (-44.3 to -31.1)	189 713 (162 374 to 221 870)	206 (176 to 241)	-49.8 (-54.3 to -44.7)
Armenia	1 184 (1 078 to 1 291)	39 (36 to 44)	-86.0 (-88.2 to -83.4)	2 422 (1 557 to 3 558)	69 (45 to 101)	-45.3 (-53.9 to -35.5)	3 606 (2 762 to 4 726)	108 (84 to 140)	-73.4 (-78.0 to -68.0)
Azerbaijan	20 053 (14 401 to 31 632)	203 (144 to 315)	-44.7 (-62.3 to -8.4)	9 741 (6 582 to 13 825)	88 (59 to 125)	-47.6 (-54.9 to -39.3)	29 795 (22 853 to 41 525)	292 (222 to 406)	-45.7 (-57.9 to -22.2)
Georgia	5 932 (5 369 to 6 649)	149 (134 to 169)	-49.1 (-55.6 to -41.0)	3 273 (2 202 to 4 739)	75 (51 to 107)	-32.2 (-37.1 to -27.4)	9 205 (7 893 to 10 753)	224 (193 to 261)	-44.4 (-49.8 to -38.0)
Kazakhstan	19 622 (17 307 to 22 258)	105 (93 to 119)	-60.4 (-65.9 to -54.1)	12 277 (8 115 to 17 852)	66 (44 to 97)	-42.6 (-51.0 to -33.8)	31 900 (27 037 to 38 042)	172 (146 to 205)	-55.0 (-60.1 to -49.6)
Kyrgyzstan	4 878 (4 339 to 5 511)	76 (68 to 84)	-67.0 (-71.4 to -62.0)	5 086 (3 503 to 6 944)	84 (58 to 116)	-22.8 (-29.7 to -16.0)	9 964 (8 317 to 11 886)	160 (134 to 192)	-52.7 (-58.1 to -47.2)
Mongolia	6 472 (2 930 to 10 811)	184 (85 to 301)	-14.7 (-61.9 to 65.3)	3 620 (2 557 to 4 865)	109 (77 to 147)	-22.6 (-30.8 to -13.4)	10 092 (6 251 to 14 640)	292 (183 to 421)	-17.9 (-49.2 to 27.1)
Tajikistan	14 975 (10 886 to 22 058)	146 (110 to 211)	-55.4 (-65.7 to -22.7)	9 522 (6 727 to 12 633)	114 (80 to 150)	-38.4 (-43.5 to -33.0)	24 498 (18 981 to 32 436)	260 (204 to 336)	-49.3 (-56.6 to -28.9)
Turkmenistan	6 375 (5 502 to 7 322)	123 (106 to 140)	-64.8 (-70.0 to -58.8)	4 329 (2 989 to 6 034)	87 (60 to 122)	-54.8 (-60.7 to -47.9)	10 704 (8 954 to 12 558)	210 (176 to 247)	-61.2 (-65.6 to -56.3)
Uzbekistan	35 205 (31 264 to 40 033)	108 (96 to 122)	-54.4 (-60.7 to -46.9)	24 745 (17 035 to 34 415)	78 (54 to 110)	-36.7 (-44.3 to -28.3)	59 949 (50 511 to 69 779)	186 (156 to 217)	-48.3 (-53.8 to -42.5)
**Central Europe**	47 672 (45 481 to 49 745)	35 (33 to 36)	-59.5 (-61.8 to -56.6)	55 781 (35 246 to 84 320)	38 (24 to 57)	-33.4 (-42.7 to -24.4)	103 453 (82 742 to 131 517)	73 (59 to 92)	-49.0 (-54.1 to -43.3)
Albania	561 (424 to 799)	21 (16 to 29)	-60.2 (-72.4 to -28.6)	1 232 (780 to 1 842)	37 (24 to 56)	-41.8 (-53.7 to -29.3)	1 793 (1 288 to 2 458)	58 (43 to 78)	-50.1 (-60.0 to -36.5)
Bosnia and Herzegovina	1 084 (901 to 1 313)	27 (23 to 33)	-41.9 (-58.3 to -10.4)	1 778 (1 139 to 2 624)	42 (27 to 61)	-41.5 (-52.6 to -28.5)	2 863 (2 161 to 3 745)	68 (52 to 88)	-41.6 (-50.6 to -29.9)
Bulgaria	2 979 (2 709 to 3 243)	39 (34 to 43)	-56.0 (-61.5 to -49.9)	3 359 (2 095 to 5 075)	36 (23 to 55)	-30.9 (-39.5 to -22.1)	6 338 (5 053 to 8 064)	75 (61 to 93)	-46.6 (-52.3 to -40.5)
Croatia	850 (783 to 918)	14 (13 to 16)	-71.1 (-74.1 to -67.9)	1 360 (858 to 2 067)	24 (16 to 36)	-20.8 (-24.3 to -17.8)	2 210 (1 693 to 2 883)	39 (29 to 51)	-51.9 (-57.8 to -46.2)
Czech Republic	2 970 (2 740 to 3 207)	22 (20 to 23)	-49.8 (-54.3 to -44.5)	6 259 (3 961 to 9 409)	47 (30 to 70)	16.2 (11.0 to 21.5)	9 229 (6 978 to 12 464)	68 (51 to 92)	-17.8 (-26.4 to -9.0)
Hungary	3 115 (2 866 to 3 389)	24 (22 to 26)	-72.4 (-75.2 to -69.3)	4 651 (2 930 to 6 986)	37 (24 to 56)	-22.4 (-30.0 to -15.3)	7 767 (6 045 to 10 157)	61 (47 to 80)	-54.4 (-60.4 to -47.7)
Macedonia	450 (328 to 548)	21 (15 to 26)	-45.0 (-63.3 to -22.5)	985 (618 to 1 472)	37 (23 to 55)	-30.2 (-42.6 to -17.3)	1 435 (1 049 to 1 956)	57 (43 to 77)	-36.3 (-49.4 to -24.2)
Montenegro	229 (197 to 275)	32 (27 to 40)	-48.2 (-57.9 to -32.8)	285 (180 to 426)	38 (24 to 56)	-13.6 (-20.8 to -6.8)	514 (403 to 663)	70 (55 to 90)	-34.0 (-43.4 to -23.4)
Poland	19 024 (17 413 to 20 598)	40 (37 to 43)	-47.4 (-52.0 to -42.3)	18 883 (11 998 to 28 430)	39 (25 to 58)	-33.2 (-43.5 to -22.7)	37 907 (30 561 to 47 350)	79 (64 to 98)	-41.2 (-46.8 to -35.1)
Romania	12 137 (11 279 to 12 950)	56 (52 to 61)	-65.4 (-68.9 to -61.5)	9 278 (5 828 to 13 990)	37 (23 to 56)	-55.2 (-62.7 to -47.3)	21 415 (17 918 to 26 140)	93 (79 to 112)	-61.9 (-65.6 to -58.0)
Serbia	2 498 (2 119 to 2 992)	23 (19 to 29)	-62.6 (-71.6 to -42.8)	4 058 (2 544 to 6 118)	37 (23 to 56)	-22.7 (-32.4 to -12.9)	6 556 (4 975 to 8 623)	60 (45 to 79)	-45.0 (-56.5 to -31.3)
Slovakia	1 499 (1 143 to 1 795)	25 (18 to 30)	-56.2 (-67.4 to -45.2)	2 526 (1 584 to 3 794)	37 (24 to 56)	-12.1 (-20.0 to -4.8)	4 025 (3 016 to 5 345)	62 (47 to 81)	-37.2 (-47.3 to -28.1)
Slovenia	275 (249 to 303)	10 (9 to 11)	-67.4 (-71.2 to -63.2)	1 127 (717 to 1 700)	42 (27 to 63)	19.6 (15.4 to 23.9)	1 402 (989 to 1 974)	52 (37 to 73)	-21.4 (-31.4 to -11.9)
**Eastern Europe**	344 980 (334 120 to 354 335)	143 (138 to 147)	-31.5 (-33.7 to -29.3)	151 731 (95 832 to 229 673)	56 (35 to 84)	-31.6 (-40.0 to -23.6)	496 710 (440 094 to 572 734)	199 (178 to 226)	-31.5 (-34.4 to -28.7)
Belarus	19 914 (18 112 to 21 839)	174 (157 to 191)	-21.5 (-31.3 to -9.9)	7 527 (4 690 to 11 396)	60 (38 to 91)	-29.6 (-38.2 to -21.9)	27 442 (24 168 to 31 806)	234 (207 to 270)	-23.8 (-31.2 to -15.4)
Estonia	1 715 (1 486 to 1 982)	109 (94 to 126)	-71.7 (-76.0 to -66.4)	1 037 (645 to 1 568)	59 (37 to 89)	-41.6 (-47.0 to -37.1)	2 752 (2 306 to 3 315)	169 (143 to 200)	-65.4 (-69.7 to -60.5)
Latvia	3 641 (3 232 to 4 092)	156 (138 to 176)	-59.2 (-64.3 to -53.1)	1 579 (976 to 2 396)	60 (38 to 89)	-39.6 (-45.5 to -34.5)	5 219 (4 454 to 6 108)	215 (186 to 251)	-55.2 (-60.2 to -49.7)
Lithuania	2 394 (2 175 to 2 632)	70 (64 to 76)	-62.8 (-66.8 to -58.5)	2 098 (1 320 to 3 190)	56 (35 to 84)	-16.1 (-22.6 to -9.8)	4 492 (3 667 to 5 594)	126 (104 to 155)	-50.6 (-56.1 to -44.9)
Moldova	3 851 (3 573 to 4 159)	95 (86 to 106)	-63.9 (-68.6 to -58.0)	2 970 (1 964 to 4 285)	64 (43 to 93)	-32.9 (-40.9 to -25.0)	6 821 (5 811 to 8 127)	159 (136 to 188)	-55.6 (-60.6 to -49.6)
Russian Federation	245 156 (236 789 to 252 068)	145 (140 to 149)	-28.3 (-31.2 to -26.1)	103 954 (65 603 to 157 020)	56 (35 to 84)	-33.2 (-41.7 to -25.0)	349 110 (309 751 to 401 138)	201 (180 to 229)	-29.7 (-32.5 to -26.8)
Ukraine	68 310 (63 520 to 73 380)	137 (126 to 148)	-32.1 (-39.1 to -24.4)	32 565 (20 494 to 50 308)	55 (35 to 84)	-26.8 (-35.6 to -18.2)	100 874 (87 316 to 118 844)	192 (169 to 222)	-30.6 (-36.6 to -24.8)
**High-income**	338 427 (331 415 to 345 251)	28 (28 to 29)	-60.6 (-61.7 to -59.5)	434 241 (261 045 to 685 917)	33 (20 to 50)	-18.1 (-21.0 to -15.9)	772 669 (597 840 to 1 024 033)	61 (48 to 79)	-45.4 (-50.2 to -40.5)
**Australasia**	4 269 (3 917 to 4 631)	14 (13 to 16)	-64.3 (-68.0 to -60.2)	13 761 (8 644 to 20 764)	42 (27 to 64)	5.7 (2.7 to 9.1)	18 030 (12 954 to 25 173)	57 (41 to 78)	-29.1 (-37.6 to -21.9)
Australia	3 539 (3 182 to 3 898)	14 (12 to 15)	-63.1 (-67.7 to -58.0)	11 060 (6 946 to 16 674)	40 (25 to 61)	9.6 (5.9 to 13.6)	14 599 (10 453 to 20 417)	54 (39 to 75)	-26.8 (-35.8 to -18.9)
New Zealand	730 (684 to 781)	17 (16 to 19)	-67.6 (-70.9 to -64.1)	2 701 (1 702 to 4 094)	54 (34 to 82)	-5.2 (-10.0 to 0.5)	3 431 (2 423 to 4 824)	72 (52 to 100)	-35.4 (-43.2 to -27.6)
**High-income Asia-Pacific**	50 987 (48 561 to 52 976)	21 (20 to 22)	-64.5 (-66.5 to -62.5)	112 660 (66 291 to 181 445)	45 (27 to 71)	-2.9 (-11.7 to 3.5)	163 647 (116 892 to 233 007)	66 (48 to 93)	-37.6 (-46.4 to -28.2)
Brunei	266 (208 to 309)	63 (51 to 73)	-47.4 (-56.7 to -37.5)	176 (110 to 267)	39 (24 to 59)	-39.6 (-48.5 to -31.2)	441 (354 to 539)	102 (83 to 123)	-44.7 (-52.3 to -37.4)
Japan	35 261 (33 829 to 36 675)	20 (19 to 21)	-48.9 (-51.5 to -46.3)	85 672 (49 391 to 140 743)	49 (28 to 79)	25.2 (23.4 to 27.5)	120 934 (84 305 to 176 462)	69 (48 to 99)	-12.0 (-21.9 to -3.1)
South Korea	15 087 (13 810 to 16 343)	25 (23 to 27)	-77.8 (-79.8 to -75.6)	24 118 (14 992 to 36 475)	37 (23 to 55)	-44.8 (-54.0 to -35.7)	39 205 (30 481 to 51 759)	62 (49 to 81)	-65.7 (-70.5 to -60.0)
Singapore	373 (341 to 409)	6 (6 to 7)	-60.9 (-65.1 to -56.5)	2 694 (1 677 to 4 069)	41 (26 to 63)	4.1 (-4.1 to 11.4)	3 067 (2 071 to 4 439)	48 (33 to 69)	-14.6 (-24.2 to -5.3)
**High-income North America**	150 747 (146 570 to 154 783)	39 (38 to 41)	-61.7 (-63.1 to -60.1)	132 879 (77 214 to 217 764)	30 (17 to 49)	-38.1 (-40.3 to -36.6)	283 626 (227 266 to 364 315)	69 (57 to 87)	-54.2 (-56.9 to -51.4)
Canada	11 270 (10 517 to 12 159)	30 (27 to 33)	-62.9 (-66.6 to -58.6)	15 538 (9 648 to 23 617)	35 (22 to 52)	-9.9 (-13.2 to -6.6)	26 808 (20 983 to 34 978)	64 (51 to 82)	-45.8 (-52.0 to -40.3)
Greenland	92 (76 to 116)	180 (145 to 238)	-64.5 (-74.1 to -37.4)	29 (20 to 43)	46 (31 to 67)	-63.6 (-67.9 to -59.4)	121 (102 to 147)	226 (186 to 282)	-64.3 (-72.3 to -44.9)
USA	139 383 (135 257 to 143 215)	40 (39 to 42)	-61.5 (-62.9 to -59.9)	117 309 (67 381 to 193 364)	29 (17 to 48)	-40.5 (-42.7 to -39.0)	256 693 (205 691 to 330 505)	70 (57 to 88)	-54.8 (-57.3 to -52.1)
**Southern Latin America**	37 213 (34 444 to 40 534)	56 (51 to 61)	-49.5 (-53.9 to -44.5)	35 842 (23 427 to 52 336)	52 (34 to 76)	-33.8 (-43.9 to -22.9)	73 054 (59 668 to 88 715)	108 (88 to 130)	-43.0 (-48.3 to -36.9)
Argentina	23 907 (21 635 to 26 635)	53 (48 to 59)	-42.9 (-49.2 to -35.9)	25 349 (16 838 to 36 592)	55 (37 to 79)	-26.9 (-37.3 to -15.7)	49 256 (39 813 to 60 381)	108 (88 to 132)	-35.8 (-42.1 to -29.0)
Chile	11 086 (9 870 to 12 443)	60 (53 to 67)	-62.3 (-66.8 to -56.8)	8 761 (5 509 to 13 434)	45 (29 to 69)	-49.2 (-59.0 to -37.9)	19 847 (16 324 to 24 413)	105 (87 to 129)	-57.6 (-62.5 to -51.5)
Uruguay	2 218 (1 985 to 2 457)	62 (56 to 69)	-27.8 (-37.2 to -17.6)	1 730 (1 119 to 2 594)	47 (30 to 70)	-30.7 (-41.2 to -19.6)	3 948 (3 282 to 4 859)	109 (91 to 134)	-29.0 (-35.9 to -21.7)
**Western Europe**	95 211 (91 768 to 98 270)	18 (17 to 18)	-62.9 (-64.6 to -61.2)	139 100 (86 755 to 212 419)	26 (16 to 40)	-1.8 (-3.3 to -0.2)	234 311 (180 928 to 307 672)	44 (34 to 57)	-40.9 (-46.9 to -34.6)
Andorra	17 (14 to 22)	21 (16 to 26)	-58.0 (-72.0 to -31.3)	27 (17 to 42)	27 (17 to 41)	-5.4 (-9.3 to -1.5)	45 (34 to 60)	48 (36 to 64)	-38.5 (-53.3 to -18.6)
Austria	1 693 (1 573 to 1 827)	15 (14 to 17)	-39.8 (-46.0 to -32.5)	2 941 (1 844 to 4 465)	27 (17 to 41)	12.3 (8.3 to 16.6)	4 634 (3 556 to 6 208)	42 (32 to 57)	-14.4 (-22.1 to -7.9)
Belgium	3 496 (3 251 to 3 781)	27 (24 to 29)	-55.2 (-60.6 to -49.0)	3 474 (2 160 to 5 290)	25 (16 to 38)	-10.1 (-13.5 to -6.5)	6 970 (5 669 to 8 793)	52 (42 to 64)	-40.7 (-46.7 to -34.8)
Cyprus	251 (213 to 299)	17 (14 to 22)	-59.2 (-66.1 to -45.4)	394 (248 to 597)	26 (17 to 40)	-16.0 (-24.1 to -8.8)	645 (496 to 855)	43 (33 to 58)	-40.6 (-49.6 to -30.5)
Denmark	1 951 (1 796 to 2 107)	27 (24 to 29)	-36.1 (-43.4 to -28.1)	1 904 (1 200 to 2 898)	28 (17 to 41)	10.2 (5.9 to 14.8)	3 855 (3 137 to 4 857)	54 (44 to 69)	-18.8 (-26.0 to -12.1)
Finland	1 991 (1 826 to 2 165)	30 (27 to 33)	-63.6 (-67.2 to -59.3)	2 131 (1 327 to 3 285)	31 (20 to 48)	-6.6 (-10.0 to -2.8)	4 122 (3 318 to 5 266)	61 (49 to 78)	-46.9 (-52.8 to -41.1)
France	20 196 (18 809 to 21 638)	24 (22 to 27)	-59.9 (-63.6 to -55.6)	22 369 (14 010 to 34 028)	28 (18 to 43)	-0.5 (-3.9 to 3.3)	42 564 (33 930 to 54 351)	53 (42 to 67)	-40.9 (-47.0 to -34.7)
Germany	15 460 (13 871 to 17 162)	15 (13 to 16)	-71.4 (-74.9 to -67.7)	28 119 (17 193 to 42 953)	27 (17 to 41)	0.4 (-3.6 to 4.4)	43 579 (33 098 to 58 153)	42 (32 to 56)	-47.0 (-53.9 to -39.6)
Greece	2 519 (2 352 to 2 709)	18 (17 to 20)	-51.4 (-56.9 to -45.8)	3 151 (1 987 to 4 773)	24 (15 to 36)	1.3 (-2.4 to 5.0)	5 669 (4 459 to 7 384)	42 (33 to 54)	-30.9 (-38.1 to -24.6)
Iceland	64 (59 to 69)	17 (15 to 19)	-55.4 (-60.6 to -49.4)	104 (65 to 158)	27 (17 to 41)	3.4 (-0.5 to 7.6)	169 (130 to 224)	44 (34 to 58)	-31.5 (-38.6 to -23.9)
Ireland	1 001 (904 to 1 093)	19 (17 to 21)	-72.7 (-76.2 to -68.8)	1 498 (942 to 2 253)	27 (17 to 40)	-9.3 (-12.8 to -5.2)	2 499 (1 932 to 3 292)	46 (36 to 60)	-53.6 (-60.0 to -47.2)
Israel	1 772 (1 636 to 1 914)	19 (17 to 20)	-54.0 (-59.1 to -48.5)	2 414 (1 518 to 3 654)	26 (16 to 39)	-15.5 (-20.8 to -10.7)	4 186 (3 311 to 5 440)	45 (35 to 58)	-37.3 (-43.9 to -31.3)
Italy	11 375 (10 619 to 12 167)	12 (11 to 12)	-62.3 (-65.7 to -58.3)	15 806 (9 887 to 24 117)	20 (13 to 31)	-7.6 (-10.7 to -4.1)	27 181 (21 217 to 35 558)	32 (24 to 42)	-39.5 (-46.1 to -33.8)
Luxembourg	88 (79 to 98)	12 (11 to 14)	-68.8 (-72.6 to -64.3)	195 (122 to 294)	28 (17 to 41)	12.6 (8.3 to 16.8)	283 (212 to 384)	40 (30 to 54)	-37.5 (-46.1 to -29.0)
Malta	55 (51 to 60)	11 (10 to 12)	-48.3 (-54.6 to -41.6)	183 (115 to 277)	34 (21 to 51)	27.1 (22.9 to 31.5)	239 (171 to 335)	45 (33 to 62)	-6.4 (-15.9 to 1.9)
Netherlands	2 165 (2 030 to 2 317)	10 (9 to 11)	-66.7 (-70.6 to -62.8)	3 470 (2 170 to 5 291)	17 (11 to 26)	-15.9 (-20.1 to -11.8)	5 635 (4 334 to 7 429)	26 (20 to 35)	-46.1 (-52.7 to -40.0)
Norway	1 763 (1 677 to 1 828)	28 (27 to 29)	-61.3 (-64.1 to -59.3)	2 182 (1 278 to 3 561)	34 (20 to 55)	-11.0 (-13.4 to -8.6)	3 945 (3 038 to 5 347)	62 (48 to 84)	-43.9 (-49.8 to -37.6)
Portugal	5 527 (5 348 to 5 695)	49 (48 to 51)	-28.4 (-34.6 to -21.8)	3 455 (2 157 to 5 227)	26 (16 to 39)	-32.1 (-39.0 to -25.4)	8 982 (7 683 to 10 764)	75 (65 to 88)	-29.7 (-34.7 to -25.3)
Spain	6 640 (6 148 to 7 187)	12 (11 to 13)	-73.4 (-76.4 to -70.4)	16 134 (10 058 to 24 543)	28 (17 to 42)	4.5 (1.1 to 8.2)	22 774 (16 672 to 31 418)	39 (29 to 54)	-44.0 (-52.0 to -35.7)
Sweden	3 031 (2 851 to 3 224)	23 (22 to 25)	-46.0 (-50.3 to -41.4)	4 264 (2 467 to 6 977)	34 (20 to 56)	3.6 (0.3 to 7.0)	7 295 (5 517 to 10 032)	57 (44 to 79)	-24.6 (-31.5 to -17.4)
Switzerland	930 (852 to 1 013)	10 (9 to 11)	-51.6 (-57.7 to -44.7)	2 787 (1 733 to 4 233)	27 (17 to 40)	18.2 (13.0 to 23.9)	3 716 (2 684 to 5 183)	36 (27 to 51)	-14.4 (-23.5 to -6.3)

Location	YLLs (95% UI)			YLDs (95% UI)			DALYs (95% UI)		
	2017 counts	2017 age-standardised rates per 100,000	Percentage change in age-standardised rates between 1990 and 2017	2017 counts	2017 age-standardised rates per 100,000	Percentage change in age-standardised rates between 1990 and 2017	2017 counts	2017 age-standardised rates per 100,000	Percentage change in age-standardised rates between 1990 and 2017
United Kingdom	13 127 (12 866 to 13 385)	17 (17 to 17)	-64.7 (-66.0 to -63.5)	21 955 (13 818 to 33 442)	28 (17 to 42)	1.1 (-0.8 to 3.1)	35 081 (26 801 to 46 636)	45 (34 to 59)	-40.9 (-47.6 to -34.2)
**Latin America and Caribbean**	**216 868 (205 860 to 230 545)**	**38 (36 to 41)**	**-68.9 (-71.1 to -65.0)**	**144 011 (96 811 to 209 798)**	**24 (16 to 35)**	**-42.0 (-47.6 to -36.9)**	**360 878 (310 260 to 427 855)**	**63 (54 to 74)**	**-62.1 (-65.5 to -57.9)**
**Andean Latin America**	**33 330 (27 475 to 44 311)**	**54 (45 to 71)**	**-68.7 (-75.9 to -50.4)**	**20 456 (14 235 to 28 025)**	**34 (24 to 47)**	**-51.5 (-58.9 to -42.0)**	**53 786 (44 963 to 66 662)**	**88 (74 to 109)**	**-63.7 (-70.4 to -50.1)**
Bolivia	8 805 (5 690 to 12 563)	75 (51 to 103)	-72.6 (-83.2 to -45.8)	4 934 (3 486 to 6 586)	45 (32 to 60)	-47.5 (-53.6 to -41.0)	13 739 (10 212 to 17 958)	120 (92 to 152)	-66.6 (-76.5 to -46.2)
Ecuador	8 745 (7 726 to 9 922)	54 (48 to 61)	-61.4 (-66.8 to -55.3)	5 390 (3 718 to 7 416)	33 (23 to 45)	-54.2 (-61.7 to -44.6)	14 135 (12 124 to 16 474)	87 (75 to 101)	-59.0 (-63.4 to -53.9)
Peru	15 779 (11 117 to 23 695)	47 (33 to 69)	-69.9 (-80.1 to -46.7)	10 133 (6 880 to 14 187)	31 (21 to 43)	-52.4 (-61.0 to -41.4)	25 912 (20 334 to 34 676)	78 (61 to 103)	-64.7 (-73.6 to -47.6)
**Caribbean**	**33 005 (22 275 to 43 982)**	**76 (50 to 103)**	**-60.9 (-71.2 to -46.1)**	**22 514 (15 716 to 30 348)**	**47 (33 to 64)**	**-24.1 (-30.0 to -17.1)**	**55 518 (42 411 to 68 092)**	**124 (93 to 154)**	**-52.0 (-62.6 to -38.2)**
Antigua and Barbuda	59 (53 to 67)	72 (63 to 85)	-56.1 (-63.3 to -47.3)	31 (21 to 45)	32 (22 to 47)	-44.8 (-52.0 to -36.8)	90 (77 to 107)	104 (88 to 124)	-53.2 (-59.4 to -46.1)
The Bahamas	343 (307 to 379)	95 (84 to 106)	-56.9 (-63.4 to -50.3)	141 (95 to 201)	35 (24 to 50)	-42.6 (-48.9 to -36.6)	483 (423 to 552)	130 (114 to 149)	-53.8 (-59.2 to -48.3)
Barbados	125 (114 to 137)	40 (36 to 44)	-60.5 (-65.4 to -55.0)	107 (70 to 157)	32 (21 to 46)	-24.2 (-33.8 to -13.6)	232 (194 to 283)	71 (60 to 86)	-49.9 (-55.5 to -43.6)
Belize	197 (176 to 223)	53 (47 to 59)	-62.3 (-68.1 to -55.6)	184 (127 to 248)	48 (33 to 64)	-19.8 (-26.7 to -12.5)	381 (321 to 448)	100 (85 to 118)	-49.6 (-55.6 to -43.2)
Bermuda	5 (5 to 6)	7 (6 to 8)	-71.4 (-75.6 to -66.7)	27 (17 to 40)	33 (21 to 50)	-1.5 (-15.5 to 12.8)	32 (22 to 46)	40 (28 to 57)	-31.6 (-43.3 to -18.6)
Cuba	2 405 (2 164 to 2 680)	19 (17 to 21)	-81.5 (-83.5 to -79.2)	4 091 (2 593 to 6 213)	31 (19 to 46)	-16.8 (-28.8 to -4.6)	6 495 (4 964 to 8 553)	49 (38 to 65)	-64.3 (-70.1 to -57.1)
Dominica	51 (45 to 58)	81 (70 to 96)	-37.1 (-47.2 to -24.1)	30 (21 to 42)	41 (28 to 56)	-21.1 (-28.9 to -13.2)	81 (70 to 93)	122 (105 to 142)	-32.6 (-40.1 to -23.2)
Dominican Republic	5 843 (4 207 to 7 333)	58 (41 to 72)	-58.3 (-68.6 to -36.5)	4 186 (2 860 to 5 802)	40 (27 to 55)	-46.3 (-54.9 to -34.9)	10 030 (8 087 to 11 995)	97 (79 to 116)	-54.1 (-62.6 to -40.4)
Grenada	80 (73 to 89)	70 (62 to 80)	-64.4 (-69.7 to -57.7)	54 (38 to 74)	47 (33 to 64)	-36.4 (-42.7 to -30.4)	135 (116 to 155)	116 (99 to 134)	-56.7 (-61.8 to -51.4)
Guyana	581 (505 to 662)	81 (71 to 92)	-56.8 (-63.2 to -49.3)	392 (272 to 516)	52 (36 to 69)	-27.8 (-34.2 to -21.4)	973 (832 to 1 131)	133 (114 to 155)	-48.7 (-54.2 to -42.5)
Haiti	18 826 (9 236 to 28 759)	153 (81 to 228)	-67.0 (-79.3 to -40.5)	8 952 (6 222 to 11 881)	77 (54 to 101)	-38.9 (-43.0 to -33.9)	27 778 (17 553 to 38 246)	230 (151 to 309)	-61.0 (-73.4 to -39.9)
Jamaica	1 004 (852 to 1 167)	37 (31 to 43)	-18.4 (-34.2 to -2.0)	1 250 (862 to 1 744)	43 (30 to 60)	-10.7 (-20.6 to 0.2)	2 255 (1 842 to 2 755)	80 (65 to 98)	-14.4 (-24.0 to -4.6)
Puerto Rico	864 (804 to 932)	20 (19 to 22)	-62.7 (-66.6 to -58.3)	1 267 (793 to 1 936)	29 (19 to 45)	4.0 (-7.0 to 14.8)	2 131 (1 664 to 2 774)	50 (39 to 64)	-39.9 (-48.0 to -30.7)
Saint Lucia	94 (86 to 104)	52 (47 to 58)	-66.8 (-71.1 to -62.0)	76 (51 to 107)	39 (27 to 56)	-39.5 (-46.9 to -32.0)	170 (144 to 202)	92 (78 to 108)	-58.9 (-63.4 to -54.4)
Saint Vincent and the Grenadines	78 (71 to 85)	69 (62 to 76)	-55.2 (-61.7 to -48.7)	54 (38 to 74)	45 (32 to 61)	-22.5 (-29.3 to -15.9)	132 (115 to 153)	114 (99 to 131)	-46.2 (-51.8 to -40.0)
Suriname	389 (348 to 434)	71 (63 to 79)	-64.2 (-69.4 to -57.9)	288 (204 to 386)	48 (34 to 65)	-35.6 (-41.9 to -29.1)	676 (579 to 787)	119 (103 to 139)	-56.3 (-61.3 to -50.9)
Trinidad and Tobago	828 (692 to 978)	65 (54 to 78)	-43.0 (-53.8 to -30.7)	539 (370 to 759)	35 (24 to 50)	-30.4 (-38.8 to -20.9)	1 366 (1 134 to 1 625)	100 (83 to 119)	-39.1 (-47.7 to -29.8)
Virgin Islands	45 (39 to 51)	37 (32 to 42)	-57.7 (-64.2 to -50.8)	36 (23 to 54)	30 (19 to 45)	-33.1 (-45.4 to -20.0)	81 (66 to 100)	66 (55 to 81)	-49.4 (-56.5 to -41.7)
**Central Latin America**	**83 328 (79 569 to 87 179)**	**33 (32 to 35)**	**-67.8 (-69.9 to -65.8)**	**62 257 (41 143 to 91 671)**	**24 (16 to 36)**	**-44.3 (-50.6 to -38.0)**	**145 584 (123 766 to 175 650)**	**58 (49 to 69)**	**-60.8 (-64.2 to -57.1)**
Colombia	9 330 (8 086 to 10 753)	19 (17 to 22)	-81.4 (-84.1 to -78.2)	12 410 (8 190 to 18 138)	24 (16 to 35)	-59.0 (-66.1 to -50.5)	21 741 (17 077 to 27 465)	43 (34 to 55)	-73.3 (-77.4 to -68.9)
Costa Rica	1 028 (916 to 1 123)	22 (20 to 24)	-41.1 (-48.5 to -34.0)	1 030 (653 to 1 570)	21 (13 to 32)	-32.5 (-43.7 to -20.4)	2 058 (1 660 to 2 602)	44 (35 to 55)	-37.2 (-44.0 to -30.1)
El Salvador	1 849 (1 353 to 2 737)	31 (23 to 46)	-71.2 (-81.8 to -48.9)	1 586 (1 070 to 2 269)	26 (17 to 37)	-61.2 (-67.9 to -52.6)	3 435 (2 683 to 4 423)	57 (44 to 73)	-67.3 (-75.7 to -53.7)
Guatemala	12 367 (10 837 to 14 289)	75 (67 to 86)	-49.4 (-56.1 to -41.7)	6 574 (4 563 to 8 959)	40 (28 to 55)	-49.0 (-54.6 to -42.0)	18 941 (16 188 to 21 699)	116 (100 to 132)	-49.3 (-54.0 to -43.7)
Honduras	2 090 (1 589 to 3 019)	23 (18 to 31)	-64.4 (-72.8 to -48.0)	4 684 (3 241 to 6 294)	51 (36 to 68)	-22.7 (-30.5 to -13.6)	6 774 (5 221 to 8 652)	74 (57 to 93)	-43.2 (-50.9 to -33.2)
Mexico	45 070 (41 842 to 46 596)	37 (34 to 38)	-68.0 (-70.8 to -66.5)	24 266 (14 430 to 39 212)	19 (11 to 31)	-39.7 (-43.6 to -36.4)	69 336 (59 036 to 84 267)	56 (48 to 68)	-61.8 (-65.1 to -58.3)
Nicaragua	1 080 (804 to 1 660)	18 (13 to 27)	-77.6 (-85.4 to -52.4)	1 940 (1 334 to 2 712)	31 (22 to 44)	-41.9 (-50.2 to -32.0)	3 020 (2 285 to 3 919)	49 (37 to 63)	-63.1 (-72.1 to -45.5)
Panama	927 (811 to 1 071)	24 (21 to 28)	-46.3 (-54.3 to -36.4)	962 (632 to 1 401)	24 (16 to 35)	-38.1 (-48.5 to -26.1)	1 889 (1 546 to 2 333)	48 (40 to 59)	-42.5 (-49.2 to -34.6)
Venezuela	9 587 (8 231 to 11 173)	31 (27 to 36)	-54.5 (-61.2 to -46.9)	8 803 (5 784 to 12 811)	28 (19 to 41)	-39.2 (-48.7 to -27.5)	18 391 (15 215 to 22 831)	60 (49 to 74)	-48.3 (-54.2 to -41.5)
**Tropical Latin America**	**67 205 (64 762 to 69 761)**	**32 (30 to 33)**	**-73.3 (-75.1 to -71.5)**	**38 784 (22 821 to 61 296)**	**17 (10 to 26)**	**-41.5 (-44.5 to -39.4)**	**105 989 (90 190 to 128 110)**	**48 (42 to 58)**	**-67.2 (-70.0 to -64.2)**
Brazil	64 784 (62 492 to 67 138)	32 (30 to 33)	-73.7 (-75.5 to -71.9)	36 748 (21 466 to 58 734)	16 (9 to 26)	-41.8 (-44.8 to -39.7)	101 532 (86 335 to 123 197)	48 (41 to 57)	-67.7 (-70.6 to -64.8)
Paraguay	2 421 (1 903 to 3 478)	36 (29 to 51)	-52.8 (-63.0 to -36.5)	2 036 (1 423 to 2 746)	31 (21 to 41)	-42.3 (-48.9 to -34.3)	4 457 (3 611 to 5 674)	67 (54 to 84)	-48.5 (-56.0 to -38.3)
**North Africa and Middle East**	**566 160 (395 766 to 682 033)**	**92 (65 to 111)**	**-64.3 (-70.5 to -52.9)**	**268 833 (186 677 to 372 830)**	**46 (32 to 65)**	**-49.6 (-56.7 to -41.0)**	**834 993 (628 426 to 1 007 453)**	**139 (105 to 168)**	**-60.4 (-66.8 to -51.5)**
**North Africa and Middle East**	**566 160 (395 766 to 682 033)**	**92 (65 to 111)**	**-64.3 (-70.5 to -52.9)**	**268 833 (186 677 to 372 830)**	**46 (32 to 65)**	**-49.6 (-56.7 to -41.0)**	**834 993 (628 426 to 1 007 453)**	**139 (105 to 168)**	**-60.4 (-66.8 to -51.5)**
Afghanistan	30 038 (23 116 to 39 043)	90 (72 to 113)	-46.8 (-66.5 to 52.6)	23 301 (16 420 to 30 597)	85 (60 to 110)	9.9 (3.6 to 16.5)	53 340 (43 031 to 65 108)	175 (143 to 209)	-29.1 (-49.8 to 31.8)
Algeria	37 234 (18 698 to 59 372)	89 (45 to 143)	-61.3 (-69.3 to -47.6)	19 066 (13 171 to 26 437)	47 (32 to 65)	-54.1 (-61.1 to -46.1)	56 300 (35 875 to 80 229)	136 (87 to 194)	-59.1 (-65.9 to -49.2)
Bahrain	460 (353 to 717)	35 (25 to 49)	-32.3 (-61.8 to 14.3)	672 (434 to 988)	40 (26 to 59)	-34.2 (-47.2 to -18.9)	1 132 (860 to 1 550)	75 (57 to 99)	-33.3 (-52.6 to -14.4)
Egypt	112 863 (76 758 to 151 644)	113 (78 to 149)	-51.1 (-62.8 to -33.8)	43 833 (30 953 to 59 729)	49 (34 to 67)	-53.8 (-60.9 to -45.5)	156 697 (116 906 to 198 848)	162 (123 to 201)	-52.0 (-60.8 to -40.4)
Iran	94 351 (80 346 to 106 671)	116 (99 to 132)	-73.3 (-79.5 to -57.2)	33 256 (21 471 to 48 584)	38 (25 to 56)	-66.7 (-73.3 to -58.9)	127 607 (107 993 to 149 979)	154 (130 to 182)	-71.9 (-77.8 to -59.7)
Iraq	26 763 (21 189 to 31 985)	56 (46 to 67)	-73.0 (-81.7 to -36.0)	24 850 (17 626 to 32 674)	66 (47 to 87)	-19.4 (-24.3 to -14.4)	51 613 (42 718 to 61 351)	122 (100 to 146)	-57.8 (-69.3 to -27.9)
Jordan	7 521 (5 881 to 9 429)	69 (56 to 86)	-65.5 (-73.2 to -50.6)	3 400 (2 227 to 4 932)	36 (23 to 52)	-53.1 (-61.5 to -43.7)	10 921 (8 709 to 13 422)	105 (85 to 128)	-62.1 (-69.3 to -50.7)
Kuwait	1 515 (1 363 to 1 673)	35 (32 to 39)	-46.3 (-52.9 to -39.3)	1 725 (1 079 to 2 594)	37 (23 to 56)	-11.3 (-21.7 to -2.2)	3 240 (2 612 to 4 122)	72 (59 to 91)	-32.7 (-40.1 to -24.7)
Lebanon	5 463 (2 613 to 10 237)	63 (30 to 115)	-74.0 (-80.5 to -62.7)	2 822 (1 773 to 4 283)	35 (22 to 54)	-52.7 (-62.7 to -41.7)	8 285 (5 022 to 13 005)	98 (60 to 151)	-69.0 (-74.0 to -60.3)
Libya	6 711 (2 115 to 10 731)	95 (30 to 151)	-48.9 (-68.7 to -23.5)	2 368 (1 558 to 3 469)	34 (22 to 51)	-53.4 (-61.8 to -43.7)	9 079 (4 493 to 13 256)	129 (65 to 188)	-50.2 (-64.1 to -35.0)
Morocco	41 300 (20 778 to 59 951)	117 (59 to 170)	-59.8 (-69.3 to -44.9)	17 932 (12 570 to 24 232)	49 (34 to 66)	-53.2 (-59.3 to -46.1)	59 233 (37 038 to 79 804)	166 (103 to 224)	-58.0 (-66.3 to -47.8)
Palestine	1 359 (913 to 2 402)	28 (20 to 47)	-69.7 (-81.5 to -44.0)	2 688 (1 873 to 3 614)	65 (45 to 88)	12.7 (3.0 to 21.5)	4 047 (2 986 to 5 329)	93 (69 to 122)	-38.2 (-53.2 to -18.9)
Oman	880 (622 to 1 353)	21 (16 to 29)	-63.4 (-73.2 to -32.3)	1 490 (940 to 2 226)	33 (20 to 49)	-32.4 (-47.3 to -15.9)	2 370 (1 708 to 3 237)	54 (41 to 72)	-49.3 (-60.7 to -31.2)
Qatar	1 041 (688 to 1 525)	38 (26 to 53)	-56.2 (-72.8 to -27.5)	1 034 (640 to 1 554)	35 (22 to 52)	-34.4 (-46.7 to -21.2)	2 076 (1 532 to 2 843)	72 (55 to 96)	-47.9 (-61.9 to -30.4)
Saudi Arabia	34 677 (26 228 to 46 261)	97 (76 to 128)	-65.1 (-77.9 to -34.1)	11 553 (7 390 to 17 477)	33 (21 to 50)	-64.8 (-72.8 to -55.6)	46 229 (36 634 to 58 414)	130 (104 to 163)	-65.0 (-75.1 to -44.4)
Sudan	71 376 (29 494 to 115 803)	158 (68 to 248)	-67.8 (-78.0 to -37.0)	11 677 (7 256 to 18 347)	38 (23 to 60)	-28.7 (-32.6 to -25.2)	83 053 (40 881 to 128 000)	196 (105 to 287)	-63.9 (-75.4 to -35.3)
Syria	6 716 (5 124 to 8 886)	39 (30 to 52)	-71.4 (-81.4 to -55.0)	7 337 (4 936 to 10 453)	43 (29 to 62)	-35.9 (-47.1 to -22.5)	14 054 (10 981 to 17 653)	83 (65 to 105)	-59.7 (-70.3 to -46.7)
Tunisia	6 788 (3 139 to 12 158)	60 (27 to 109)	-68.1 (-77.2 to -53.1)	4 816 (3 136 to 7 062)	39 (25 to 56)	-55.6 (-64.0 to -45.7)	11 604 (7 276 to 17 574)	98 (61 to 152)	-64.1 (-72.5 to -53.3)
Turkey	18 770 (13 701 to 22 925)	25 (18 to 31)	-66.2 (-79.7 to -43.2)	32 746 (20 428 to 49 181)	37 (23 to 56)	-48.6 (-62.3 to -32.0)	51 516 (38 159 to 69 206)	62 (46 to 83)	-57.5 (-69.7 to -43.1)
United Arab Emirates	6 785 (4 818 to 9 272)	74 (55 to 94)	-48.6 (-64.7 to -25.6)	4 442 (2 904 to 6 472)	37 (24 to 54)	-48.2 (-57.7 to -37.3)	11 227 (8 594 to 14 375)	110 (87 to 135)	-48.5 (-60.6 to -33.5)
Yemen	53 017 (24 238 to 86 788)	166 (75 to 266)	-63.2 (-76.6 to -11.1)	17 573 (12 306 to 23 106)	70 (50 to 91)	-48.0 (-52.7 to -42.6)	70 590 (41 349 to 104 441)	235 (143 to 341)	-59.7 (-72.5 to -29.1)
**South Asia**	**1 557 948 (1 106 206 to 1 922 240)**	**88 (63 to 107)**	**-50.3 (-57.6 to -32.2)**	**902 076 (645 769 to 1 176 952)**	**52 (37 to 68)**	**-26.6 (-32.1 to -19.5)**	**2 460 024 (1 929 025 to 2 957 623)**	**140 (110 to 167)**	**-43.5 (-49.9 to -30.0)**
**South Asia**	**1 557 948 (1 106 206 to 1 922 240)**	**88 (63 to 107)**	**-50.3 (-57.6 to -32.2)**	**902 076 (645 769 to 1 176 952)**	**52 (37 to 68)**	**-26.6 (-32.1 to -19.5)**	**2 460 024 (1 929 025 to 2 957 623)**	**140 (110 to 167)**	**-43.5 (-49.9 to -30.0)**
Bangladesh	76 146 (55 005 to 114 686)	50 (37 to 74)	-74.4 (-82.1 to -53.8)	78 981 (56 160 to 103 311)	51 (36 to 67)	-43.3 (-50.0 to -34.7)	155 127 (119 474 to 201 141)	102 (79 to 130)	-64.6 (-71.8 to -49.2)
Bhutan	482 (291 to 744)	50 (32 to 76)	-68.0 (-79.5 to -45.9)	489 (346 to 643)	52 (37 to 68)	-41.3 (-48.2 to -32.9)	971 (711 to 1 281)	102 (77 to 134)	-58.4 (-67.9 to -44.1)
India	1 302 830 (876 315 to 1 605 973)	95 (64 to 115)	-47.7 (-56.1 to -27.6)	686 418 (488 361 to 908 135)	50 (36 to 66)	-27.0 (-32.7 to -19.7)	1 989 247 (1 510 953 to 2 397 971)	145 (111 to 174)	-42.0 (-49.0 to -27.0)
Nepal	17 258 (11 196 to 26 182)	59 (39 to 89)	-60.5 (-73.6 to -35.1)	15 810 (11 126 to 20 647)	56 (40 to 73)	-25.7 (-32.2 to -19.1)	33 068 (25 257 to 43 321)	116 (88 to 149)	-48.8 (-59.9 to -32.0)
Pakistan	161 232 (114 041 to 247 961)	70 (50 to 106)	-42.4 (-59.4 to -16.2)	120 378 (85 526 to 157 435)	63 (45 to 83)	-5.2 (-12.2 to 2.4)	281 610 (222 948 to 378 894)	133 (106 to 176)	-29.2 (-44.4 to -11.9)
**Southeast Asia, East Asia, and Oceania**	**618 455 (526 353 to 693 539)**	**31 (26 to 34)**	**-66.5 (-70.8 to -52.2)**	**518 157 (319 297 to 791 689)**	**21 (13 to 31)**	**-23.8 (-38.2 to -9.2)**	**1 136 611 (919 686 to 1 423 698)**	**51 (42 to 63)**	**-56.8 (-63.6 to -43.0)**
**East Asia**	**337 686 (276 482 to 384 719)**	**24 (20 to 28)**	**-74.3 (-78.0 to -59.3)**	**451 067 (268 412 to 700 327)**	**25 (15 to 39)**	**-18.4 (-35.4 to -1.4)**	**788 753 (601 788 to 1 056 217)**	**49 (38 to 63)**	**-60.4 (-68.1 to -44.7)**
China	314 464 (254 116 to 358 170)	23 (19 to 27)	-74.8 (-78.6 to -59.2)	430 651 (254 314 to 671 143)	25 (15 to 39)	-18.0 (-35.6 to -0.7)	745 115 (565 919 to 1 003 602)	48 (38 to 63)	-60.7 (-68.7 to -44.4)
North Korea	11 886 (7 389 to 17 419)	52 (33 to 77)	-22.1 (-47.6 to 6.7)	8 367 (5 923 to 11 285)	29 (20 to 39)	-1.9 (-9.0 to 4.9)	20 253 (14 784 to 26 087)	81 (59 to 106)	-16.0 (-34.3 to 4.2)
Taiwan (Province of China)	5 897 (5 495 to 6 322)	26 (24 to 28)	-80.4 (-82.2 to -78.3)	4 782 (2 948 to 7 258)	16 (10 to 25)	-51.9 (-60.1 to -43.9)	10 679 (8 827 to 13 061)	42 (36 to 50)	-74.5 (-77.5 to -71.0)
**Oceania**	**20 234 (11 974 to 27 414)**	**151 (92 to 202)**	**-22.5 (-37.6 to -1.9)**	**5 575 (3 956 to 7 308)**	**47 (33 to 61)**	**-5.1 (-10.2 to -0.1)**	**25 808 (17 237 to 33 488)**	**198 (137 to 252)**	**-19.0 (-32.0 to -2.7)**
American Samoa	25 (20 to 30)	47 (39 to 57)	-35.7 (-52.3 to -5.4)	17 (12 to 23)	31 (22 to 43)	-16.0 (-25.4 to -5.9)	41 (35 to 49)	79 (66 to 94)	-29.1 (-41.9 to -9.9)
Federated States of Micronesia	75 (43 to 107)	74 (43 to 106)	-36.3 (-60.4 to -10.6)	43 (31 to 57)	43 (30 to 56)	-12.2 (-19.9 to -4.7)	118 (86 to 151)	117 (85 to 150)	-29.3 (-46.9 to -11.0)
Fiji	1 632 (1 338 to 1 959)	182 (150 to 218)	-11.7 (-37.8 to 14.8)	460 (329 to 606)	50 (36 to 66)	-14.7 (-20.8 to -9.1)	2 092 (1 768 to 2 452)	232 (196 to 271)	-12.3 (-33.1 to 6.8)
Guam	47 (40 to 59)	29 (24 to 36)	-24.6 (-42.1 to 8.5)	43 (28 to 63)	25 (16 to 36)	3.1 (-6.9 to 12.2)	91 (73 to 113)	54 (43 to 66)	-13.9 (-27.7 to 6.1)
Kiribati	50 (37 to 67)	40 (31 to 53)	-20.4 (-42.6 to 18.8)	64 (45 to 83)	57 (41 to 74)	35.9 (25.5 to 46.5)	114 (92 to 138)	97 (79 to 116)	5.2 (-13.5 to 27.9)

Location	YLLs (95% UI)			YLDs (95% UI)			DALYs (95% UI)		
	2017 counts	2017 age-standardised rates per 100,000	Percentage change in age-standardised rates between 1990 and 2017	2017 counts	2017 age-standardised rates per 100,000	Percentage change in age-standardised rates between 1990 and 2017	2017 counts	2017 age-standardised rates per 100,000	Percentage change in age-standardised rates between 1990 and 2017
Marshall Islands	54 (34 to 73)	97 (61 to 131)	-19.5 (-46.4 to 12.3)	22 (16 to 29)	40 (29 to 52)	-3.4 (-10.9 to 4.7)	76 (55 to 95)	137 (101 to 171)	-15.4 (-36.3 to 8.1)
Northern Mariana Islands	16 (14 to 20)	39 (33 to 48)	-41.8 (-57.1 to -21.5)	11 (7 to 17)	21 (14 to 32)	-21.8 (-32.9 to -11.4)	27 (22 to 33)	61 (49 to 73)	-36.0 (-49.1 to -21.3)
Papua New Guinea	15 945 (8 199 to 22 599)	160 (85 to 223)	-26.3 (-42.7 to -0.3)	4 039 (2 871 to 5 279)	46 (33 to 60)	-5.5 (-12.3 to 1.2)	19 984 (12 023 to 26 844)	205 (129 to 271)	-22.5 (-37.0 to -1.4)
Samoa	119 (76 to 190)	61 (40 to 98)	-28.0 (-47.7 to -4.0)	79 (56 to 102)	44 (32 to 57)	-5.3 (-12.4 to 2.1)	198 (146 to 278)	106 (79 to 147)	-20.0 (-34.9 to -4.6)
Solomon Islands	705 (513 to 947)	109 (81 to 149)	-27.4 (-46.5 to -0.8)	297 (208 to 388)	51 (36 to 66)	4.5 (-2.4 to 11.0)	1 002 (800 to 1 261)	160 (129 to 201)	-19.6 (-35.8 to 0.4)
Tonga	156 (119 to 198)	153 (117 to 194)	-18.2 (-37.0 to 9.8)	49 (35 to 64)	51 (37 to 67)	-17.7 (-24.6 to -10.1)	204 (166 to 250)	204 (167 to 248)	-18.1 (-32.6 to 3.5)
Vanuatu	295 (183 to 427)	101 (63 to 146)	-16.7 (-43.0 to 22.5)	143 (101 to 185)	55 (39 to 71)	7.1 (0.3 to 13.8)	439 (320 to 574)	155 (115 to 201)	-9.6 (-30.3 to 16.9)
**Southeast Asia**	**260 535 (227 102 to 285 189)**	**42 (36 to 45)**	**-54.2 (-61.4 to -38.6)**	**61 515 (42 349 to 86 328)**	**9 (6 to 13)**	**-44.0 (-49.3 to -38.6)**	**322 050 (284 855 to 355 477)**	**51 (44 to 56)**	**-52.7 (-59.4 to -39.8)**
Cambodia	11 857 (8 343 to 16 413)	75 (54 to 103)	-67.8 (-78.4 to -41.0)	2 871 (2 029 to 3 790)	18 (13 to 24)	-52.2 (-56.3 to -47.2)	14 728 (10 958 to 19 371)	94 (71 to 122)	-65.6 (-75.4 to -42.6)
Indonesia	84 060 (65 473 to 95 303)	35 (27 to 41)	-63.0 (-71.7 to -38.1)	13 008 (7 976 to 20 343)	5 (3 to 8)	-50.8 (-53.4 to -47.5)	97 067 (77 151 to 111 352)	40 (32 to 47)	-61.8 (-70.3 to -40.1)
Laos	7 792 (4 794 to 10 818)	109 (69 to 149)	-68.2 (-78.6 to -35.1)	1 313 (917 to 1 743)	20 (14 to 26)	-58.1 (-62.3 to -54.1)	9 105 (6 034 to 12 232)	128 (87 to 169)	-67.0 (-76.8 to -40.8)
Malaysia	13 931 (11 829 to 16 306)	47 (40 to 54)	-46.9 (-55.4 to -36.6)	2 623 (1 728 to 3 810)	8 (6 to 12)	-60.3 (-67.8 to -51.0)	16 554 (14 274 to 19 096)	56 (48 to 63)	-49.5 (-56.3 to -41.6)
Maldives	166 (135 to 247)	41 (34 to 56)	-82.3 (-87.9 to -60.1)	36 (24 to 53)	8 (5 to 11)	-79.9 (-84.5 to -74.0)	202 (167 to 289)	48 (41 to 65)	-81.9 (-87.2 to -65.6)
Mauritius	1 070 (975 to 1 177)	83 (76 to 92)	-71.4 (-74.5 to -68.0)	166 (108 to 240)	11 (7 to 16)	-71.5 (-75.7 to -66.9)	1 236 (1 122 to 1 360)	94 (86 to 104)	-71.4 (-74.2 to -68.3)
Myanmar	15 501 (11 821 to 21 696)	31 (24 to 43)	-61.3 (-74.7 to -31.9)	7 641 (5 360 to 10 191)	14 (10 to 19)	-24.6 (-32.2 to -16.2)	23 142 (18 490 to 29 823)	45 (37 to 58)	-54.5 (-67.0 to -29.2)
Philippines	59 364 (51 782 to 68 534)	58 (51 to 67)	-34.7 (-44.1 to -23.3)	11 021 (7 689 to 14 791)	11 (8 to 15)	-38.2 (-44.8 to -31.2)	70 385 (61 948 to 80 051)	69 (61 to 78)	-35.3 (-43.1 to -25.6)
Sri Lanka	14 622 (11 749 to 19 253)	67 (54 to 89)	-52.5 (-63.7 to -36.6)	2 177 (1 392 to 3 253)	9 (6 to 14)	-63.7 (-70.6 to -55.2)	16 799 (13 905 to 21 544)	77 (63 to 99)	-54.2 (-64.1 to -40.9)
Seychelles	142 (119 to 170)	138 (116 to 164)	-23.2 (-45.9 to 5.2)	16 (11 to 24)	15 (10 to 21)	-52.6 (-59.8 to -43.9)	158 (135 to 187)	152 (131 to 181)	-27.5 (-46.9 to -5.0)
Thailand	29 469 (20 237 to 35 512)	41 (29 to 49)	-35.2 (-61.7 to 0.1)	7 357 (4 714 to 10 819)	9 (6 to 13)	-50.3 (-60.7 to -38.3)	36 826 (27 366 to 43 850)	50 (38 to 59)	-38.5 (-60.2 to -13.4)
Timor-Leste	1 004 (531 to 1 362)	75 (42 to 102)	-71.2 (-84.6 to -45.3)	184 (128 to 244)	16 (12 to 21)	-59.3 (-63.3 to -54.2)	1 189 (717 to 1 541)	91 (58 to 119)	-69.6 (-81.7 to -48.2)
Vietnam	21 213 (17 215 to 26 140)	22 (18 to 28)	-52.4 (-65.6 to -28.8)	13 021 (8 874 to 18 053)	13 (9 to 18)	-33.9 (-44.5 to -21.4)	34 233 (28 556 to 41 152)	35 (29 to 42)	-47.0 (-58.7 to -29.9)
**Sub-Saharan Africa**	**1 481 063 (1 167 082 to 1 715 145)**	**139 (112 to 158)**	**-46.0 (-55.7 to -26.1)**	**627 159 (444 725 to 819 109)**	**74 (53 to 96)**	**-22.4 (-26.2 to -18.3)**	**2 108 222 (1 774 010 to 2 393 778)**	**214 (181 to 245)**	**-39.6 (-48.0 to -25.1)**
**Central sub-Saharan Africa**	**194 070 (115 177 to 274 367)**	**146 (91 to 193)**	**-46.6 (-61.3 to -12.8)**	**76 775 (54 502 to 100 670)**	**76 (54 to 98)**	**-15.5 (-20.2 to -10.6)**	**270 845 (193 162 to 356 411)**	**222 (166 to 273)**	**-38.9 (-52.1 to -14.1)**
Angola	44 371 (32 204 to 60 548)	144 (113 to 181)	-64.2 (-76.9 to -17.1)	16 002 (11 243 to 21 278)	72 (51 to 94)	-33.9 (-38.8 to -28.4)	60 373 (47 024 to 77 423)	216 (178 to 257)	-57.7 (-70.2 to -23.5)
Central African Republic	10 918 (4 418 to 18 545)	233 (101 to 374)	-26.2 (-51.3 to 18.0)	3 470 (2 467 to 4 589)	80 (57 to 105)	0.1 (-5.7 to 6.7)	14 388 (7 979 to 22 251)	313 (186 to 461)	-20.8 (-39.6 to 12.6)
Congo (Brazzaville)	5 981 (3 781 to 8 578)	129 (81 to 176)	-52.1 (-65.2 to -22.5)	3 123 (2 196 to 4 097)	69 (49 to 89)	-23.9 (-29.5 to -18.2)	9 104 (6 892 to 11 888)	198 (150 to 251)	-45.1 (-56.5 to -23.4)
DR Congo	130 063 (62 650 to 197 310)	145 (77 to 201)	-38.8 (-57.6 to -3.8)	52 598 (37 334 to 69 574)	78 (56 to 101)	-7.9 (-13.8 to -1.7)	182 661 (118 068 to 253 909)	223 (155 to 286)	-30.7 (-46.8 to -4.7)
Equatorial Guinea	1 112 (661 to 1 718)	93 (58 to 141)	-72.9 (-84.7 to -35.6)	505 (358 to 676)	47 (34 to 63)	-53.2 (-59.9 to -44.4)	1 618 (1 140 to 2 239)	140 (103 to 189)	-68.4 (-79.0 to -42.0)
Gabon	1 625 (1 049 to 2 601)	103 (67 to 160)	-52.1 (-65.9 to -16.7)	1 077 (765 to 1 392)	68 (48 to 88)	-29.6 (-34.6 to -24.6)	2 702 (2 072 to 3 737)	171 (132 to 232)	-45.2 (-56.5 to -21.4)
**Eastern sub-Saharan Africa**	**528 920 (433 544 to 651 281)**	**139 (116 to 167)**	**-49.9 (-61.1 to -22.3)**	**282 466 (200 912 to 368 955)**	**89 (64 to 114)**	**-19.2 (-23.4 to -14.8)**	**811 386 (687 123 to 970 780)**	**227 (194 to 269)**	**-41.1 (-51.9 to -20.9)**
Burundi	19 009 (12 955 to 26 250)	178 (125 to 234)	-47.4 (-62.6 to -16.9)	9 208 (6 466 to 12 213)	102 (72 to 133)	-23.7 (-28.5 to -18.5)	28 217 (21 634 to 36 096)	280 (219 to 347)	-40.7 (-53.7 to -18.8)
Comoros	817 (584 to 1 190)	126 (93 to 176)	-54.8 (-68.6 to -28.8)	668 (471 to 870)	101 (71 to 131)	-34.8 (-39.0 to -30.2)	1 484 (1 173 to 1 920)	227 (181 to 286)	-47.7 (-58.1 to -31.7)
Djibouti	1 268 (780 to 2 207)	125 (79 to 209)	-53.1 (-69.9 to -21.5)	954 (677 to 1 247)	91 (65 to 117)	-34.7 (-39.5 to -29.8)	2 222 (1 640 to 3 247)	215 (163 to 301)	-46.8 (-59.5 to -27.2)
Eritrea	9 668 (6 849 to 12 660)	186 (133 to 232)	-47.2 (-62.9 to -7.7)	5 132 (3 619 to 6 739)	98 (70 to 127)	-25.5 (-30.3 to -20.2)	14 800 (11 738 to 18 122)	284 (227 to 338)	-41.3 (-55.2 to -14.6)
Ethiopia	109 554 (86 979 to 145 219)	112 (91 to 148)	-62.9 (-74.5 to -30.7)	75 241 (52 714 to 98 895)	92 (66 to 120)	-23.0 (-27.9 to -17.9)	184 795 (150 363 to 232 317)	204 (168 to 252)	-51.5 (-63.4 to -27.9)
Kenya	46 456 (37 647 to 66 497)	117 (97 to 162)	-40.2 (-50.6 to -15.1)	17 479 (10 885 to 27 007)	49 (30 to 76)	2.2 (-0.9 to 5.2)	63 935 (51 821 to 86 913)	166 (136 to 220)	-31.9 (-43.8 to -11.0)
Madagascar	38 014 (27 717 to 51 194)	151 (113 to 197)	-46.1 (-60.0 to -23.1)	22 302 (15 847 to 28 993)	103 (73 to 134)	-26.3 (-31.3 to -21.4)	60 317 (48 067 to 75 602)	254 (207 to 312)	-39.5 (-50.3 to -24.6)
Malawi	21 623 (16 865 to 28 808)	130 (106 to 164)	-52.1 (-70.0 to 16.2)	11 905 (8 369 to 15 737)	83 (59 to 108)	-15.0 (-21.0 to -8.8)	33 528 (27 121 to 41 288)	213 (177 to 255)	-42.3 (-59.8 to 3.3)
Mozambique	50 663 (38 974 to 69 851)	182 (147 to 229)	-52.1 (-65.8 to -18.5)	20 959 (14 851 to 27 459)	84 (60 to 108)	-39.1 (-44.3 to -33.1)	71 622 (58 252 to 91 537)	266 (222 to 319)	-48.6 (-60.1 to -26.2)
Rwanda	16 575 (11 533 to 26 838)	139 (101 to 214)	-57.5 (-72.4 to -21.7)	9 457 (6 677 to 12 380)	89 (64 to 115)	-35.4 (-40.5 to -30.3)	26 032 (19 814 to 36 549)	228 (177 to 307)	-50.9 (-63.7 to -26.5)
Somalia	31 496 (20 769 to 47 413)	191 (131 to 284)	-40.1 (-64.2 to 27.8)	16 934 (12 032 to 22 445)	122 (88 to 158)	-7.2 (-13.5 to -0.7)	48 430 (36 167 to 65 477)	314 (242 to 413)	-30.5 (-52.1 to 11.9)
South Sudan	26 208 (14 950 to 40 120)	243 (153 to 365)	-24.1 (-48.2 to 33.8)	9 456 (6 702 to 12 310)	118 (84 to 152)	-5.1 (-10.8 to 0.0)	35 664 (24 608 to 49 809)	360 (263 to 484)	-18.8 (-39.4 to 17.5)
Tanzania	86 248 (64 646 to 125 784)	142 (114 to 189)	-33.6 (-53.5 to 17.7)	41 750 (29 182 to 55 294)	94 (67 to 123)	-9.6 (-15.7 to -3.3)	127 998 (102 231 to 167 101)	236 (196 to 289)	-25.8 (-41.7 to 6.2)
Uganda	47 248 (34 741 to 67 032)	123 (92 to 163)	-46.3 (-60.8 to -14.8)	27 535 (19 258 to 36 488)	91 (65 to 119)	2.6 (-4.5 to 9.8)	74 783 (59 865 to 97 313)	214 (175 to 267)	-32.6 (-46.4 to -8.3)
Zambia	23 741 (18 243 to 31 566)	142 (115 to 177)	-53.7 (-67.9 to -12.3)	13 308 (9 346 to 17 547)	92 (65 to 119)	-10.8 (-17.1 to -4.5)	37 049 (29 925 to 46 211)	233 (195 to 278)	-42.9 (-56.9 to -11.3)
**Southern sub-Saharan Africa**	**130 012 (103 189 to 160 407)**	**171 (137 to 208)**	**-46.4 (-54.5 to -34.3)**	**49 851 (35 575 to 65 473)**	**67 (48 to 88)**	**-36.3 (-39.8 to -32.9)**	**179 864 (149 240 to 215 553)**	**237 (198 to 281)**	**-43.9 (-50.5 to -34.8)**
Botswana	2 883 (1 836 to 4 810)	133 (89 to 211)	-45.3 (-57.5 to -21.9)	1 389 (979 to 1 842)	63 (45 to 84)	-33.6 (-39.7 to -26.5)	4 271 (3 090 to 6 338)	196 (146 to 279)	-42.0 (-52.0 to -24.8)
Lesotho	5 161 (3 813 to 6 533)	281 (207 to 351)	-4.0 (-44.7 to 37.9)	1 554 (1 110 to 2 007)	84 (61 to 109)	-10.7 (-16.9 to -4.1)	6 715 (5 259 to 8 139)	365 (286 to 438)	-5.7 (-37.9 to 25.0)
Namibia	3 485 (2 550 to 5 055)	158 (117 to 225)	-47.1 (-58.4 to -28.7)	1 577 (1 119 to 2 060)	75 (53 to 98)	-39.0 (-44.8 to -32.7)	5 063 (3 952 to 6 763)	233 (185 to 307)	-44.7 (-53.5 to -32.2)
South Africa	85 024 (63 417 to 109 871)	154 (116 to 197)	-56.2 (-62.1 to -40.5)	33 042 (23 471 to 44 053)	60 (42 to 80)	-46.7 (-50.5 to -42.4)	118 066 (94 277 to 146 418)	214 (172 to 263)	-53.9 (-58.8 to -42.1)
Swaziland	2 453 (1 797 to 3 188)	235 (174 to 303)	-20.8 (-42.0 to 6.8)	728 (523 to 942)	73 (53 to 96)	-31.1 (-36.3 to -25.5)	3 181 (2 506 to 3 975)	308 (245 to 380)	-23.5 (-40.1 to -3.6)
Zimbabwe	31 007 (22 845 to 39 267)	224 (162 to 280)	10.0 (-28.9 to 48.2)	11 561 (8 129 to 14 999)	91 (64 to 116)	33.7 (24.8 to 43.7)	42 568 (33 876 to 51 059)	314 (250 to 378)	15.9 (-14.8 to 44.6)
**Western sub-Saharan Africa**	**628 061 (404 048 to 828 532)**	**130 (90 to 163)**	**-39.5 (-52.2 to -16.0)**	**218 067 (154 009 to 285 972)**	**62 (44 to 80)**	**-22.9 (-27.4 to -17.8)**	**846 128 (626 568 to 1 058 581)**	**192 (151 to 230)**	**-34.9 (-45.0 to -18.4)**
Benin	14 679 (9 462 to 21 589)	125 (83 to 173)	-44.7 (-59.7 to -21.6)	6 584 (4 667 to 8 676)	71 (51 to 92)	-21.3 (-27.2 to -15.7)	21 263 (15 783 to 28 334)	196 (152 to 250)	-38.0 (-49.2 to -21.4)
Burkina Faso	46 090 (31 725 to 64 509)	187 (142 to 245)	-38.0 (-52.5 to -10.1)	12 614 (8 810 to 16 382)	75 (53 to 96)	-22.5 (-28.2 to -16.6)	58 704 (44 368 to 78 088)	262 (213 to 325)	-34.2 (-46.2 to -13.8)
Cameroon	29 910 (17 468 to 41 105)	117 (67 to 156)	-40.1 (-59.0 to -13.1)	14 727 (10 324 to 19 199)	63 (45 to 82)	-23.4 (-28.8 to -17.8)	44 637 (32 046 to 57 166)	180 (130 to 224)	-35.1 (-48.5 to -16.7)
Cape Verde	238 (197 to 306)	47 (39 to 61)	-58.0 (-71.0 to -32.2)	230 (163 to 307)	43 (31 to 58)	-38.0 (-44.9 to -30.0)	468 (384 to 574)	90 (74 to 112)	-50.3 (-61.2 to -34.8)
Chad	35 354 (19 991 to 54 963)	192 (123 to 289)	-15.9 (-38.1 to 18.0)	8 982 (6 292 to 11 869)	79 (56 to 102)	-7.2 (-12.9 to -1.0)	44 336 (28 559 to 63 550)	271 (196 to 370)	-13.5 (-29.8 to 10.6)
Cote d'Ivoire	42 744 (32 208 to 56 048)	170 (133 to 213)	-32.8 (-48.2 to -5.5)	15 907 (11 147 to 20 926)	74 (52 to 95)	-12.1 (-17.9 to -6.3)	58 651 (47 190 to 72 430)	244 (203 to 289)	-27.6 (-40.5 to -6.7)
The Gambia	2 427 (1 730 to 3 592)	124 (90 to 169)	-38.5 (-54.3 to -10.1)	1 099 (775 to 1 451)	62 (45 to 80)	-18.7 (-24.9 to -12.7)	3 526 (2 736 to 4 704)	186 (148 to 235)	-33.1 (-45.1 to -13.0)
Ghana	30 035 (23 456 to 40 722)	111 (90 to 141)	-31.6 (-46.8 to -3.9)	15 729 (11 022 to 20 453)	58 (41 to 75)	-27.2 (-33.0 to -20.8)	45 764 (37 674 to 57 815)	169 (142 to 204)	-30.1 (-41.2 to -12.5)
Guinea	20 000 (12 647 to 28 278)	161 (106 to 221)	-46.6 (-62.8 to -15.6)	7 321 (5 184 to 9 639)	76 (54 to 99)	-23.5 (-29.3 to -17.5)	27 322 (19 703 to 36 079)	238 (180 to 305)	-40.9 (-54.4 to -18.1)
Guinea-Bissau	2 737 (1 886 to 3 678)	154 (104 to 200)	-48.1 (-63.3 to -19.5)	1 125 (800 to 1 491)	72 (51 to 93)	-26.5 (-32.3 to -20.9)	3 862 (2 975 to 4 882)	225 (174 to 276)	-42.8 (-56.0 to -21.2)
Liberia	4 618 (2 846 to 7 048)	105 (65 to 158)	-60.4 (-75.2 to -19.5)	2 578 (1 812 to 3 426)	64 (46 to 84)	-22.4 (-28.3 to -16.1)	7 196 (5 232 to 9 682)	169 (124 to 226)	-51.3 (-64.9 to -20.5)
Mali	52 541 (28 387 to 82 644)	198 (122 to 303)	-39.2 (-58.1 to -4.4)	10 282 (7 275 to 13 531)	66 (48 to 86)	-30.6 (-36.4 to -24.2)	62 823 (38 378 to 92 636)	265 (185 to 368)	-37.2 (-52.7 to -13.4)
Mauritania	3 240 (2 059 to 5 187)	87 (57 to 131)	-45.8 (-61.7 to -16.3)	1 920 (1 353 to 2 533)	60 (43 to 78)	-30.4 (-35.7 to -24.2)	5 160 (3 787 to 7 298)	147 (111 to 198)	-40.4 (-51.9 to -21.5)
Niger	42 873 (23 964 to 70 502)	166 (106 to 293)	-53.8 (-71.0 to -13.0)	11 996 (8 477 to 15 770)	78 (55 to 101)	-23.5 (-28.8 to -17.8)	54 869 (35 453 to 81 918)	245 (177 to 376)	-47.0 (-62.9 to -16.6)
Nigeria	262 202 (125 004 to 413 767)	107 (58 to 155)	-40.4 (-55.9 to -8.1)	89 386 (63 077 to 117 699)	55 (39 to 71)	-24.2 (-30.9 to -16.8)	351 588 (215 599 to 497 642)	161 (111 to 211)	-35.7 (-48.5 to -14.8)
Sao Tome and Principe	49 (34 to 91)	27 (19 to 45)	-50.5 (-64.9 to -14.7)	110 (78 to 144)	62 (45 to 81)	-11.0 (-17.9 to -3.6)	160 (121 to 210)	89 (69 to 112)	-28.1 (-38.4 to -12.8)
Senegal	19 005 (12 874 to 29 776)	132 (90 to 201)	-38.0 (-56.9 to -4.0)	8 798 (6 183 to 11 653)	72 (52 to 95)	-20.2 (-25.4 to -14.9)	27 803 (20 985 to 39 600)	204 (158 to 282)	-32.7 (-47.1 to -10.0)
Sierra Leone	12 553 (6 620 to 18 148)	153 (88 to 210)	-48.3 (-65.5 to -16.5)	4 412 (3 105 to 5 793)	67 (48 to 86)	-23.4 (-29.3 to -17.7)	16 964 (11 260 to 22 861)	220 (156 to 279)	-42.7 (-57.3 to -18.8)
Togo	6 761 (4 277 to 9 254)	99 (63 to 133)	-47.4 (-62.2 to -23.7)	4 264 (3 015 to 5 639)	65 (47 to 85)	-22.2 (-27.9 to -15.3)	11 025 (8 367 to 13 890)	164 (126 to 202)	-39.6 (-50.6 to -23.8)
